# Probing the Boundaries between Lewis‐Basic and Redox Behavior of a Parent Borylene

**DOI:** 10.1002/chem.202103256

**Published:** 2021-11-11

**Authors:** Merle Arrowsmith, Sara Endres, Myron Heinz, Vincent Nestler, Max C. Holthausen, Holger Braunschweig

**Affiliations:** ^1^ Institute for Inorganic Chemistry Julius-Maximilians-Universität Würzburg Am Hubland 97074 Würzburg Germany; ^2^ Institute for Sustainable Chemistry & Catalysis with Boron Julius-Maximilians-Universität Würzburg Am Hubland 97074 Würzburg Germany; ^3^ Institut für Anorganische und Analytische Chemie Goethe-Universität Frankfurt am Main Max-von-Laue-Str. 7 60438 Frankfurt am Main Germany

**Keywords:** bond dissociation energies, borylene, group 13, Lewis adducts, redox processes

## Abstract

The parent borylene (CAAC)(Me_3_P)BH, **1** (CAAC=cyclic alkyl(amino)carbene), acts both as a Lewis base and one‐electron reducing agent towards group 13 trichlorides (ECl_3_, E=B, Al, Ga, In), yielding the adducts **1‐ECl_3_** and increasing proportions of the radical cation **[1]^•+^** for the heavier group 13 analogues. With boron trihalides (BX_3_, X=F, Cl, Br, I) **1** undergoes sequential adduct formation and halide abstraction reactions to yield borylboronium cations and shows an increasing tendency towards redox processes for the heavier halides. Calculations confirm that **1** acts as a strong Lewis base towards EX_3_ and show a marked increase in the B−E bond dissociation energies down both group 13 and the halide group.

## Graphical abstract

Electrophile or nucleophile: A cyclic alkyl(amino)carbene‐ and PMe_3_‐stabilized parent borylene acts both as a Lewis base and reducing agent towards group 13 trihalides (EX_3_, E=B, Al, Ga, In, X=F, Cl, Br, I), yielding borylene‐EX_3_ adducts and the boryl radical cation. Halide abstraction converts the borylene‐BX_3_ adducts to the unsymmetrical borylboronium species. Computations probe the bond dissociation energy and electronic nature of the B–E bonds in the Lewis adducts.

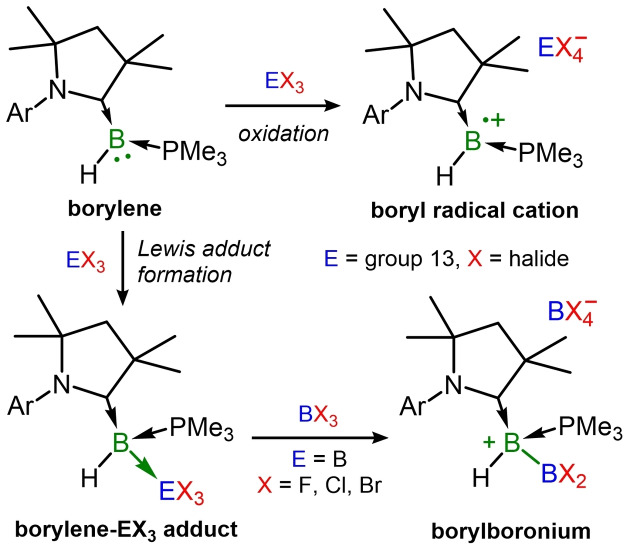

## Introduction

**Figure 1:**
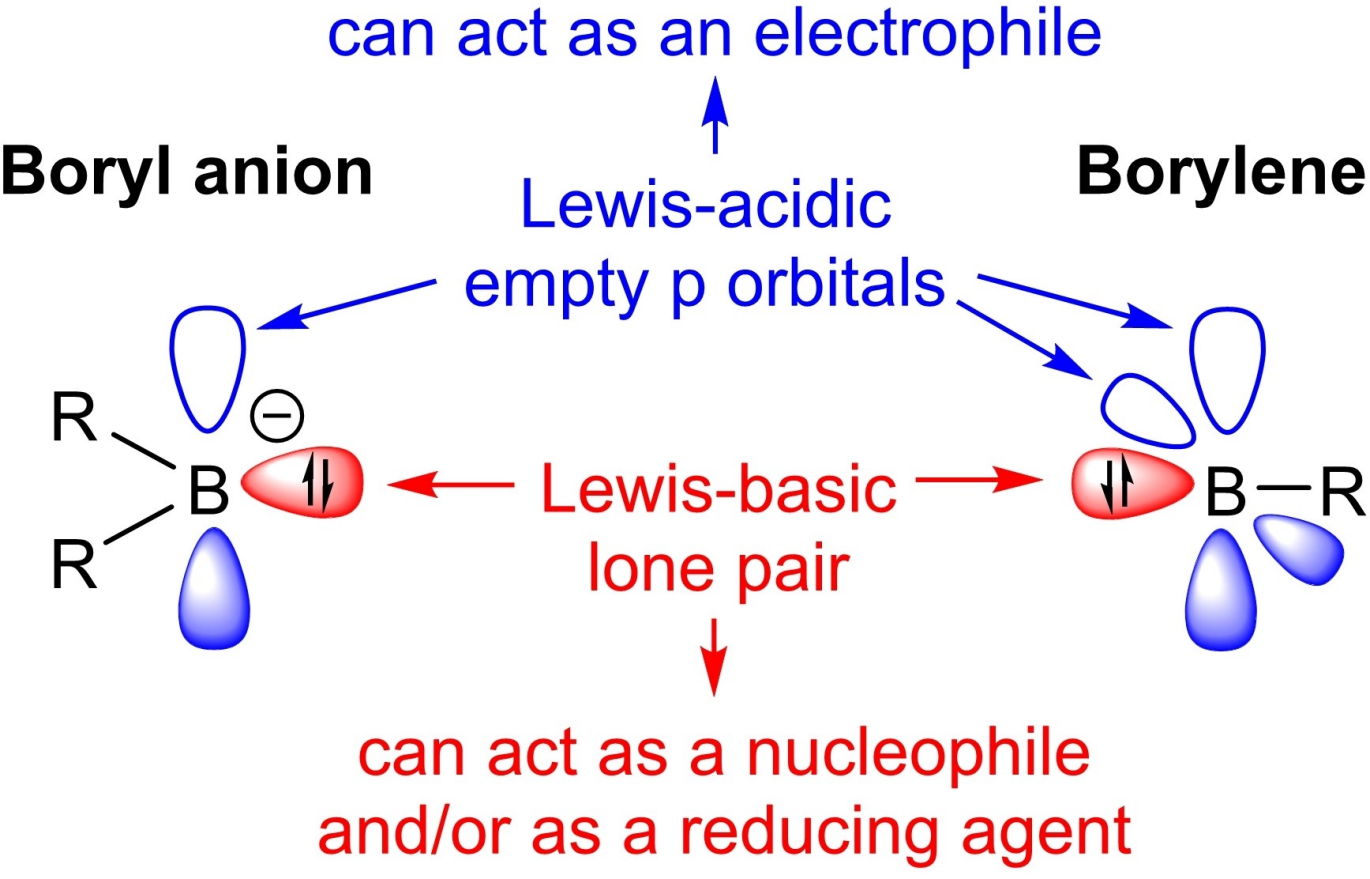
Schematic representation of the electronic structure of boryl anions and borylenes.

**Figure 2:**
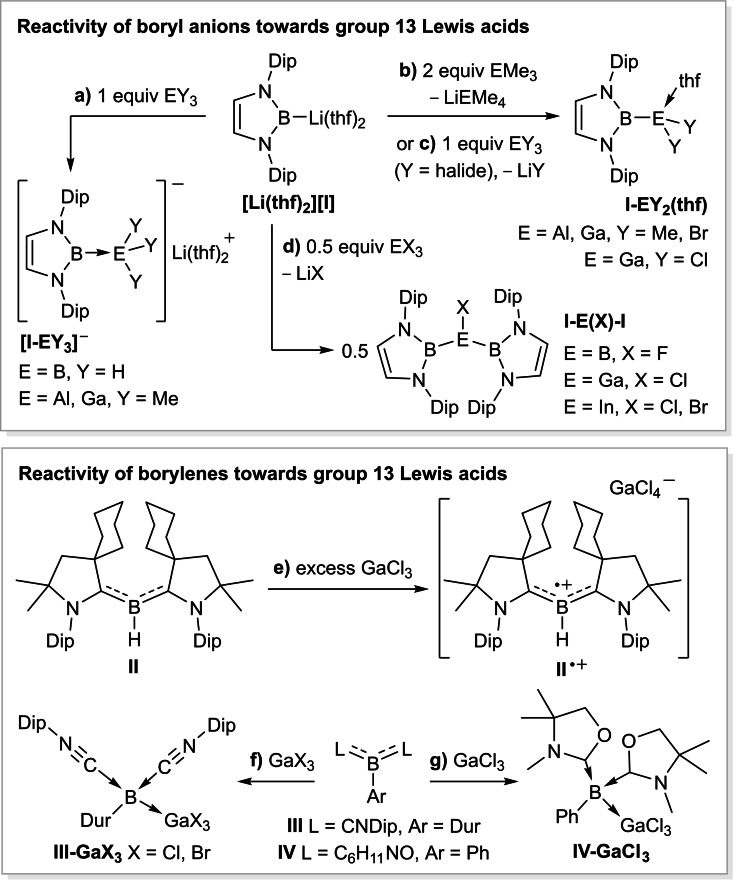
Reported reactivity of boryl anions and borylenes towards group 13 Lewis acids. Dip=2,6‐*i*Pr_2_C_6_H_3_; Dur=2,3,5,6‐Me_4_C_6_H.

With their formal lone pair at boron, boryl anions and borylenes are strong boron‐based nucleophiles, while their formally empty p orbital(s) also make them highly electrophilic (Figure 1). Since the isolation of the first boryl anion, **[I]** ^−^, by Yamashita in 2006^[1]^ and the first metal‐free doubly base‐stabilized borylene, **II**, by Bertrand in 2010^[2]^ (Figure 2) significant progress has been made in the targeted synthesis and the exploration of the reactivity of these unusually electron‐rich boron(I) compounds.

The nucleophilic character of boryl anions and borylenes towards main group Lewis acids can be exploited, in particular to generate new bonds between boron and elements of group 13 (E=B, Al, Ga, In). Yamashita's boryl anion, for example, coordinates as an anionic donor to BH_3_ and EMe_3_ (E=Al, Ga) to yield the corresponding borylborates, **[I‐BH_3_]** ^−^,^[4]^ and **[I‐EMe_3_]** ^−^,^[5]^ respectively (Figure 2a). In the presence of additional EMe_3_, however, methyllithium is abstracted from [**Li(thf)_2_][I‐EMe_3_]** to yield the neutral species **I‐EMe_2_(thf)** and the ionic by‐product, Li[EMe_4_] (Figure 2b).[^5^, ^6^] THF can then be abstracted from **I‐EMe_2_(thf)** either *in vacuo* or by adding further EMe_3_ as a Lewis acidic THF scavenger. With group 13 trihalides, **[I]** ^−^ systematically undergoes either single or double salt metathesis to yield the neutral dinuclear species **I‐EX_2_(thf)** (Figure 2c)^[7]^ or the trinuclear species **I‐E(X)‐I** (Figure 2d),[^7^, ^8^] which display electron‐sharing covalent B−E bonds. Similarly, an unsaturated analogue of **[I]** ^−^ undergoes salt metathesis with B(OMe)_3_ to yield the corresponding unsymmetrical 1,1‐dialkoxy‐2,2‐diaminodiborane(4).^[9]^

The reactivity of borylenes towards group 13 trihalides has not been so widely explored. Whereas Bertrand's hydroborylene **II** undergoes a one‐electron oxidation with GaCl_3_ to yield the corresponding boryl radical cation, **II** ^.**+**^ (Figure 2e),^[2]^ our group and that of Kinjo have shown that the doubly base‐stabilized arylborylenes **III** and **IV** react with gallium trihalides to form the Lewis adducts **III‐GaX_3_** (Figure 2f)^[10]^ and **IV‐GaCl_3_** (Figure 2g),^[11]^ respectively. From these reactions it becomes apparent that boron(I) species can interact with group 13 electrophiles both as bases, forming simple adducts, or as reducing agents.

To date, however, there has been no systematic study of Lewis‐basic versus redox reactivity of boron(I) compounds. In this work we present a highly reactive phosphine‐stabilized parent borylene and systematically investigate its reactivity towards the series of group 13 trichlorides (ECl_3_, E=B, Al, Ga, In) and of boron trihalides (BX_3_, X=F, Cl, Br, I). We show that trends in the selectivity of these reactions for either Lewis adduct formation and/or redox chemistry can be correlated to both the nature of the group 13 element and that of the halide. Computational investigations provide insights into the nature of the B–E bond in a series of borylene‐EX_3_ adducts.

## Results and Discussion

### Synthesis of borylene 1

**Scheme 1:**
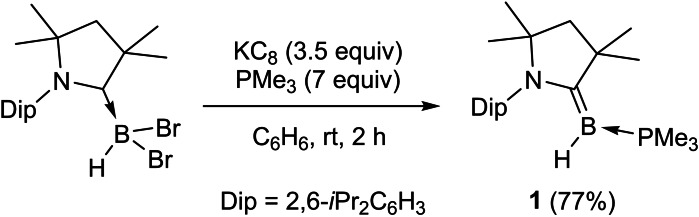
Synthesis of hydroborylene **1**.

The room‐temperature reduction of (CAAC)BHBr_2_ with 3.5 equiv. KC_8_ in benzene in the presence of 7 equiv. PMe_3_ yielded, after workup, the mixed‐base‐stabilized hydroborylene (CAAC)(Me_3_P)BH (**1**) as a yellow crystalline solid in good yield (77 %, Scheme 1). The ^11^B NMR spectrum of **1** presents an apparent triplet at −7.6 ppm, resulting from coupling to both the ^1^H and ^31^P nuclei (^1^ *J*_11B‐31P_≈^1^ *J*_11B‐1H_ ≈127 Hz). The ^11^B NMR shift of **1** is between that of the related cyanoborylene (CAAC)(PEt_3_)B(CN) (δ_11B_=−17.8 ppm)^[12]^ and chloroborylene (CAAC)(PEt_3_)BCl (δ_11B_=5.6 ppm).^[13]^ The corresponding ^1^H{^11^B} NMR B*H* resonance appears as a doublet at 1.72 (^2^ *J*_1H‐31P_ *=*19.8 Hz), while the ^31^P{^1^H} NMR spectrum displays a broad multiplet centered at −25.4 ppm.

**Figure 3:**
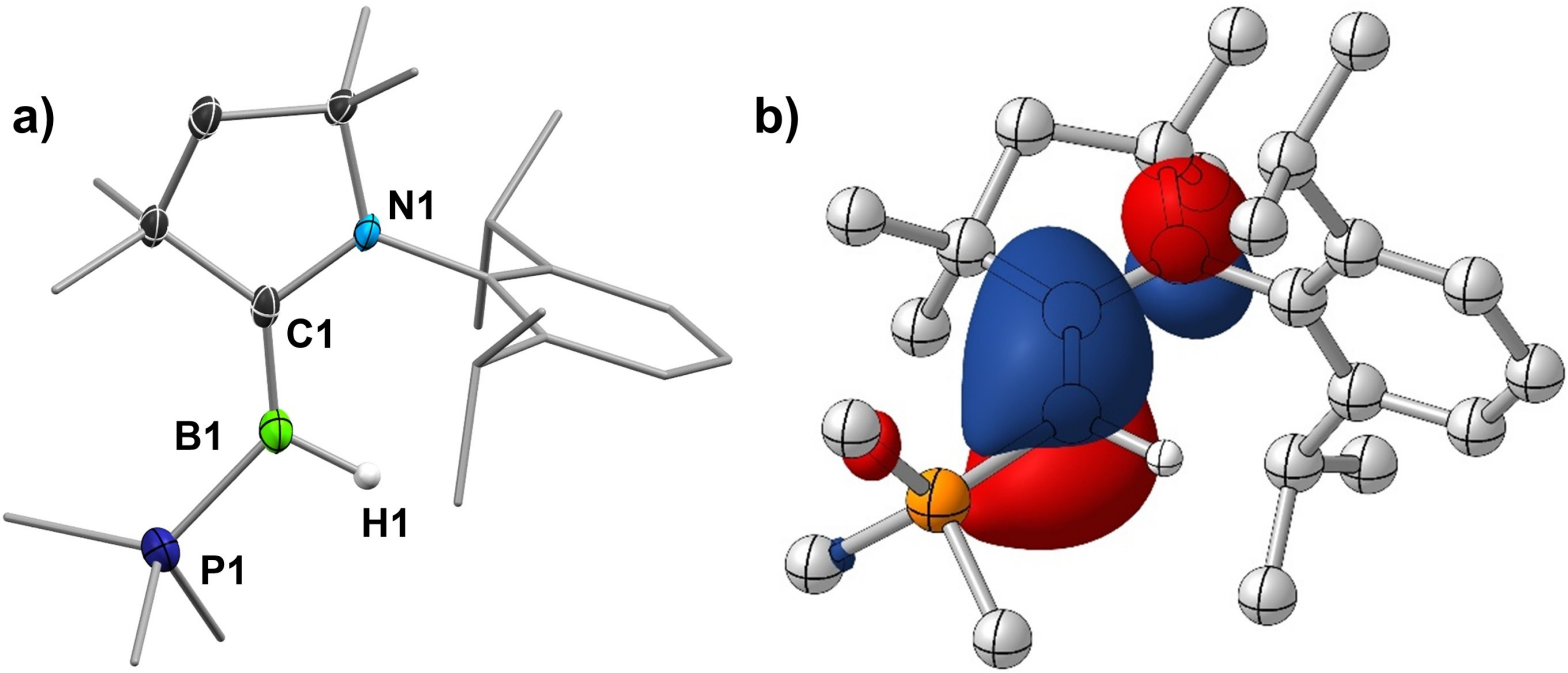
a) Crystallographically‐derived molecular structure of **1**. Atomic displacement ellipsoids drawn at 50 % probability level. Ellipsoids on ligand periphery and hydrogen atoms omitted for clarity. b) Plot of the HOMO of **1** (RI‐DSD‐BLYP‐D3BJ/def2‐QZVPP//PBEh‐3c level, isovalues ±0.05 a_0_^−3/2^). PBEh‐3c‐NPA charges: B1 −0.40, H1 0.00, C1 −0.08, N1 −0.56, P1 1.23.

The solid‐state structure of **1** (Figure 3a) shows a trigonal planar borylene center (Σ(∠B1) 359.93(12)°) bound to the CAAC ligand by a planar B=C double bond (B1–C1 1.454(3) Å; torsion angles N1‐C1‐B1‐H1 0.5(14)°, N1‐C1‐B1‐P1 179.54(14)°), similar to that in (CAAC)(PEt_3_)BCl (1.456(3) Å), and to the phosphine by a typical B–P single bond (1.871(2) Å). While a number of CAAC‐stabilized parent borylenes have been reported,[^2^, ^14^] this is the first phosphine‐stabilized example and the one presenting the least steric congestion at the borylene center, making it likely highly reactive. It therefore came as a surprise that **1** proved indefinitely stable in hydrocarbon solutions up to 100 °C and could be further purified without notable decomposition from traces of the hydrolysis by‐product (CAAC)BH_3_ by sublimation (110 °C, 10^3^ mbar). Density functional theory (DFT) calculations at the RI‐DSD‐BLYP‐D3BJ/def2‐QZVPP//PBEh‐3c level of theory (see Supporting Information for details) show that the HOMO, which represents the formal lone pair at boron, is slightly delocalized over the B–C π bond (B: 35 %, C: 21 %, see Table S6 in the Supporting Information) with a small antibonding contribution of the CAAC nitrogen p orbital (Figure 3b), similar to other (CAAC,PR_3_)‐stabilized borylenes.[^12^, ^13^] Natural population analysis (NPA) provides a calculated charge at boron of −0.40 (see legend of Figure 3), suggesting that **1** should be a strong boron‐centered nucleophile. Furthermore, the relatively small size of the hydride and PMe_3_ ligands afford sufficient space in the coordination sphere of boron for the coordination of Lewis acids.

### Synthesis and NMR‐spectroscopic characterization of adducts with group 13 trichlorides

**Scheme 2:**
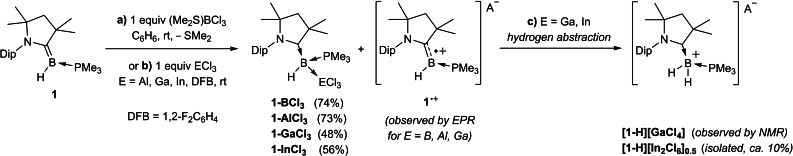
1 : 1 reactions between **1** and group 13 trichlorides.

**Figure 4:**
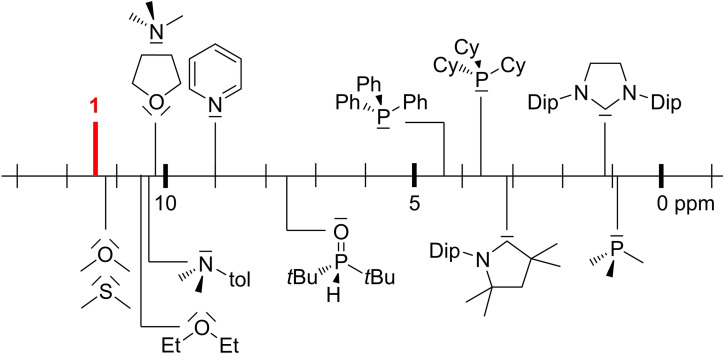
Comparison of the ^11^B NMR shift of **1‐BCl_3_** with literature‐known Lewis base adducts of BCl_3_ (tol=4‐MeC_6_H_4_).

We therefore set out to investigate the adduct formation of **1** with Lewis acidic group 13 trihalides. The room‐temperature reaction of borylene **1** with one equiv. (Me_2_S)BCl_3_ in benzene resulted in the crystallization of the colorless borylene‐borane adduct **1‐BCl_3_** in 74 % yield over the course of 30 min at room temperature (Scheme 2a).^[15]^ The ^11^B NMR spectrum of **1‐BCl_3_** displays two broad resonances at 11.4 and −21.4 ppm corresponding to the BCl_3_ and borylene moieties, respectively (Table 1). Given that the ^11^B NMR shift of Lewis base adducts of boranes is dependent on the overall electron‐donor strength of the Lewis base, a comparison with the ^11^B NMR shifts of literature‐known donor complexes of BCl_3_ (Figure 4)^[16]^ shows that borylene **1** is a comparatively weak Lewis base, similar to dimethyl ether and dimethyl sulfide. The ^31^P NMR shift of **1‐BCl_3_** at −10.9 ppm is significantly downfield‐shifted from that of **1** (δ_11B_=−25.4 ppm), and comparable to that of the [(CAAC)(PMe_3_)BH_2_]^+^ cation, **[1‐H]^+^** (δ_31P_=−10.6 ppm).^[17]^ In the ^1^H{^11^B} NMR spectrum the B*H* resonance appears at 1.80 ppm as a broad doublet coupling to the neighboring phosphorus nucleus (^2^ *J*_1H‐31P_=12.3 Hz), while the CAAC ligand resonances are all split due to the presence of the chiral borylene center and the hindered rotation around the B−C_CAAC_ bond.

**Table 1:** ^11^B and ^31^P NMR‐spectroscopic shifts of **1** and **1‐ECl_3_**.

Sample	δ_11B_ [ppm]	δ_31P_ [ppm]
**1**	−7.6 (t, ^1^ *J*_HB_≈^1^ *J*_PB_≈127 Hz)	−25.4 (m)
**1‐BCl_3_**	13.4 (br, *B*Cl_3_), −21.4 (br, *B*H)	−10.9 (m)
**1‐AlCl_3_**	−26.2 (br)	−12.9 (m)
**1‐GaCl_3_**	−25.2 (br t, ^1^ *J*_HB_≈^1^ *J*_PB_≈81 Hz)	−11.6 (m)
**1‐InCl_3_**	−24.6 (br t, ^1^ *J*_HB_≈^1^ *J*_PB_≈96 Hz)	−12.4 (m)

Similarly, the 1 : 1 reaction between **1** and AlCl_3_ in *o*‐difluorobenzene (DFB) afforded the borylene‐alane adduct **1‐AlCl_3_** as a colourless solid in 73 % yield (Scheme 2b). **1‐AlCl_3_** shows a broad ^11^B NMR resonance at −26.2 ppm, 5 ppm upfield of the borylene resonance of **1‐BCl_3_**, a ^31^P NMR multiplet at −12.9 ppm and a very broad ^27^Al NMR resonance at 125 ppm (fwmh ≈1400 Hz), in the region of four‐coordinate aluminium chlorides.^[18]^ While the 1 : 1 reactions with the heavier group 13 trichlorides, GaCl_3_ and InCl_3_, also resulted in the formation of the corresponding borylene‐gallane and ‐indane adducts (**1‐GaCl_3_**: δ_11B_=−25.2 ppm; **1‐InCl_3_**: δ_11B_=−24.6 ppm), these proved less selective (Scheme 2b).^[19]^ In both cases the NMR spectra of the reaction mixture showed the formation of varying amounts of the known **[1‐H]^+^** cation.[^17^, ^20^] For the InCl_3_‐based reaction single‐crystal X‐ray diffraction analysis confirmed the formation of the by‐product **[1‐H][In_2_Cl_6_]_0.5_** (see Figure S41 in the Supporting Information).^[21]^ The formation of **[1‐H]^+^** and the In_2_Cl_6_^2–^ counteranion, in which the indium centers are formally in the +2 oxidation state, points to a redox reaction between **1** and InCl_3_, followed by hydrogen radical abstraction by the **1** ^.**+**^ radical cation (Scheme 2c). Indeed EPR spectra of the 1 : 1 reactions of **1** with (Me_2_S)BCl_3_, AlCl_3_ and GaCl_3_, all showed the presence of the same radical species, presumably **[1][ECl_4_]** (E=B, Al, Ga),^[22]^ analogous to the radical cation obtained by Bertrand from the one‐electron oxidation of **II** with GaCl_3_ (Figure 2e).^[2]^

### Isolation of the boryl radical cation 1^•+^

**Figure 5:**
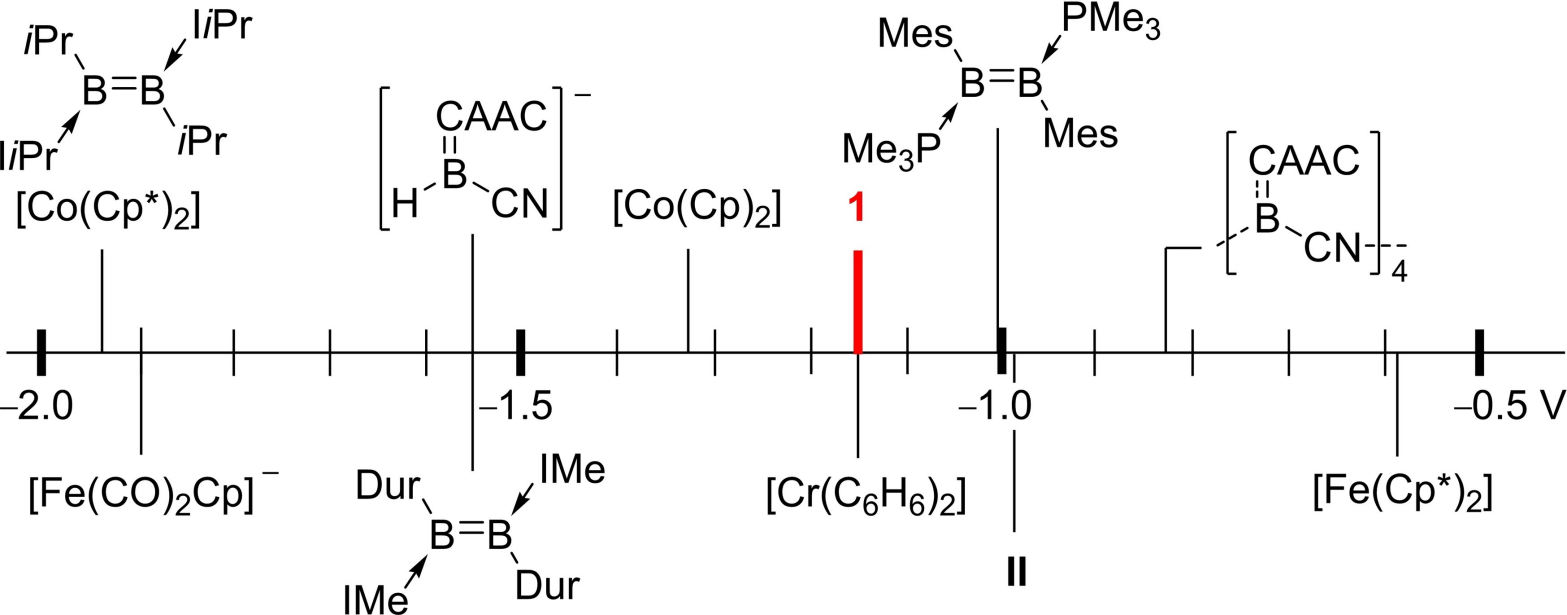
Comparison of the formal one‐electron oxidation potential of **1** (vs. Fc/Fc^+^, Fc=Cp_2_Fe with selected organometallic reducing agents and other boron(I) compounds. Cp=C_5_H_5_; Cp*=C_5_Me_5_; Dur=2,3,5,6‐Me_4_C_6_H; Mes=2,4,6‐Me_3_C_6_H_2_ ; I*i*Pr=1,3‐diisopropylimidazol‐2‐ylidene.

The cyclic voltammogram of **1** in in THF (0.1 M [*n*Bu_4_N][PF_6_]) shows a reversible oxidation wave at *E*_1/2_=−1.15 V and an irreversible one at *E*_pa_=+0.06 V (versus Fc/Fc^+^, Fc=(C_5_H_5_)_2_Fe, the former suggesting that a selective one‐electron chemical oxidation should be achievable. A comparison with commonly used organometallic reducing agents^[23]^ and other mono‐ and dinuclear boron(I) compounds (Figure 5)[^2^, ^12^, ^24^] shows that borylene **1** is a relatively mild one‐electron reducing agent, on a par with [Cr(C_6_H_6_)_2_] (*E*_1/2_=−1.15 V), but is significantly more reducing than borylene **II** (*E*_1/2_=−0.94 V)^[2]^ or our tetrameric cyanoborylene (*E*_1/2_=−0.83 V).^[12]^

**Scheme 3:**
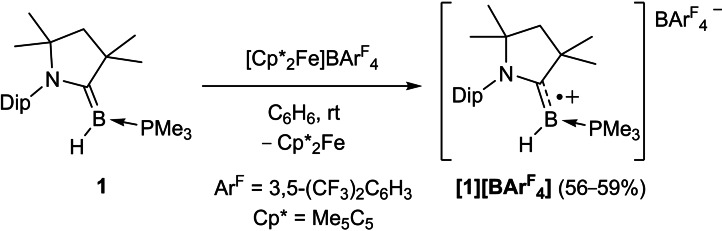
Independent synthesis of the boryl radical cation **1** ^.**+**^.

**Figure 6:**
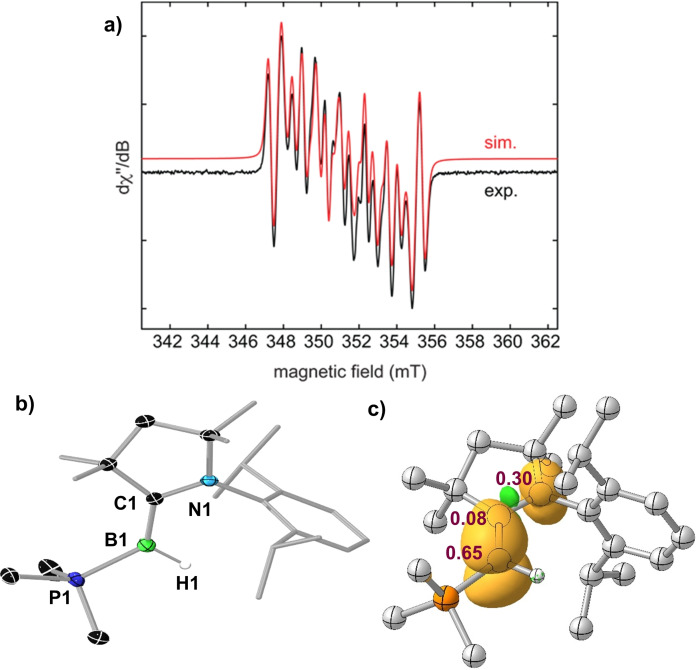
a) Experimental (black) and simulated (red) EPR spectrum of **1^.+^**. b) Crystallographically‐derived molecular structure of **1^.+^** (BAr^F^_4−_omitted for clarity). Thermal ellipsoids set at 50 % probability. Thermal ellipsoids of ligand periphery and hydrogen atoms omitted for clarity, except for boron‐bound H1. c) Plot of spin density of **1^.+^** with Mulliken atomic spin densities at the RI‐DSD‐BLYP‐D3BJ/def2‐QZVPP//PBEh‐3c level of theory (isovalues ±0.005 a_0_^−3^). PBEh‐3c‐NPA charges: B1 −0.01, H1 −0.01, C1 0.03, N1 −0.40, P1 1.20.

In order to confirm the formation of **1^•+^** in the reactions presented in Scheme 2, compound **1** was oxidized with [Cp*_2_Fe][BAr^F^_4_] (Cp*=C_5_Me_5_; Ar^F^=3,5‐bis(trifluoromethyl)phenyl, Scheme 3).^[25]^ **[1][BAr^F^**_**4**_ **]** was isolated as highly air‐ and moisture‐sensitive pale yellow crystals, the EPR spectrum of which was essentially identical to those recorded for the reactions shown in Scheme 2 (Figure 6a, see Figure S37 in the Supporting Information). The signal is unusually broad, spanning 105 G, and shows a unique splitting pattern. Simulation provides very large hyperfine coupling constants to the phosphorus and nitrogen nuclei (*a*(^31^P)=29.4 G; *a*(^14^N)=18.1 G; cf. **II** ^.**+**^: a(^14^N)=4.470 G),^[2]^ and coupling constants to the boron‐bound hydride (*a*(^1^H)=11.5 G) and boron nucleus (*a*(^11^B)=8.7 G), similar to those observed in **II** ^.**+**^ (*a*(^1^H)=11.447 G, *a*(^11^B)=6.432 G).^[2]^ The solid‐state structure of **1** ^.**+**^ shows a conformation analogous to **1** (Figure 6b), with a trigonal planar boron center (Σ∠_B1=_359.88(11)°), but significant elongation of the B1−C1 (1.508(2) Å) and shortening of the C1−N1 bonds (1.338(2) Å) compared to those **1** (B1−C1 1.454(3); C1−N1 1.408(2) Å), as expected upon oxidation. The B=C double bond remains planar as seen in the N1−C1−B1−H1 and N1−C1−B1−P1 torsion angles of 3.3(14) and 177.79(14), respectively. The NPA charge of −0.01 at boron is significantly less negative than that in **1** (−0.40), which is in line with the increase in oxidation state from +1 to +2. Furthermore, calculations show that the unpaired electron is delocalized over the B1‐C1‐N1 π system, with the majority of the Mulliken spin density (65 %) concentrated at boron and 30 % at the CAAC nitrogen atom. Among CAAC‐stabilized boron radicals this is the highest spin density at boron reported to date.^[26]^

### Structural analyses of borylene‐ECl_3_ adducts

**Figure 7:**
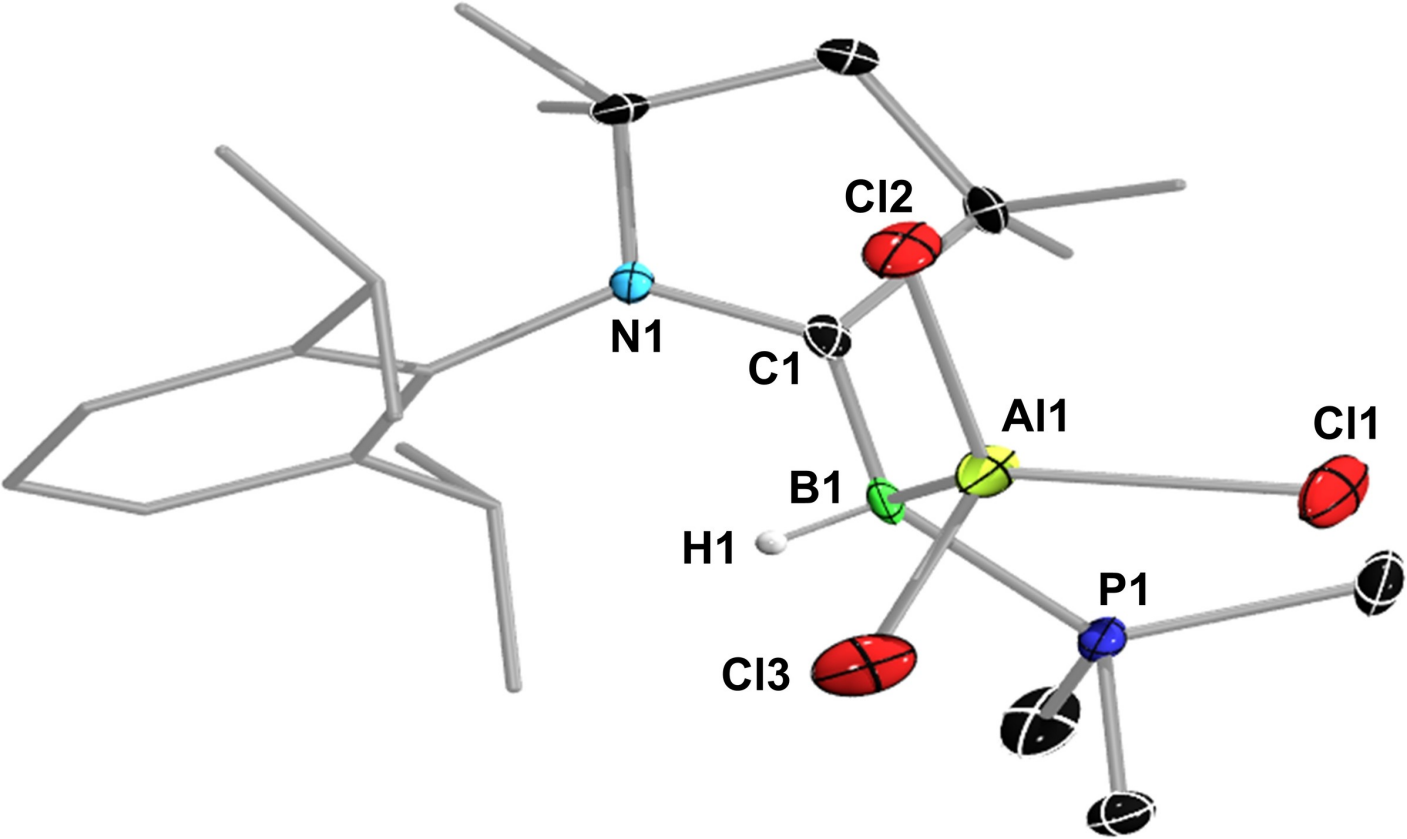
Crystallographically‐derived molecular structure of **1‐AlCl_3_**. Thermal ellipsoids set at 50 % probability. Thermal ellipsoids of ligand periphery and hydrogen atoms omitted for clarity, except for boron‐bound H1.

X‐ray crystallographic analyses were performed on all four **1‐ECl_3_** adducts (E=Al, Figure 7; E=B, Ga, In, see Figures S38–S40 in the Supporting Information). Relevant bond lengths and angles are listed in Table 2. All four compounds display a similar conformation, in which the B1‐bound hydride is oriented so as to minimize the (H1−B1−C1−N1) torsion angle (13 to 21°) and thereby the steric interaction between the Dip substituent and the B1‐bound PMe_3_ and ECl_3_ ligands. The B1−C1 (1.567(3) to 1.599(4) Å) and C1−N1 bond lengths (ca. 1.32 Å) denote B−C single and C−N double bonds, indicating that the CAAC ligand acts as a pure σ donor. The B−B bond length of 1.784(4) Å in **1‐BCl_3_** is similar to that of 1.797(4) Å in a bis(phosphine)‐stabilized borylene‐borane recently reported by our group.^[27]^ Complex **1‐AlCl_3_** is only the third borylene‐alane reported to date, its B−Al bond length of 2.191(2) Å being identical to that of our recently reported aminoborylene‐alane adduct (2.196(4) Å).^[28]^ The B−Ga bond length of 2.153(2) Å is only slightly shorter than that reported for **III‐GaCl_3_** and **IV‐GaX_3_** (ca. 2.17 Å).[^10^, ^11^] Complex **1‐InCl_3_** (B−In 2.314(2) Å) is, to our knowledge, the first reported borylene‐indane complex. It is noteworthy that the B−E bond lengths do not increase linearly down the group. Indeed the B−Al bond in **1‐AlCl_3_** (2.191(2) Å) is slightly longer than the B−Ga bond in **1‐GaCl_3_** (2.153(2) Å). This inverse trend has been observed previously in **I‐EMe_2_(thf)** (Figure 2b), in which the B−Al bonds (ca. 2.15 Å) are significantly longer than the B−Ga bonds (ca. 2.07 Å) owing to the d‐orbital contraction in gallium. This is also apparent in the quasi identical covalent radii of Al (*r*=1.21(4) Å) and Ga (*r*=1.22(3) Å).^[29]^

**Table 2:** Selected bond lengths [Å] and angles [°] of crystallographically characterized compounds.

	**1‐BCl_3_** ^[a]^	**1‐AlCl_3_** ^[b]^	**1‐GaCl_3_** ^[c]^	**1‐InCl_3_** ^[d]^	**[1‐BF_2_][BF_4_]** ^[a,e]^	**[1‐BCl_2_][BCl_4_]** ^[a]^	**4** ^[a]^	**[1‐I]I**
B1–C1	1.599(4)	1.567(3)	1.592(2)	1.579(2)	1.588(6), 1.602(6)	1.609(4)	1.620(4)	1.607(6)
B1–H1	1.14(3)	1.06(2)	1.06(2)	1.07(2)	1.18(5), 1.07(5)	1.12(4)	1.16(3)	1.12(5)
B1–P1	1.969(3)	1.927(2)	1.951(2)	1.939(2)	1.959(5), 1.942(5)	1.963(3)	1.974(4)^[f]^	1.977(5)
B1–E	1.784(4)	2.191(2)	2.153(2)	2.314(2)	1.733(7), 1.719(7)	1.709(4)	1.736(5)	2.290(5)^[g]^
N1–C1	1.318(3)	1.322(2)	1.320(2)	1.313(2)	1.305(5), 1.298(5)	1.313(4)	1.308(4)	1.305(6)
Torsion (H1‐B1‐C1‐N1)	21(1)	13(1)	15(1)	13(1)	17(3), 22(3)	22(2)	7(2)	–

[a] E=B. [b] E=Al. [c] E=Ga. [d] E=In. [e] Two crystallographically distinct molecules in the asymmetric unit. [f] B2–P1. [g] E=I.

### Reactivity of borylene 1 towards boron trihalides

**Scheme 4:**
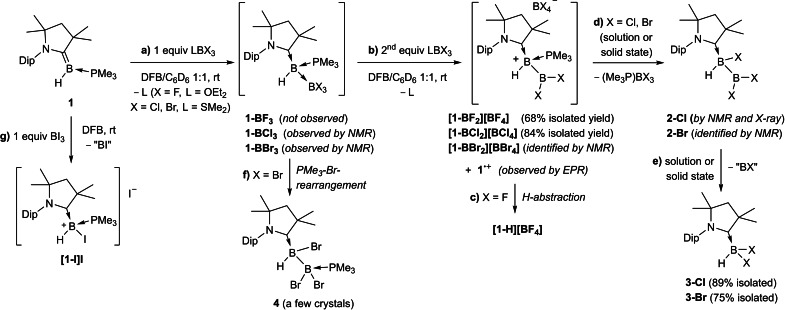
Divergent reactivity of **1** towards BX_3_ (X=F, Cl, Br, I).

Having determined these trends in the reactivity of ECl_3_ with borylene **1** (E=B, Al, Ga, In), we studied variations of the halide to identify further trends. Independent of the reaction conditions, combining **1** with (Et_2_O)BF_3_ resulted in a rapid 1 : 2 reaction,^[30]^ forming **[1‐BF_2_][BF_4_]** as the major product (ca. 80 %) and **[1‐H][BF_4_]** as the sole NMR‐active by‐product (Scheme 4a–c). Furthermore, the radical species **1^•+^** was detected by EPR spectroscopy. The formation of **[1‐BF_2_][BF_4_]** can be rationalized by fluoride ion abstraction from an initial **1‐BF_3_** adduct by a second BF_3_ equivalent (Scheme 4a,b). The fact that **1‐BF_3_** was never observed implies that fluoride abstraction occurs significantly faster than adduct formation in this case, presumably due to the much lower Lewis acidity of BF_3_ compared to BCl_3_ ^[31]^ and its high fluoride ion affinity.^[32]^

**Figure 8:**
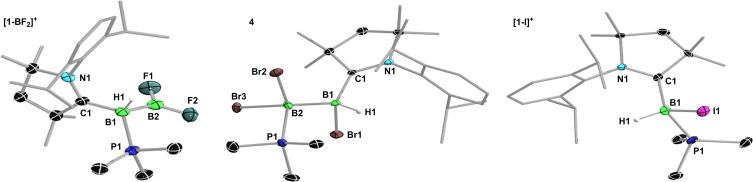
Crystallographically‐derived molecular structures of **[1‐BF_2_]^+^** (one of the two cations present in the asymmetric units), **4** and **[1‐I]^+^**. Thermal ellipsoids set at 50 % probability. Thermal ellipsoids of ligand periphery, the BF_4_^–^ counteranion of **[1‐BF_2_]^+^**, the iodide counteranion of **[1‐I]^+^** and hydrogen atoms omitted for clarity, except for boron‐bound H1.

The ^11^B NMR spectrum of **[1‐BF_2_][BF_4_]** shows two broad resonances at 34.6 (sp^2^‐B) and −30.8 (sp^3^‐B) ppm for the diboron cation and a sharp singlet at 0.1 ppm for the [BF_4_]^−^ anion. The ^19^F NMR spectrum displays two singlets at −38.5 and −138.7 for the B*F_2_* moiety and the [B*F_4_*]^−^ anion, respectively. The solid‐state structure of **[1‐BF_2_][BF_4_]** (Figure 8, Table 2) shows a sp^2^‐hybridized B2 center (Σ∠_B2_≈360°),^[33]^ with C1, B1, B2, F1 and F2 all belonging to the same plane (torsion angle (C1−B1−B2−F1) ca. 10°). The B−B bond (avg. 1.726 Å) is similar in length to those of other structurally characterized doubly base‐stabilized sp^2^‐sp^3^ borylboronium cations (avg. 1.73 Å).^[34]^

Independent of the reaction conditions the reaction of **1** with (Me_2_S)BBr_3_ or BBr_3_ proved highly unselective.^[35]^ While the formation of **1‐BBr_3_** (δ_11B_=−4.9 (br, *B*Br_3_), −20.1 (br, *B*H); δ_31P_=−12.1 (m) ppm, Scheme 4a) was observed when working with substoichiometric amounts of BBr_3_ at −70 °C, this adduct could not be isolated cleanly.^[36]^ As with (Et_2_O)BF_3_ the room temperature reaction always consumed two equiv. BBr_3_ and also resulted in a complex mixture of at least five boron‐containing species. Over the course of one day at room temperature, however, this mixture resolved into two major products, formed in a 1 : 1 ratio: (Me_3_P)BBr_3_ (δ_11B_=−4.4 (d, *J*_11B‐31P_=150 Hz) ppm; δ_31P_=−7.9 (m) ppm) and the known compound (CAAC)BHBr_2_, **3‐Br** (Scheme 4e).^[37]^ In order to elucidate the mechanism of this reaction, our attention turned to the analogous 1 : 2 reaction between **1** and (Me_2_S)BCl_3_. Carried out in a 1 : 1 DFB/benzene mixture, it resulted in the instant precipitation of **[1‐BCl_2_][BCl_4_]** (δ_11B_=75.8 (br, B*B*Cl_2_), 8.3 (s, BCl_4_^−^), −23.5 (br, *B*H) ppm; Scheme 4a,b). The solid‐state structure of **[1‐BCl_2_][BCl_4_]** (see Figure S42 in the Supporting Information, Table 2) resembles that of **[1‐BF_2_][BF_4_]**, with a slightly shorter B−B bond length of 1.709(4) Å, owing to the lower electronegativity of the chloride versus the fluoride ligands.

In solution and in the solid state at room temperature, isolated samples of **[1‐BCl_2_][BCl_4_]** converted overnight to a 1 : 1 mixture of the neutral sp^2^‐sp^3^ diborane (CAAC)BHCl(BCl_2_) (**2‐Cl**: δ_11B_=75.8 (br), −13.0 (br) ppm) and (Me_3_P)BCl_3_ (δ_11B_=3.1 (d, ^1^ *J*_11B‐31P_=164 Hz) ppm, Scheme 4d).^[38]^ The ^11^B NMR shifts of **2‐Cl** resemble those of the singly NHC‐stabilized adducts of B_2_Cl_4_ (δ_11B_=+69, −5 ppm), which are formed at low temperature and decompose upon warming.^[39]^ Diborane **2‐Cl** was also unstable both in solution and in the solid state, undergoing a B−B bond‐cleaving intramolecular chloride migration to yield the known compound (CAAC)BHCl_2_, **3‐Cl**,^[35]^ as the sole isolable product (Scheme 4e). The comparison of the ^11^B and ^31^P NMR spectra of **[1‐BCl_2_][BCl_4_]** and **2‐Cl** with those of the complex product mixture first obtained upon reacting **1** with (Me_2_S)BBr_3_ enabled the identification of **[1‐BBr_2_][BBr_4_]** (δ_11B_=74.5 (br, *B*Br_2_), −22.4 (br, *B*H), −23.2 (s, *B*Br_4_) ppm; δ_31P_=−9.9 (m) ppm) and **2‐Br** (δ_11B_=70.9 (br, *B*Br_2_), −9.0 (br, *B*H) ppm) as the major intermediates in the formation of **3‐Br**. Overall, the 1 : 2 reactions of **1** with (Me_2_S)BCl_3_ and (Me_2_S)BBr_3_ thus result in the two‐electron oxidation of **1** via one‐electron oxidation intermediates.

The only other crystalline product that was consistently isolated from the reaction of **1** with (Me_2_S)BBr_3_, albeit not in quantities sufficient for full characterization, was the unsymmetrical doubly base‐stabilized diborane **4** (δ_11B_=−4.8 (*B*Br_2_PMe_3_) and −15.3 (*B*HBr) ppm; δ_31P_=−11.7 (m) ppm), which results from the phosphine‐bromide rearrangement of **1‐BBr_3_** (Scheme 4f).^[40]^ The solid‐state structure of **4** (Figure 8, Table 2) confirms the migration of the PMe_3_ ligand to B2 and of one bromide to B1. The B–B bond length of 1.736(5) Å is significantly shorter than in **1‐BCl_3_** (1.784(4) Å). The phosphine and CAAC ligands are in an *anti* conformation, with a (P1−B2−B1−C1) torsion angle of 169.9(2)°. Compound **4**, which proved indefinitely stable at room temperature in solution, is the first structurally characterized example of a neutral (trihalo)hydrodiborane and a rare example of a neutral diborane stabilized by two different Lewis bases.^[41]^ It is structurally very similar to the carbene‐ and PMe_3_‐stabilized tetrabromodiborane reported by Kinjo and co‐workers.^[41b]^

Finally, the 1 : 1 reaction of **1** with BI_3_ in DFB proceeded very selectively, and independent of reaction temperature, to a single product displaying a broad ^11^B NMR resonance at −28.3 ppm and a ^31^P NMR multiplet at −14.3 ppm (Scheme 4g). After filtration from a small amount of intractable brown by‐product,^[42]^ recrystallization yielded single crystals of the two‐electron oxidation product **[1‐I]I** (Figure 8, Table 2). The fact that only one equivalent of BI_3_ is required and the PMe_3_ ligand remains bound to the boron center suggests a different reaction pathway from that of **1** with BBr_3_. Assuming that, here too, the Lewis adduct **1‐BI_3_** is formed first as an intermediate, the latter may be decomposing directly to **[1‐I]I** by iodide migration from B2 to B1 concomitant with B−B bond cleavage. Alternatively, the reaction may proceed via the one‐electron oxidation intermediate **1^•+^**, with subsequent iodine abstraction to yield **[1‐I]I**.

### Computational analysis of B–E bonding in 1‐EX_3_

In order to gain a deeper understanding of the bonding situation in the borylene‐EX_3_ adducts, B−E bond dissociation energies (BDEs) for the isolated **1‐ECl_3_** adducts (E=B, Al, Ga, In) and the putative **1‐BX_3_** adducts (X=F, Br, I) were calculated at the BP86‐D3BJ/TZ2P//PBEh‐3c and the improved double hybrid RI‐DSD‐BLYP−D3BJ/def2‐QZVPP//PBEh‐3c levels of theory (see details in the Supporting Information). The calculated B−E bond lengths match those of the solid‐state structures closely (within 1.5 to 2 %), including B−Al being slightly longer than B−Ga (Tables 2 and 3). The B−E BDEs at both levels of theory show similar trends, notwithstanding the typical overbinding by the BP86 functional. The comparison of the B−B BDEs of the putative **1‐BX_3_** adducts with the B−E BDEs of the isolated **1‐ECl_3_** adducts shows that the former would theoretically be stable enough for isolation if subsequent halide abstraction and/or redox processes could be prevented. In line with the general trend in Lewis acidities of the boron trihalides,^[43]^ calculated B–B BDEs in **1‐BX_3_** nearly double between **1‐BF_3_** and **1‐BCl_3_**, then increase more slowly upon descending the halide group further. The B–E BDEs of **1‐ECl_3_** increase substantially from BCl_3_ to InCl_3_, nearly doubling upon going from **1‐BCl_3_** and **1‐AlCl_3_**, then increasing more slowly upon descending group 13 further. Energy decomposition analysis (EDA) shows that this trend goes back to substantially diminished preparation energies ▵*E*_Prep_ (the energy necessary to deform the fragments from their individual equilibrium structures to the structures they assume in the respective adduct), whereas the corresponding interaction energies are essentially constant for all four **1‐ECl_3_** adducts. Deformation of the ECl_3_ fragments in particular dominates ▵*E*_Prep_. While the structural deformation, as measured by the sum of angles about the group 13 atom, is almost identical in all four cases, the bending potentials flatten substantially from BCl_3_ to InCl_3_ (see Figure S43 in the Supporting Information). Furthermore, while B−B bonding in **1‐BX_3_** is dominated by orbital interactions (54–60 %), the contribution of which increases upon descending the halide group, B−E bonding in **1‐ECl_3_** (E=Al, Ga, In) is dominated by electrostatic interactions (51–57 %), the contribution of which increases upon descending group 13 (see Tables S4 and S5 in the Supporting Information). This is in line with the increasing polarization of the B−B bond in **1‐BX_3_** for the lighter halides and of the B−E bond in **1‐ECl_3_** for the heavier group 13 elements.

**Table 3:** Bond dissociation, preparation and interaction energies according to energy decomposition analysis and B–E bond lengths for the **1‐EX_3_** adducts.

Sample	BDE^[a]^	BDE^[b]^	▵*E*_Int_^[b]^ [kcal mol^−1^]	▵*E*_Prep_^[b]^ [kcal mol^−1^]	B–E [Å]^[b]^
**1‐BCl_3_**	21.8	27.5	−97.9	68.5	1.81
**1‐AlCl_3_**	40.7	48.4	−91.2	39.6	2.23
**1‐GaCl_3_**	48.8	54.0	−97.7	42.5	2.19
**1‐InCl_3_**	56.4	61.8	−98.6	33.8	2.36
**1‐BF_3_**	11.3	16.1	−72.5	53.8	1.89
**1‐BBr_3_**	27.8	34.3	−108.3	70.6	1.81
**1‐BI_3_**	31.0	45.3	−122.0	73.6	1.81

[a] Based on improved RI‐DSD‐BLYP−D3BJ/def2‐QZVPP//PBEh‐3c calculations. [b] BP86‐D3BJ/TZ2P//PBEh‐3c.

## Conclusion

In this study we have shown that the (CAAC,PMe_3_)‐stabilized hydroborylene **1** offers an easily accessible, versatile platform for the systematic assessment of reactivity patterns of borylenes towards Lewis‐acidic group 13 trihalides, EX_3_. Depending on the nature of E and X the reactivity can be tuned either in favor of neutral Lewis adduct formation or one‐ and/or two‐electron redox processes.

With all group 13 trichlorides the 1 : 1 reaction yields the corresponding Lewis adduct **1‐ECl_3_** (E=B, Al, Ga, In) as the major product. The proportion of the radical cation by‐product **1^•+^**, resulting from the one‐electron oxidation of **1** by ECl_3_, increases upon descending group 13, as the corresponding reduction potential of ECl_3_ becomes more positive. The influence of the halide in these reactions becomes apparent in the reactions of **1** with BF_3_, BBr_3_ and BI_3_ sources. While it appears reasonable to assume initial formation of **1‐BX_3_** adducts, these species are too reactive to isolate. For X=F, fluoride abstraction by a second equivalent BF_3_ is significantly more rapid than **1‐BF_3_** adduct formation, leading to the stable borylboronium species **[1‐BF_2_][BF_4_]**. For X=Br, **1‐BBr_3_** also converts instantly to **[1‐BBr_2_][BBr_4_]** which is, however, highly unstable towards intramolecular ligand exchange and redox processes, ultimately resulting in the two‐electron oxidation of **1**. Finally, for X=I, only the product of the two‐electron redox reaction between **1** and BI_3_ is observed, thereby confirming the trend for increased redox processes down the group, as the B−X bond weakens.^[44]^

Based on the calculated BDEs, **1** acts as a typical strong Lewis base towards BX_3_. For the adducts of **1** with ECl_3_ the B−E BDEs increase down the group owing to successively weaker bending potentials of the ECl_3_ groups, which facilitates geometric distortion in the course of adduct formation.

Beyond the fundamental interest in the reactivity patterns of a Lewis‐basic borylene towards group 13 Lewis acids, and the study of borylene‐group 13 Lewis adduct bond enthalpies, the 1 : 2 reaction of **1** and BCl_3_ also provides a novel synthetic route towards an otherwise inaccessible, electron‐precise, unsymmetrical diborane, **2‐Cl**. Such species have become highly sought after as they display an intrinsic polarization of the B−B bond,^[45]^ making them significantly more reactive than commercially available symmetrical diboron reagents, in particular for uncatalyzed borylation and diboration reactions.^[46]^ Furthermore, the presence of halide substituents in the cationic borylboronium species, **[1‐BX_2_]^+^**, provides a potential handle either for subsequent ligand exchange by salt metathesis or for subsequent reduction chemistry.

## Experimental Section

**Crystallographic data**: Deposition Numbers 2107378 ([**1][BAr** ^**F**^_**4**_ **]**), 2107379 (**[1‐BCl**_**2**_ **][BCl**_**4**_ **]**), 2107380 (**1**), 2107381 (**1‐AlCl**_**3**_), 2107382 (**[1‐BF**_**2**_ **][BF**_**4**_ **]**), 2107383 (**[1‐I]I**), 2107384 (**1‐InCl**_**3**_ **⋅CHCl**_**3**_), 2107385 (**1‐BCl**_**3**_ **⋅C**_**6**_ **H**_**4**_ **F**_**2**_), 2107386 (**4**), 2107387 (**1‐InCl**_**3**_ **⋅C**_**6**_ **H**_**4**_ **F**_**2**_), 2107388 (**1‐GaCl**_**3**_), 2107389 (**[1‐H][In**_**2**_ **Cl**_**6**_ **]**_**0.5**_) and 2107390 (**1‐BCl**_**3**_ **⋅C**_**6**_ **H**_**6**_) contain the supplementary crystallographic data for this paper. These data are provided free of charge by the joint Cambridge Crystallographic Data Center and Fachinformationszentrum Karlsruhe Access Structures service.

## Conflict of interest

The authors declare no conflict of interest.

## Supporting information

As a service to our authors and readers, this journal provides supporting information supplied by the authors. Such materials are peer reviewed and may be re‐organized for online delivery, but are not copy‐edited or typeset. Technical support issues arising from supporting information (other than missing files) should be addressed to the authors.

Supporting Information
