# Supplementary material for: Probing the Boundaries between Lewis‐Basic and Redox Behavior of a Parent Borylene
Source: Chemistry. 2021 Nov 11;27(70):17660–8. doi: 10.1002/chem.202103256 (PMC9299150; doi:10.1002/chem.202103256)
Supplement: Supplementary file 1 — Supporting Information [file CHEM-27-17660-s001.pdf]

Chemistry–A European Journal

Supporting Information

Probing the Boundaries between Lewis-Basic and Redox Behavior of a Parent Borylene

Merle Arrowsmith, Sara Endres, Myron Heinz, Vincent Nestler, Max C. Holthausen, and Holger Braunschweig*

Contents

Methods and materials 2

Synthetic procedures 3

NMR spectra of isolated compounds 10

NMR-spectroscopic monitoring of the decomposition of **[1-BCl$_2$][BCl$_4$]** in solution 43

Cyclic voltammetry of **1** 44

EPR spectroscopy 45

X-ray crystallographic details 47

Computational details 55

References 64

Methods and materials

All manipulations were performed either under an atmosphere of dry argon or *in vacuo* using standard Schlenk line or glovebox techniques. Deuterated solvents were dried over molecular sieves and degassed by three freeze-pump-thaw cycles prior to use. All other solvents were distilled and degassed from appropiate drying agents. Solvents were stored under argon over activated 4 Å molecular sieves. NMR spectra were acquired on a Bruker Avance 400 or 500 NMR spectrometer. Chemical shifts (*δ*) are provided in ppm and internally referenced to the carbon nuclei ($^{13}C\{^{1}H\}$) or residual protons (^{1}H) of the solvent. Heteronuclei NMR spectra are referenced to external standards (^{11}B: $BF_3{\cdot}OEt_2$; ^{19}F: Cl_3CF, ^{27}Al: 1 M $Al(NO_3)_3$ in H_2O, ^{31}P: 85% H_3PO_4 in H_2O). Microanalyses (C, H, N, S) were performed on an Elementar vario MICRO cube elemental analyzer. High-resolution mass spectrometry data were obtained from a Thermo Scientific Exactive Plus spectrometer in LIFDI or ASAP mode.

Group 13 reagents were purchased from Sigma-Aldrich or Alfa Aesar and used as received. $(CAAC^{Me})BHBr_2$ ($CAAC^{Me}$ = 1-(2,6-di*iso*propylphenyl)-3,3,5,5-tetramethylpyrrolidin-2-ylidene)[1] and $[Cp^{*}_2Fe][BAr^F_4]$ (Cp* = pentamethylcyclopentadienyl, Ar^F = 3,5-bis(trifluoromethyl)phenyl)[2] were prepared following literature procedures. KC_8 was prepared by vigorously stirring potassium metal cut into small chunks (8.15 g, 208 mmol) and graphite dried overnight in the oven (20.0 g, 1.67 mol) at 200 °C under a gentle argon flow for 1 h.

Synthetic procedures

Synthesis of 1

In a 250 mL round-bottom Schlenk flask KC_8 (1.04 g, 7.66 mmol) was suspended in 50 mL benzene and PMe_3 (1.58 mL, 15.3 mmol) was added via syringe to the slurry. In a separate Schlenk flask $(CAAC^{Me})BHBr_2$ (1.00 g, 2.19 mmol) was suspended in 50 mL benzene and the mixture gently heated under stirring until complete dissolution of the reagent. The $(CAAC^{Me})BHBr_2$ solution was then added in small portions (ca. 1 mL) via a thin canula to the KC_8/PMe_3 slurry under vigorous stirring over a period of 5 minutes at rt. The resulting mixture was stirred at rt for another 2 h prior to removal of volatiles *in vacuo*. The solid residue was extracted with 2 x 50 mL hexane and the yellow filtrate concentrated to ca. 5 mL. Crystallization at –30 °C overnight yielded a first crop of yellow crystals of **1** (525 mg, 1.40 mmol, 64% yield). The filtrate was dried and recrystallized from pentane at –30 °C, yielding a second crop of **1** (total yield: 627 mg, 1.67 mmol, 77%). $^{1}H\{^{11}B\}$ NMR (500 MHz, C_6D_6, 297 K): δ = 7.26 (m, 3H, Dip-*H*), 3.55 (sept, 2H, ^{3}J = 6.8 Hz, *i*Pr-C*H*), 2.02 (s, 2H, C*H*$_2$), 1.72 (d, 1H, $^{2}J_{1H\text{-}31P}$ = 19.8 Hz, PB*H*), 1.56 (d, 6H, ^{3}J = 6.8 Hz, *i*Pr-C*H*$_3$), 1.51 (s, 6H, C(C*H*$_3$)$_2$), 1.42 (d, 6H, ^{3}J = 6.8 Hz, *i*Pr-C*H*$_3$), 1.31 (s, 6H, C(C*H*$_3$)$_2$), 0.83 (d, 9H, $^{2}J_{1H\text{-}31P}$ = 10.4 Hz, P(C*H*$_3$)$_3$) ppm. $^{13}C\{^{1}H\}$ NMR (126 MHz, C_6D_6, 297 K): δ = 175.4 (br, B*C*$_{CAAC}$), 150.9 (*o*-Dip-C), 139.9 (*i*-Dip-C), 126.9 (*p*-Dip-C), 124.3 (*m*-Dip-C), 64.2 (d, $^{4}J_{31P\text{-}13C}$ = 1.0 Hz, *C*(CH$_3$)$_2$), 58.1 (*C*H$_2$), 43.9 (d, $^{3}J_{31P\text{-}13C}$ = 2.8 Hz, *C*(CH$_3$)$_2$), 34.8 (d, $^{4}J_{31P\text{-}13C}$ = 2.2 Hz, C(*C*H$_3$)$_2$), 29.9 (C(*C*H$_3$)$_2$), 28.8 (*i*Pr-*C*H), 27.6, 24.4 (*i*Pr-*C*H$_3$), 17.4 (d, $^{1}J_{31P\text{-}13C}$ = 44.0 Hz, P*C*) ppm. ^{11}B NMR (160 MHz, C_6D_6, 297 K): δ = –7.6 (app. t, $^{1}J_{11B\text{-}31P} \approx {}^{1}J_{11B\text{-}1H} \approx$ 127 Hz). $^{31}P\{^{1}H\}$ NMR (202 MHz, C_6D_6, 297 K): δ = –25.4 (br m) ppm. LIFDI-HRMS calculated for $[C_{23}H_{42}BNP]^+$ = $[M + H]^+$ 374.3142; found: 374.3140.

Synthesis of 1-BCl$_3$

$(Me_2S)BCl_3$ (14 mg, 80 µmol) dissolved in 0.25 mL benzene were added to a solution of **2** (30 mg, 80 µmol) dissolved in 0.25 mL benzene at rt. A colorless precipitate formed rapidly. After 30 min at rt, the solution was decanted and the colorless solid washed with 0.2 mL pentane prior to drying *in vacuo*, yielding **1-BCl$_3$** as a colorless solid (29 mg, 59 µmol, 74%). **1-BCl$_3$** proved insoluble in most hydrocarbon solvents. In chlorinated solvents it remained stable in solution at rt for a few hours before slowly decomposing. $^{1}H\{^{11}B\}$ NMR (500 MHz, $CDCl_3$,

297 K): δ = 7.31 (t, 1H, ^3J = 7.5 Hz, *p*-Dip-*H*), 7.27 (dd, 1H, ^3J = 7.5 Hz, ^4J = 1.4 Hz, *m*-Dip-*H*), 7.16 (dd, 1H, ^3J = 7.5 Hz, ^4J = 1.4 Hz, *m*-Dip-*H*), 3.56 (br sept, 1H, *i*Pr-C*H*), 2.49 (sept, 1H, ^3J = 6.8 Hz, *i*Pr-C*H*), 2.10 (d, 1H, ^2J = 13.1 Hz, CH_2), 1.96 (d, 1H, ^2J = 13.1 Hz, CH_2), 1.87 (s, 3H, C(CH_3)$_2$), 1.80 (br d, 1H, $^2J_{1H-31P}$ = 12.3 Hz, PB*H*), 1.63 (d, 9H, $^2J_{1H-31P}$ = 10.8 Hz, P(CH_3)$_3$), 1.48, 1.29 (two s, 3H each, C(CH_3)$_2$), 1.27, 1.25, 1.21, 1.12 (four d, 3H each, ^3J = 6.8 Hz, *i*Pr-CH_3), 1.10 (s, 3H, C(CH_3)$_2$) ppm. ^{13}C{^{1}H} NMR (126 MHz, $CDCl_3$, 297 K): δ = 229.7 (BC_{CAAC}, detected by HMBC), 148.7 (*o*-Dip-*C*), 144.0 (d, $^5J_{31P-13C}$ = 1.4 Hz, *o*-Dip-*C*), 132.8 (*i*-Dip-*C*, detected by HMBC), 129.5 (*p*-Dip-*C*), 126.5, 124.7 (*m*-Dip-*C*), 79.6 (*C*(CH_3)$_2$), 53.9 (*C*(CH_3)$_2$), 51.5 (*C*H_2), 31.6 (C(CH_3)$_2$), 30.7 (d, $^4J_{31P-13C}$ = 1.5 Hz, C(*C*H_3)$_2$), 30.4, 30.1 (*i*Pr-*C*H_3), 29.6 (*i*Pr-*C*H), 29.0 (d, $^4J_{31P-13C}$ = 1.5 Hz, C(CH_3)$_2$), 28.8 (C(*C*H_3)$_2$), 28.3 (*i*Pr-*C*H), 24.4, 24.2 (*i*Pr-*C*H_3), 16.5 (d, ^1J = 40.2 Hz, P*C*H_3) ppm. ^{11}B NMR (160 MHz, $CDCl_3$, 297 K): δ = 13.4 (br, fwmh ≈ 700 Hz, B*B*Cl_3), –21.4 (br, fwmh ≈ 230 Hz, *B*HP) ppm. ^{31}P{^{1}H} NMR (202 MHz, $CDCl_3$, 297 K): δ = –10.9 (br m) ppm. Elemental analysis calculated for [$C_{23}H_{41}B_2Cl_3NP$] (M_w = 490.5): C 56.32, H 8.43, N 2.86; found: C 55.99, H 8.41, N 2.84%. Note: the compound could not be detected by HRMS in either LIFDI or ESI mode.

Synthesis of 1-AlCl$_3$

$AlCl_3$ (7.1 mg, 54 µmol) was combined with **1** (20 mg, 54 µmol) in *o*-difluorobenzene (DFB, 0.5 mL). The mixture turned green-brown and a small amount of dark precipitate formed. The mixture was filtered and a few drops of hexanes added to the pale yellow filtrate, which was left to crystallize at –30 °C. **1-AlCl$_3$** was isolated as colorless crystals (19 mg, 38 µmol, 73% yield). **1-AlCl$_3$** proved insoluble in most hydrocarbon solvents and decomposed in chlorinated solvents over a period of several weeks at rt. ^{1}H{^{11}B} NMR (500 MHz, $CDCl_3$): δ = 7.37 (t, 1H, ^3J = 7.7 Hz, *p*-Dip-*H*), 7.31 (d, 1H, ^3J = 7.7 Hz, *m*-Dip-*H*), 7.20 (d, 1H, ^3J = 7.7 Hz, *m*-Dip-*H*), 3.52 (sept, 1H, ^3J = 6.5 Hz, *i*Pr-C*H*), 2.65 (sept, 1H, ^3J = 6.5 Hz, *i*Pr-C*H*), 2.17 (d, 1H, ^2J = 12.9 Hz, CH_2), 2.03 (d, 1H, ^2J = 12.9 Hz, CH_2), 1.87 (s, 3H, C(CH_3)$_2$), 1.74 (d, 9H, $^2J_{1H-31P}$ = 10.9 Hz, P(CH_3)$_3$), 1.52 (s, 3H, C(CH_3)$_2$), 1.50 (br d, 1H, $^2J_{1H-31P}$ = 11.5 Hz, PB*H*), 1.35 (d, 3H, ^3J = 6.5 Hz, *i*Pr-CH_3), 1.32 (s + d, 6H, C(CH_3)$_2$ + *i*Pr-CH_3), 1.28 (d, 3H, ^3J = 6.5 Hz, *i*Pr-CH_3), 1.18 (d, 3H, ^3J = 6.5 Hz, *i*Pr-CH_3), 1.14 (s, 3H, C(CH_3)$_2$) ppm. ^{13}C{^{1}H} NMR (126 MHz, $CDCl_3$): δ = 224.3 (BC_{CAAC}, detected by HMBC), 148.9 (*o*-Dip-*C*), 144.6 (d, $^5J_{13C-31P}$ = 1.9 Hz, *o*-Dip-*C*), 132.9 (d, $^4J_{13C-31P}$ = 1.2 Hz, *i*-Dip-*C*), 129.4 (*p*-Dip-*C*), 125.9 (*m*-Dip-*C*), 124.5 (*m*-Dip-*C*), 77.2 (*C*(CH_3)$_2$, detected by HMBC), 52.3 (*C*(CH_3)$_2$), 51.5 (*C*H_2), 32.0 (C(*C*H_3)$_2$), 31.1 (d, $^4J_{13C-31P}$ = 1.4 Hz, C(*C*H_3)$_2$), 30.7 (*i*Pr-*C*H_3), 30.1 (C(*C*H_3)$_2$), 30.1 (*i*Pr-*C*H),

29.4 (iPr-CH), 28.3 (d, $^{5}J_{13C-31P}$ = 2.0 Hz, C(CH$_3$)$_2$), 28.1 (iPr-CH), 24.0 (iPr-CH$_3$), 23.7 (iPr-CH$_3$), 17.0 (d, $^{1}J_{13C-31P}$ = 43.3 Hz, P(CH$_3$)$_3$) ppm. ^{11}B NMR (160 MHz, CDCl$_3$): δ = –26.2 (br) ppm. ^{31}P{^{1}H} NMR (202 MHz, CDCl$_3$): δ = –12.9 (br m) ppm. ^{27}Al NMR (130 MHz, CDCl$_3$): δ = 125 (v. br, fwmh ≈ 1400 Hz) ppm. Elemental analysis calculated for [$C_{23}H_{41}AlBCl_3NP\cdot(C_6H_4F_2)_{0.75}$] ($M_w$ = 592.3): C 55.77, H 7.49, N 2.36; found: C 55.87, H 7.50, N 2.28. ASAP-HRMS calculated for [$C_{23}H_{41}AlBCl_4NP$]$^-$ = [M + Cl]$^-$: 542.1615, found: 542.1612.

Synthesis of 1-GaCl$_3$

GaCl$_3$ (9.4 mg, 54 µmol) was combined with **1** (20 mg, 54 µmol) in DFB (0.5 mL). The mixture turned bright green and a small amount of dark precipitate formed. The ^{11}B NMR spectrum directly after mixing showed the formation of four species (in varying amounts over several repeated reactions) at δ = –15.9 (br), –23.1 (br), –25.7 (br t, $^{1}J_{11B-31P} \approx {}^{1}J_{11B-1H}$ ≈ 81 Hz, **1-GaCl$_3$**) and –28.8 (q, $^{1}J_{11B-1H} \approx {}^{1}J_{11B-31P}$ ≈ 95 Hz, **[1-H]**$^+$). Furthermore, an EPR spectrum showed the presence of the radical cation **1**$^{\bullet+}$. The mixture was filtered and a few drops of hexanes were added to the filtrate, which was then left to crystallize at –30 °C. **1-GaCl$_3$** was isolated as pale yellow crystals (14 mg, 26 µmol, 48% yield). ^{1}H{^{11}B} NMR (500 MHz, CDCl$_3$): δ = 7.41 (t, 1H, ^{3}J = 7.7 Hz, p-Dip-H), 7.35 (dd, 1H, ^{3}J = 7.7 Hz, ^{4}J = 1.8 Hz, m-Dip-H), 7.22 (dd, 1H, ^{3}J = 7.7 Hz, ^{4}J = 1.8 Hz, m-Dip-H), 3.46 (sept, 1H, ^{3}J = 6.6 Hz, iPr-CH), 2.59 (sept, 1H, ^{3}J = 6.8 Hz, iPr-CH), 2.24 (d, 1H, ^{2}J = 13.0 Hz, CH_2), 2.09 (d, 1H, ^{2}J = 13.0 Hz, CH_2), 1.93 (s, 3H, C(CH_3)$_2$), 1.81 (d, 9H, $^{2}J_{1H-31P}$ = 11.2 Hz, P(CH_3)$_3$), 1.70 (d, 1H, $^{2}J_{1H-31P}$ = 9.3 Hz, PBH), 1.54 (s, 3H, C(CH_3)$_2$), 1.37 (d, 3H, ^{3}J = 6.6 Hz, iPr-CH_3), 1.35 (d, 3H, ^{3}J = 6.6 Hz, iPr-CH_3), 1.35 (s, 3H, C(CH_3)$_2$), 1.29 (d, 3H, ^{3}J = 6.8 Hz, iPr-CH_3), 1.21 (s, 3H, C(CH_3)$_2$), 1.18 (d, 3H, ^{3}J = 6.8 Hz, iPr-CH_3) ppm. ^{13}C{^{1}H} NMR (126 MHz, CDCl$_3$): δ = 223.7 (BC_{CAAC}, detected by HMBC), 148.6 (o-Dip-C), 144.0 (d, $^{5}J_{13C-31P}$ = 1.8 Hz, o-C_{Dip}), 132.0 (d, $^{4}J_{13C-31P}$ = 1.1 Hz, i-Dip-C), 129.9 (p-Dip-C), 126.3 (m-Dip-C), 124.6 (m-Dip-C), 79.1 (C(CH$_3$)$_2$), 53.2 (C(CH$_3$)$_2$), 50.9 (CH$_2$), 31.8 (C(CH$_3$)$_2$), 30.7 (s + d, C(CH$_3$)$_2$ + iPr-CH$_3$), 30.2 (iPr-CH$_3$), 29.7 (C(CH$_3$)$_2$), 29.6 (iPr-CH), 28.4 (d, $^{5}J_{13C-31P}$ = 2.0 Hz, C(CH$_3$)$_2$), 28.4 (iPr-CH), 23.9 (iPr-CH$_3$), 23.6 (iPr-CH$_3$), 16.3 (d, $^{1}J_{13C-31P}$ = 43.6 Hz, P(CH$_3$)$_3$) ppm. ^{11}B NMR (160 MHz, CDCl$_3$): δ = –25.2 (t, $^{1}J_{11B-31P} \approx {}^{1}J_{11B-1H}$ ≈ 81 Hz) ppm. ^{31}P{^{1}H} NMR (202 MHz, CDCl$_3$): δ = –11.6 (br m) ppm. Elemental analysis for [$C_{23}H_{41}BCl_3GaNP\cdot(C_6H_4F_2)$] ($M_w$ = 663.5): C 52.49, H 6.84, N 2.11; found: C 52.64, H 6.97, N 2.11%. ASAP-HRMS calculated

for $[C_{20}H_{32}BCl_4GaN]^-$ = [M – PMe_3 + Cl]$^-$: 508.0614; found: 508.0615; calculated for $[C_{23}H_{41}BCl_4GaNP]^-$ = [M + Cl]$^-$: 584.1056; found: 584.1061.

Synthesis of 1-$InCl_3$ and [1-H][In_2Cl_6]$_{0.5}$

$InCl_3$ (12 mg, 54 µmol) was combined with **1** (20 mg, 54 µmol) in DFB (0.5 mL). The ^{11}B NMR spectrum showed the formation of two species at δ = –25.1 (br t, $^1J_{11B\text{-}1H} \approx {}^1J_{11B\text{-}31P} \approx$ 96 Hz, **1-$InCl_3$**) and 28.4 (q, $^1J_{11B\text{-}1H} \approx {}^1J_{11B\text{-}31P} \approx$ 95 Hz, **[1-H][In_2Cl_6]$_{0.5}$**) in a ca. 1:1 ratio. The mixture was filtered and colorless crystals of **1-$InCl_3$** formed at rt overnight (18 mg, 30 µmol, 56% yield). The remaining solution was found to contain only **[1-H][In_2Cl_6]$_{0.5}$**. Isolated **1-$InCl_3$** proved virtually insoluble in all common organic solvents except in $CDCl_3$. In this solvent, however, it decomposed within 30 min at rt into an insoluble unidentified compound, which is why only ^1H, ^{11}B and ^{31}P NMR spectra of **1-$InCl_3$** could be recorded. $^1H\{^{11}B\}$ NMR (500 MHz, $CDCl_3$): δ = 7.45 (t, 1H, ^3J = 7.6 Hz, *p*-Dip-*H*), 7.36 (dd, 1H, ^3J = 7.6 Hz, ^4J = 1.7 Hz, *m*-Dip-*H*), 7.26 (dd, 1H, ^3J = 7.6 Hz, ^4J = 1.7 Hz, *m*-Dip-*H*), 3.22 (sept, 1H, ^3J = 6.6 Hz, *i*Pr-C*H*), 2.61 (sept, 1H, ^3J = 6.6 Hz, *i*Pr-C*H*), 2.28 (d, 1H, ^2J = 13.2 Hz, CH_2), 2.16 (d, 1H, ^2J = 13.2 Hz, CH_2), 1.94 (s, 3H, C(CH_3)$_2$), 1.92 (d, 9H, $^2J_{1H\text{-}31P}$ = 11.2 Hz, P(CH_3)$_3$), 1.79 (br d, 1H, $^2J_{1H\text{-}31P}$ = 6.8 Hz, PB*H*), 1.60 (s, 3H, C(CH_3)$_2$), 1.42 (d, 3H, ^3J = 6.6 Hz, *i*Pr-CH_3), 1.40 (s, 3H, C(CH_3)$_2$), 1.38 (d, 3H, ^3J = 6.6 Hz, *i*Pr-CH_3), 1.33 (d, 3H, ^3J = 6.6 Hz, *i*Pr-CH_3), 1.25 (s, 3H, C(CH_3)$_2$), 1.20 (d, 3H, ^3J = 6.6 Hz, *i*Pr-CH_3) ppm. ^{11}B NMR (128 MHz, $CDCl_3$): δ = –24.6 (br t, $^1J_{11B\text{-}1H} \approx {}^1J_{11B\text{-}31P} \approx$ 96 Hz) ppm. $^{31}P\{^1H\}$ NMR (161 MHz, $CDCl_3$): δ = –12.4 (m) ppm. Elemental analysis calculated for $[C_{23}H_{41}BCl_3InNP\cdot(C_6H_4F_2)_{4/3}]$ (M_w = 745.2): C 49.87, H 6.26, N 1.88; found: C 49.99, H 6.52, N 1.87%. ASAP-HRMS calculated for $[C_{23}H_{41}BCl_4InNP]^-$ = [M + Cl]$^-$: 630.0834; found: 630.0839.

Synthesis of [1][BAr^F_4]

<u>Method 1</u>: [Cp^*_2Fe][BAr^F_4] (127 mg, 107 µmol) was combined with **1** (40.0 mg, 107 µmol) in Et_2O at rt. The mixture was stirred for 5 min until complete disappearance of the green color of the Fe(III) starting material. The resulting pale yellow solution was stored for 2 days at –30 °C, resulting in crystallization of the yellow by-product [Cp^*_2Fe]. A second crop of [Cp^*_2Fe] was collected after another day at –30 °C. Hexane was added to the filtrate and the solution was stored for 1 week at –30 °C, yielding a first crop of colorless crystals of **[1][BAr^F_4]** (22.2

mg, 17.8 µmol, 17%). Two subsequent crops of crystals provided an overall isolated yield of **[1][BAr$^F_4$]** of 59% (78.0 mg, 63.1 µmol).

Method 2: Alternatively, stirring a suspension of [(Me_5C_5)$_2$Fe][BAr$^F_4$] (31.8 mg, 26.8 µmol) and **1** (10.0 mg, 26.8 µmol) in 0.5 mL benzene for 20 min at rt, until complete disappearance of the green color of the Fe(III) starting material, followed by the addition of 0.5 mL hexanes resulted in the formation of a yellow precipitate, which was dried in vacuo to yield **[1][BAr$^F_4$]** as a pale yellow solid (18.6 mg, 15.0 µmol, 56%). *Note:* elemental analysis data could not be obtained as our services can only measure probes with less than 15% fluorine content.

Synthesis of [1-BF$_2$][BF$_4$]

(Et_2O)BF_3 (21 mg, 0.14 mmol, 2.0 equiv) in 0.25 mL of DFB and **1** (27 mg, 72 µmol) in 0.25 mL of benzene were combined at rt. The solution rapidly turned colorless. Colorless crystals of **[1-BF$_2$][BF$_4$]** were obtained by leaving the solution undisturbed overnight at rt (25 mg, 49 µmol, 68%). The NMR spectra had to be recorded in a 1:1 mixture of DFB and C_6D_6 for solubility. ^{1}H{^{11}B} NMR (400 MHz, DFB/C_6D_6 1:1): δ = 7.09 (t, 1H, ^3J = 7.8 Hz, *p*-Dip-*H*), 6.98 (dd, 1H, ^3J = 7.8 Hz, ^4J = 1.6 Hz, *m*-Dip-*H*), 6.90 (dd, 2.83 ^3J = 7.8 Hz, ^4J = 1.6 Hz, *m*-Dip-*H*), 2.39 (sept, 2H, ^3J = 6.6 Hz, *i*Pr-C*H*), 1.91 (d, 1H, ^2J = 13.5 Hz, CH_2), 1.61 (d, 1H, ^2J = 13.5 Hz, CH_2), 1.41 (s, 3H, C(CH_3)$_2$), 1.35 (d, 9H, ^2J = 11.5 Hz, P(CH_3)$_3$), 1.31 (br, 1H, B*H*), 1.23 (overlapping s + d, 6H, C(CH_3)$_2$ + *i*Pr-CH_3), 1.03 (d, 3H, ^3J = 6.6 Hz, *i*Pr-CH_3), 0.98 (s, 3H, C(CH_3)$_2$), 0.94 (d, 3H, ^3J = 6.6 Hz, *i*Pr-CH_3), 0.90 (d, 3H, ^3J = 6.6 Hz, *i*Pr-CH_3), 0.78 (s, 3H, C(CH_3)$_2$) ppm. ^{13}C{^{1}H} NMR (126 MHz, $CDCl_3$): δ = 224.3 (BC_{CAAC}, detected by HMBC), 145.3 (*o*-Dip-*C*), 144.4 (d, $^5J_{31P\text{-}13C}$ = 1.2 Hz, *o*-Dip-*C*), 131.7 (*i*-Dip-*C*), 130.6 (*p*-Dip-*C*), 125.7, 125.4 (*m*-Dip-*C*), 81.2 (*C*(CH_3)$_2$), 53.3 (*C*(CH_3)$_2$), 50.2 (*C*H_2), 29.6, 29.5 (C(*C*H_3)$_2$), 29.4, 28.9 (*i*Pr-*C*H_3), 28.2, 27.9 (C(*C*H_3)$_2$), 27.8 (d, $^3J_{31P\text{-}13C}$ = 1.5 Hz, *C*(CH_3)$_2$), 23.3, 23.2 (*i*Pr-*C*H_3), 13.4 (d, $^1J_{31P\text{-}13C}$ = 45.2 Hz, P*C*H_3) ppm. ^{11}B NMR (128 MHz, DFB/C_6D_6 1:1): δ = 34.6 (br, B*B*F_2), 0.1 (s, *B*F_4), –30.8 (br, B*B*H) ppm. ^{19}F (376 MHz, DFB/C_6D_6 1:1): δ = –38.6 (s, 2F, BB*F$_2$*), –149.1 (s, 4F, B*F$_4$*) ppm. ^{31}P{^{1}H} NMR (161 MHz, DFB/C_6D_6 1:1): δ = –9.4 (m) ppm. ESI-HRMS calculated for [$C_{23}H_{41}B_2F_2NP$]$^+$ = [M]$^+$: 422.3125, found: 422.3134. *Note:* elemental analysis data could not be obtained as our services can only measure probes with less than 15% fluorine content.

Synthesis of [1-BCl$_2$][BCl$_4$]

(Me_2S)BCl_3 (19.2 mg, 0.107 mmol, 2.00 equiv.) was combined with **1** (20.0 mg, 53.6 µmol) in benzene (0.5 mL), causing the instant precipitation of **[1-BCl$_2$][BCl$_4$]** as a colorless solid, which was washed with 0.5 mL benzene and dried in vacuo (27.3 mg, 45.0 µmol, 84%). Colorless single crystals of **[1-BCl$_2$][BCl$_4$]** were obtained from a saturated DFB solution layered with hexanes and stored at –30 °C for 1 h. NMR spectra were recorded in a 10:1 mixture of C_6D_6 and DFB, the latter being required to solubilize **[1-BCl$_2$][BCl$_4$]**. In solution and in the solid state at rt **[1-BCl$_2$][BCl$_4$]** was not stable, undergoing intramolecular Cl-PMe$_3$ exchange to yield (CAAC)BCl(H)BCl$_2$ (**2-Cl**) and (Me_3P)BCl_3 (δ_{11B} = 75.8 (br, *B*Cl$_2$), 3.1 (d, $^1J_{11B\text{-}31P}$ = 173 Hz, *B*P), –13.0 (br, *B*H) ppm; δ_{31P} = –8.1 (br, B*P*) ppm), followed by decomposition of **2-Cl** to (CAAC)BHCl$_2$ (**3-Cl**, δ_{11B} = –4.5 (d, $^1J_{11B\text{-}1H}$ = 120 Hz) ppm, confirmed by single crystal X-ray crystallographic analysis).[1] As a result ^{13}C{^{1}H} NMR data for **[1-BCl$_2$][BCl$_4$]** and **2-Cl** could not be obtained. NMR data for **[1-BCl$_2$][BCl$_4$]**: ^{1}H{^{11}B} NMR (400 MHz, C_6D_6/DFB 10:1): δ = 7.03 (t, 1H, ^3J = 7.8 Hz, *p*-Dip-*H*), 6.94 (d, 1H, ^3J = 7.8 Hz, *m*-Dip-*H*), 6.82 (d, 1H, ^3J = 7.8 Hz, *m*-Dip-*H*), 3.53 (br s, 1H, B*H*), 2.52 (sept, 1H, ^3J = 6.3 Hz, *i*Pr-C*H*), 2.34 (sept, 1H, ^3J = 6.3 Hz, *i*Pr-C*H*), 2.09 (d, 1H, ^2J = 13.4 Hz, C*H*$_2$), 1.72 (d, 1H, ^2J = 13.4 Hz, C*H*$_2$), 1.62 (s, 3H, C(C*H*$_3$)$_2$), 1.46 (s, 3H, C(C*H*$_3$)$_2$), 1.38 (d, 9H, ^2J = 11.2 Hz, P(C*H*$_3$)$_3$), 1.27 (d, 3H, ^3J = 6.4 Hz, *i*Pr-C*H*$_3$), 1.03 (s, 3H, C(C*H*$_3$)$_2$), 0.97–1.00 (two overlapping d, 6H, *i*Pr-C*H*$_3$), 0.86 (d, 3H, ^3J = 6.4 Hz, *i*Pr-C*H*$_3$), 0.78 (s, 3H, C(C*H*$_3$)$_2$) ppm. ^{11}B NMR (128 MHz, C_6D_6/DFB 10:1): δ = 75.8 (br, fwmh ≈ 1020 Hz, *B*Cl$_2$), 8.3 (s, *B*Cl$_4$), –23.5 (br, fwmh ≈ 230 Hz, *B*H) ppm. ^{31}P{^{1}H} NMR (161 MHz, C_6D_6/DFB 10:1): δ = –12.7 (m) ppm. Elemental analysis calculated for [$C_{23}H_{41}B_3Cl_6NP\cdot(C_6H_6)_2$] ($M_w$ = 607.7): C 55.03, H 6.99, N 1.83; found: C 55.44, H 7.09, N 1.67%. ESI-HRMS calculated for [$C_{23}H_{41}B_2Cl_2NP$]$^+$ = [M]$^+$: 454.2534, found 454.2536.

Reaction of 1 with (Me$_2$S)BBr$_3$

(Me_2S)BBr_3 (33.5 mg, 107 mmol, 2.00 equiv.) was combined with **1** (20.0 mg, 53.6 µmol) in DFB (0.5 mL). ^{11}B and ^{31}P NMR-spectroscopic monitoring showed the instant formation of a complex mixture of **[1-BBr$_2$][BBr$_4$]** (δ(^{11}B) = 74.5 (br, *B*Br$_2$), –22.4 (br, *B*H), –23.2 (s, *B*Br$_4$) ppm; δ(^{31}P) = –9.9 (m) ppm), **2-Br** (δ(^{11}B) = 70.9 (br, *B*Br$_2$), –9.0 (br, *B*H) ppm), (Me_3P)BBr_3 (δ(^{11}B) = –14.4 (d, $J_{11B\text{-}31P}$ = 150 Hz) ppm; δ(^{31}P) = –7.9 (m) ppm) and the known compound (CAAC)BHBr$_2$, **3-Br** (confirmed by single crystal X-ray crystallographic analysis).[1] Overnight at room temperature the mixture resolved essentially to a 1:1 mixture of (Me_3P)BBr_3

and **3-Br**. Only one other very minor reaction product (< 5%), the unsymmetrical diborane (CAAC)B(H)(Br)$BBr_2(PMe_3)$, compound **4**, was systematically isolated from crystallization attempts at –30 °C in hexane/DFB mixtures as a handful of colorless crystals ($\delta(^{11}B)$ = –4.8 (br, *B*Br_2PMe_3), –15.3 (br, *B*H) ppm). Due to the very small amount of material collected each time no other analyses could be performed on **4**.

Synthesis of [1-I]I

BI_3 (21.0 mg, 53.5 µmol) was combined with **1** (20.0 mg, 53.5 µmol) in benzene (0.5 mL). An orange-colored oily phase rapidly separated at the bottom of the vial. After 10 min at rt the supernatant benzene solution was decanted and the oily phase dissolved in a few drops of *o*-difluorobenzene, layered with hexanes and left to crystallize at –30 °C. **[1-I]I** was isolated as pale yellow crystals (11.5 mg, 18.3 µmol, 34% yield). $^1H\{^{11}B\}$ NMR (400 MHz, $CDCl_3$): δ = 7.52 (t, 1H, ^3J = 7.8 Hz, *p*-Dip-*H*), 7.32-7.38 (m, 2H, *m*-Dip-*H*), 2.74 (sept, 1H, ^3J = 6.8 Hz, *i*Pr-C*H*), 2.46 (sept, 1H, ^3J = 6.6 Hz, *i*Pr-C*H*), 2.42 (d, 1H, ^2J = 13.4 Hz, CH_2), 2.36 (br d, 1H, $^2J_{1H\text{-}31P}$ = 10.1 Hz, PB*H*), 2.27 (d, 1H, ^2J = 13.4 Hz, CH_2), 2.01 (s, 3H, C(CH_3)$_2$), 1.98 (d, 9H, ^2J = 11.4 Hz, P(CH_3)$_3$),1.80 (s, 3H, C(CH_3)$_2$), 1.46 (s, 3H, C(CH_3)$_2$), 1.43 (d, 3H, ^3J = 6.6 Hz, *i*Pr-CH_3), 1.37-1.40 (two overlapping d, 6H, *i*Pr-CH_3), 1.36 (s, 3H, C(CH_3)$_2$), 1.22 (d, 3H, ^3J = 6.8 Hz, *i*Pr-CH_3) ppm. $^{13}C\{^1H\}$ NMR (126 MHz, $CDCl_3$): δ = 223.7 (BC_{CAAC}, detected by HMBC), 145.9 (*o*-Dip-*C*), 143.6 (*o*-Dip-*C*), 131.2 (*i*-Dip-*C*), 130.8 (*p*-Dip-*C*), 126.5 (*m*-Dip-*C*), 125.4 (*m*-Dip-*C*), 81.9 (*C*(CH_3)$_2$), 54.4 (*C*(CH_3)$_2$), 51.0 (*C*H_2), 35.5 (C(*C*H_3)$_2$), 30.7 (*i*Pr-*C*H_3), 30.3 (d, $^4J_{13C\text{-}31P}$ = 0.9 Hz, C(*C*H_3)$_2$), 29.8 (C(*C*H_3)$_2$), 29.0 (two s, iPr-*C*H), 28.5 (*i*Pr-*C*H_3), 28.4 (d, $^4J_{13C\text{-}31P}$ = 0.9 Hz, C(*C*H_3)$_2$), 24.5 (iPr-*C*H_3), 23.8 (*i*Pr-*C*H_3), 12.6 (d, $^1J_{13C\text{-}31P}$ = 46.8 Hz, P(*C*H_3)$_3$) ppm. ^{11}B NMR (128 MHz, $CDCl_3$): δ = –28.3 (br) ppm. $^{31}P\{^1H\}$ NMR (161 MHz, $CDCl_3$): δ = –14.3 (br m) ppm. Elemental analysis calculated for [$C_{23}H_{41}BI_2NP$] (M_w = 627.2): C 44.05, H 6.59, N 2.23; found: C 43.98, H 6.68, N 2.16%. HRMS-LIFDI calculated for $[C_{23}H_{41}BINP]^+ = [M]^+$: 500.2109, found: 500.2101.

NMR spectra of isolated compounds

Figure S1. $^{1}H\{^{11}B\}$ NMR spectrum of **1** in C_6D_6.

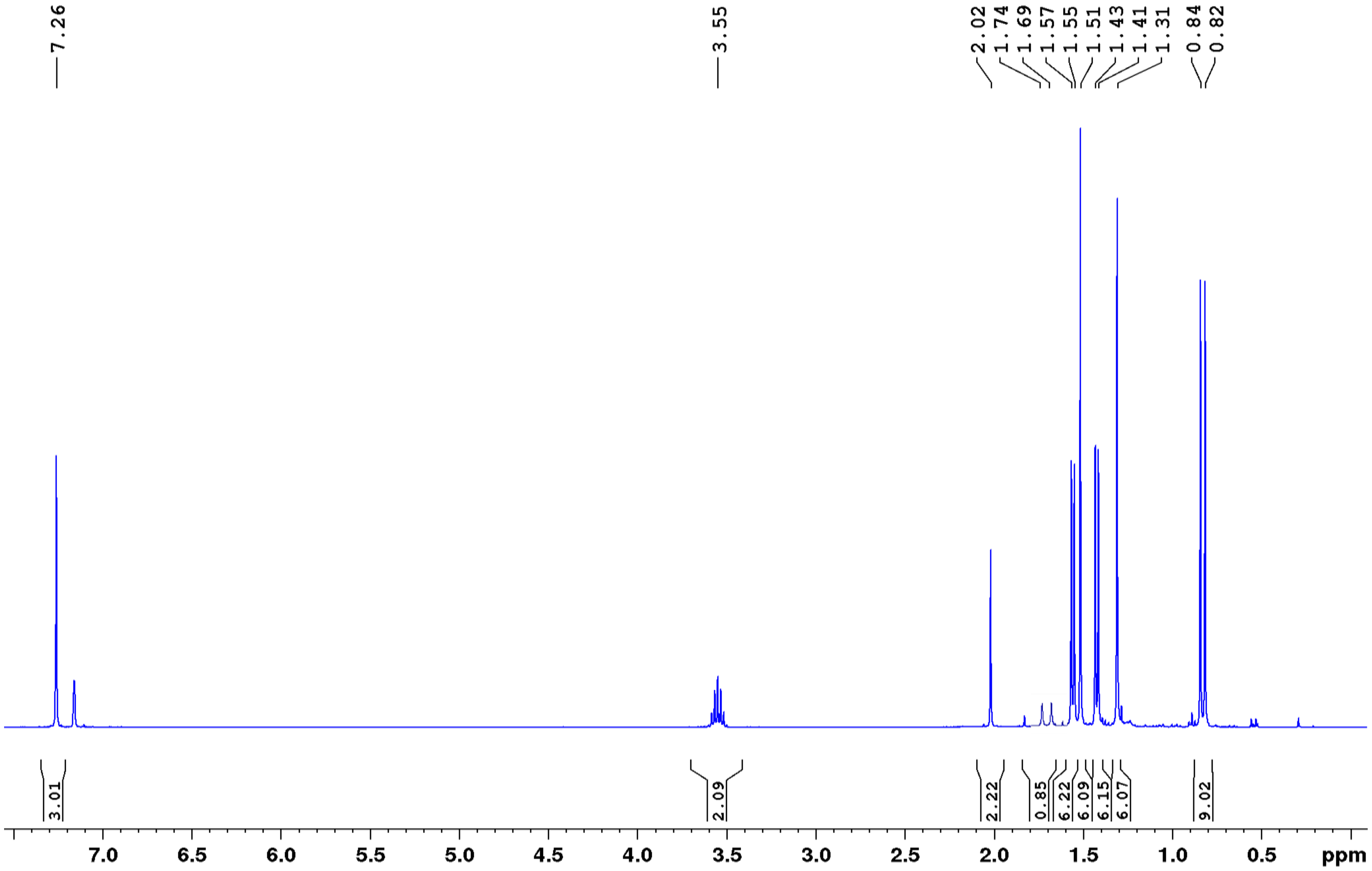

Figure S2. $^{13}C\{^{1}H\}$ NMR spectrum of **1** in C_6D_6.

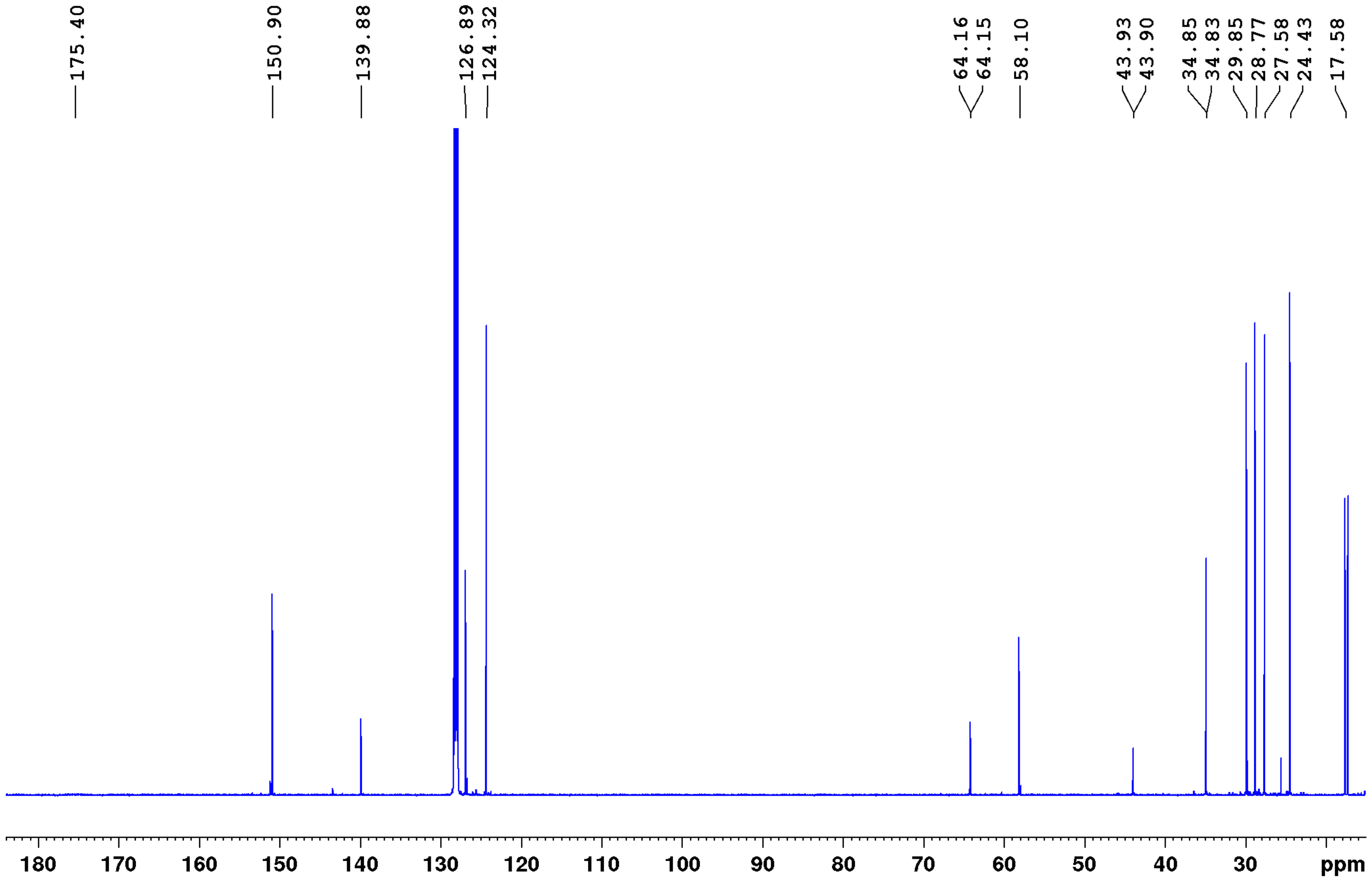

Figure S3. ^{11}B NMR spectrum of **1** in C_6D_6.

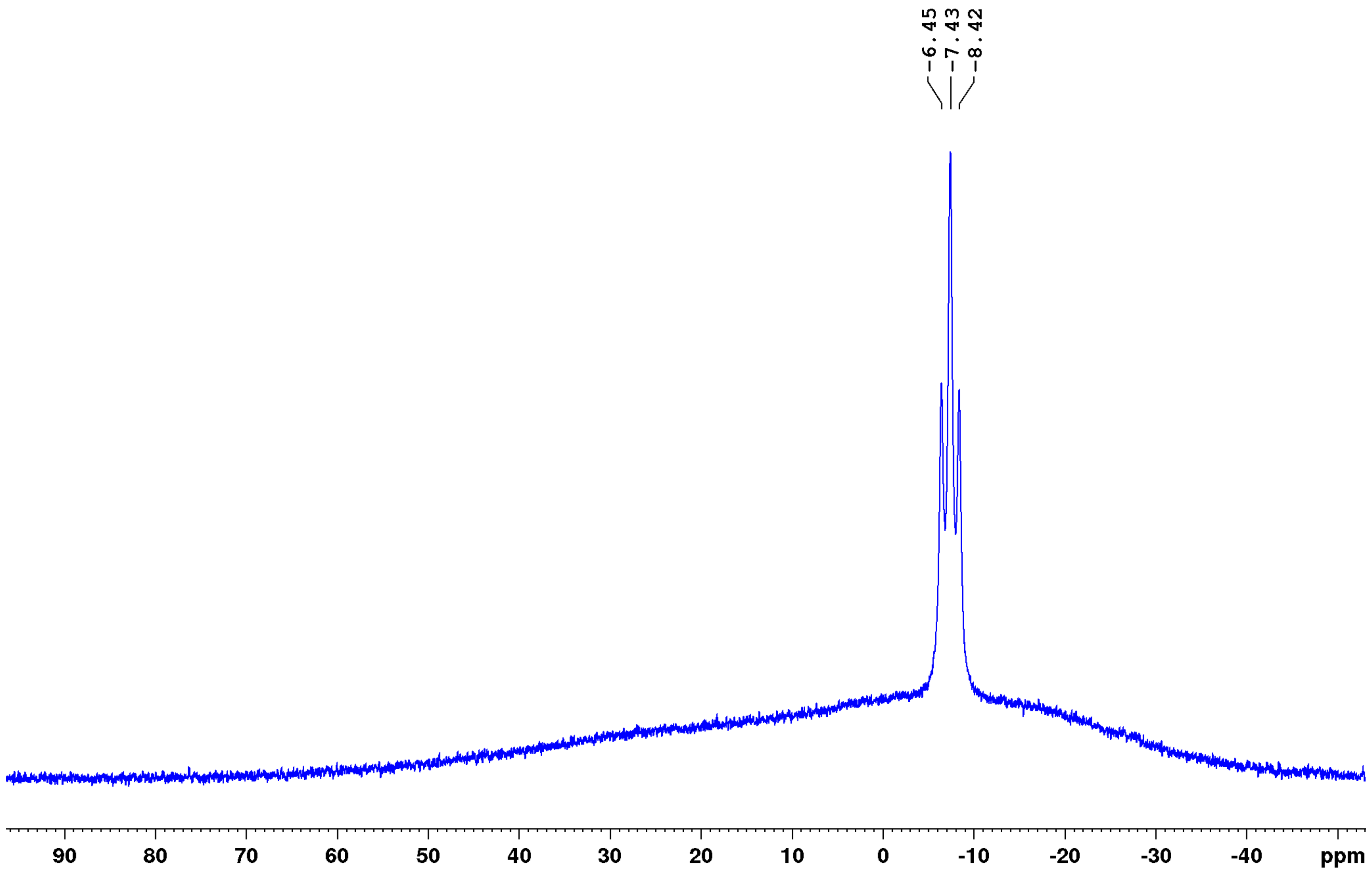

Figure S4. $^{31}P\{^{1}H\}$ NMR spectrum of **1** in C_6D_6.

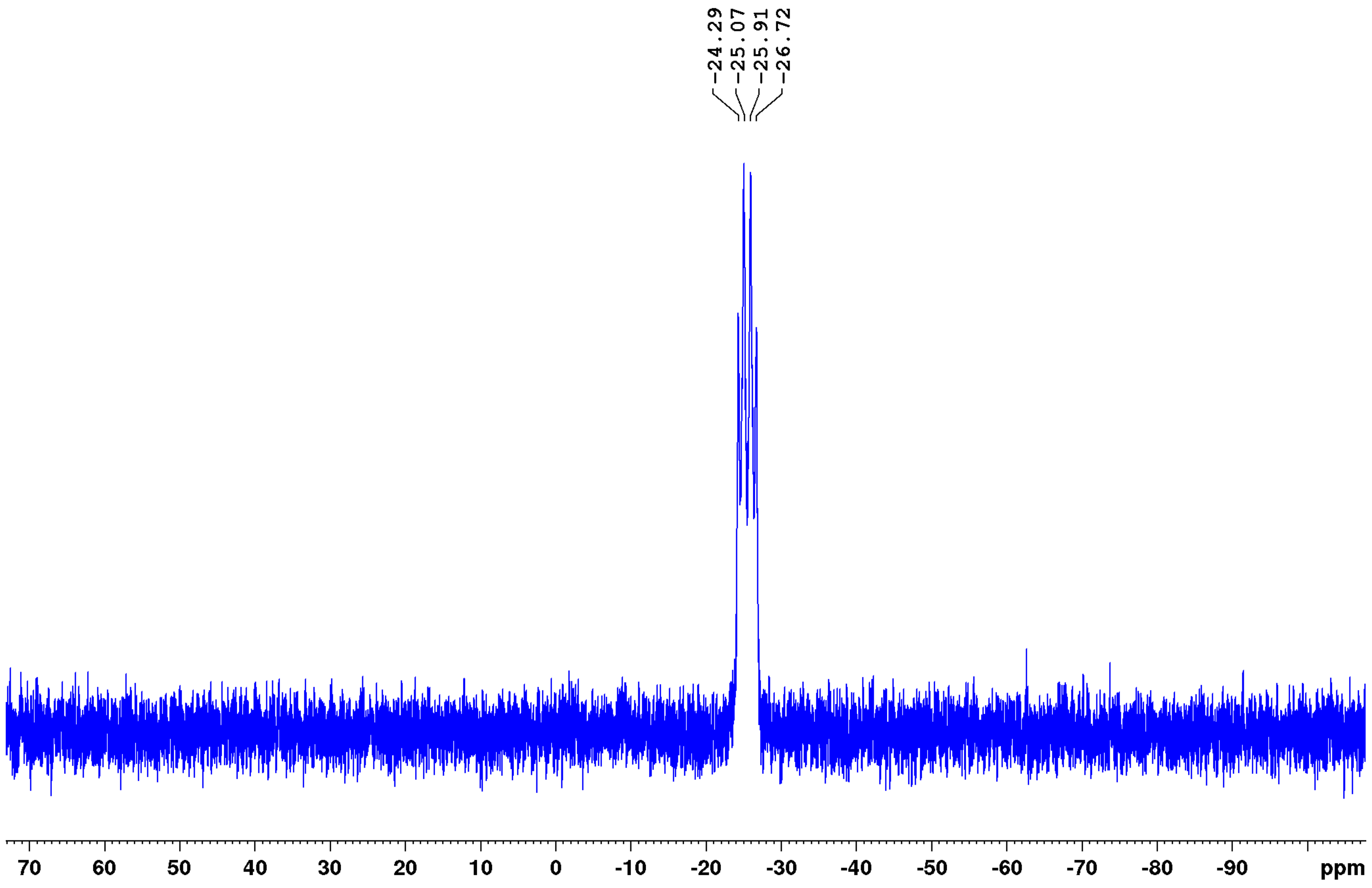

Figure S5. $^{1}H\{^{11}B\}$ NMR spectrum of **1-BCl₃** in $CDCl_3$. The additional resonances at 0.88 (t) and 1.26 (m) ppm belong to residual hexane from crystallization.

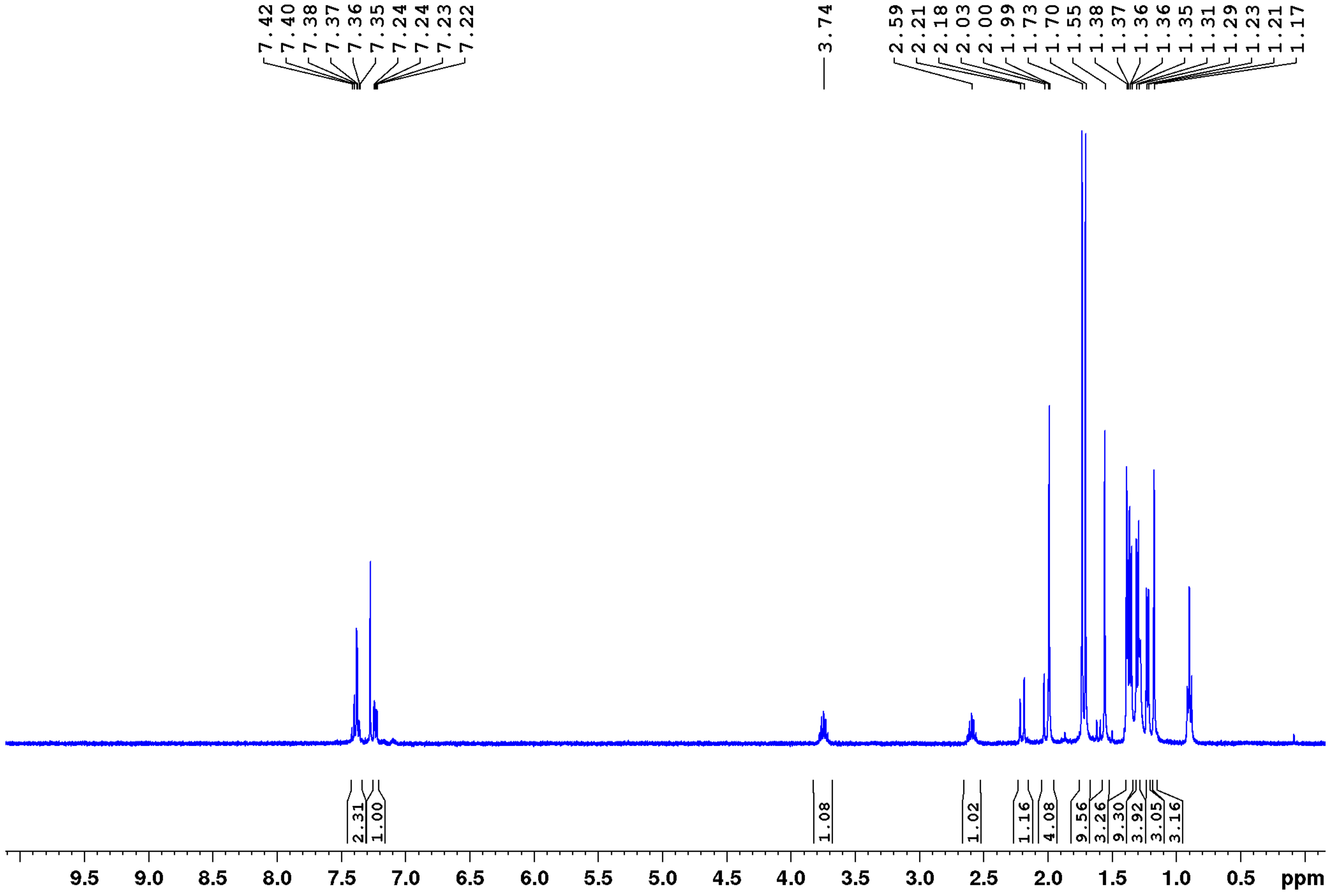

Figure S6. $^{13}C\{^1H\}$ NMR spectrum of **1-BCl₃** in $CDCl_3$. The additional resonances at 31.8, 22.8 and 14.3 ppm belong to residual hexane from crystallization.

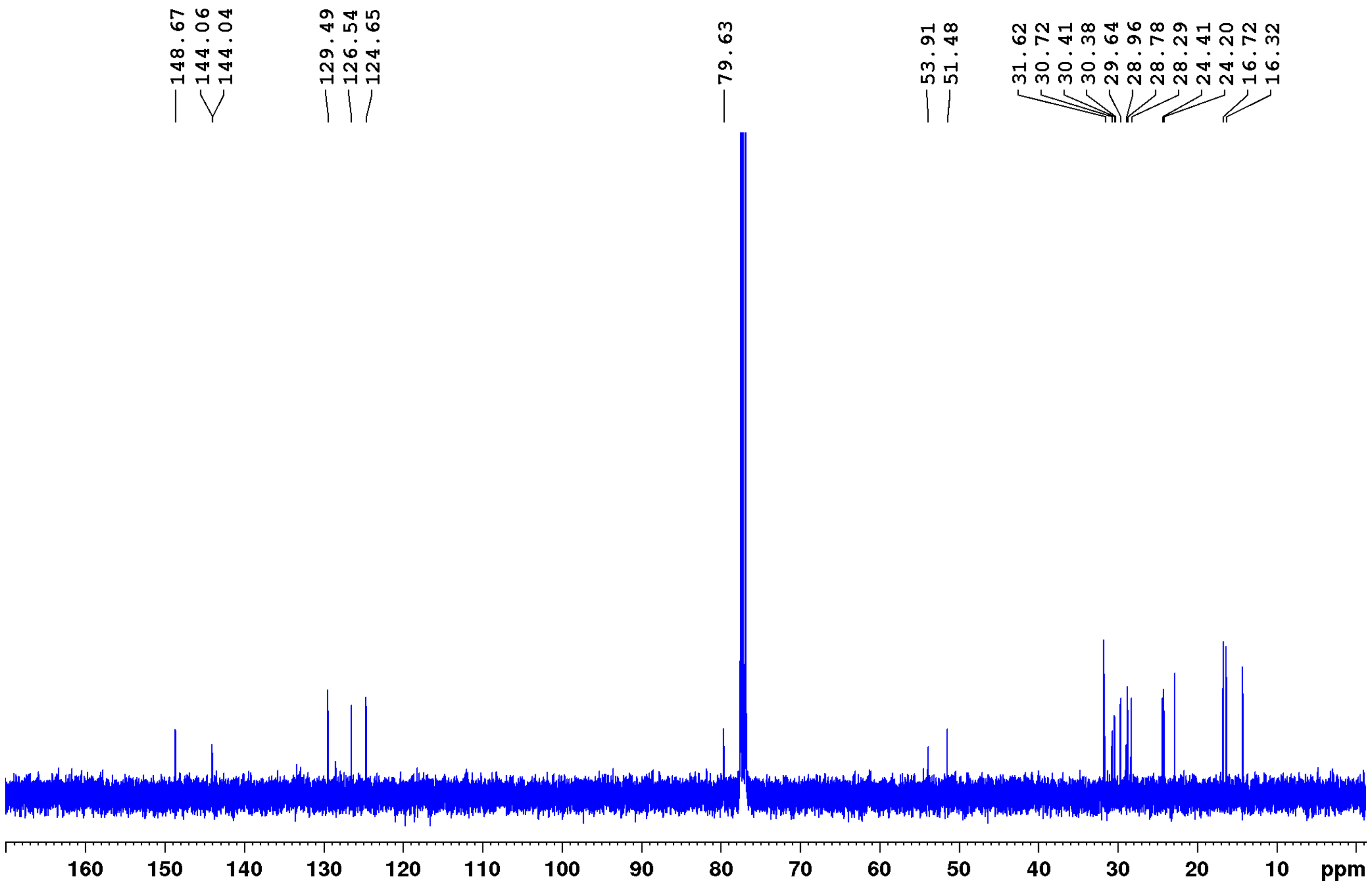

Figure S7. ^{11}B NMR spectrum of **1-BCl₃** in CDCl₃.

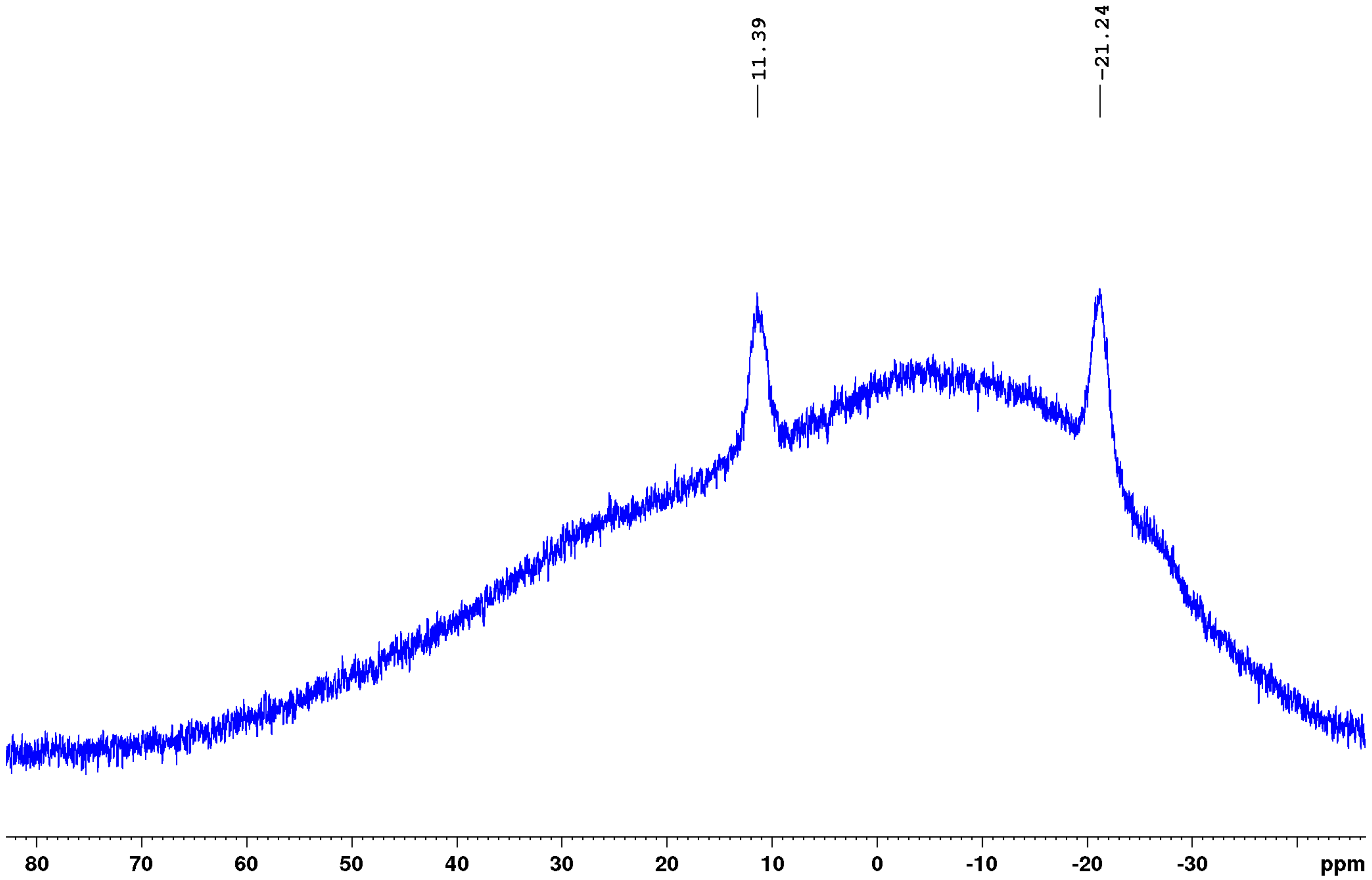

Figure S8. $^{31}P\{^{1}H\}$ NMR spectrum of **1-BCl₃** in $CDCl_3$.

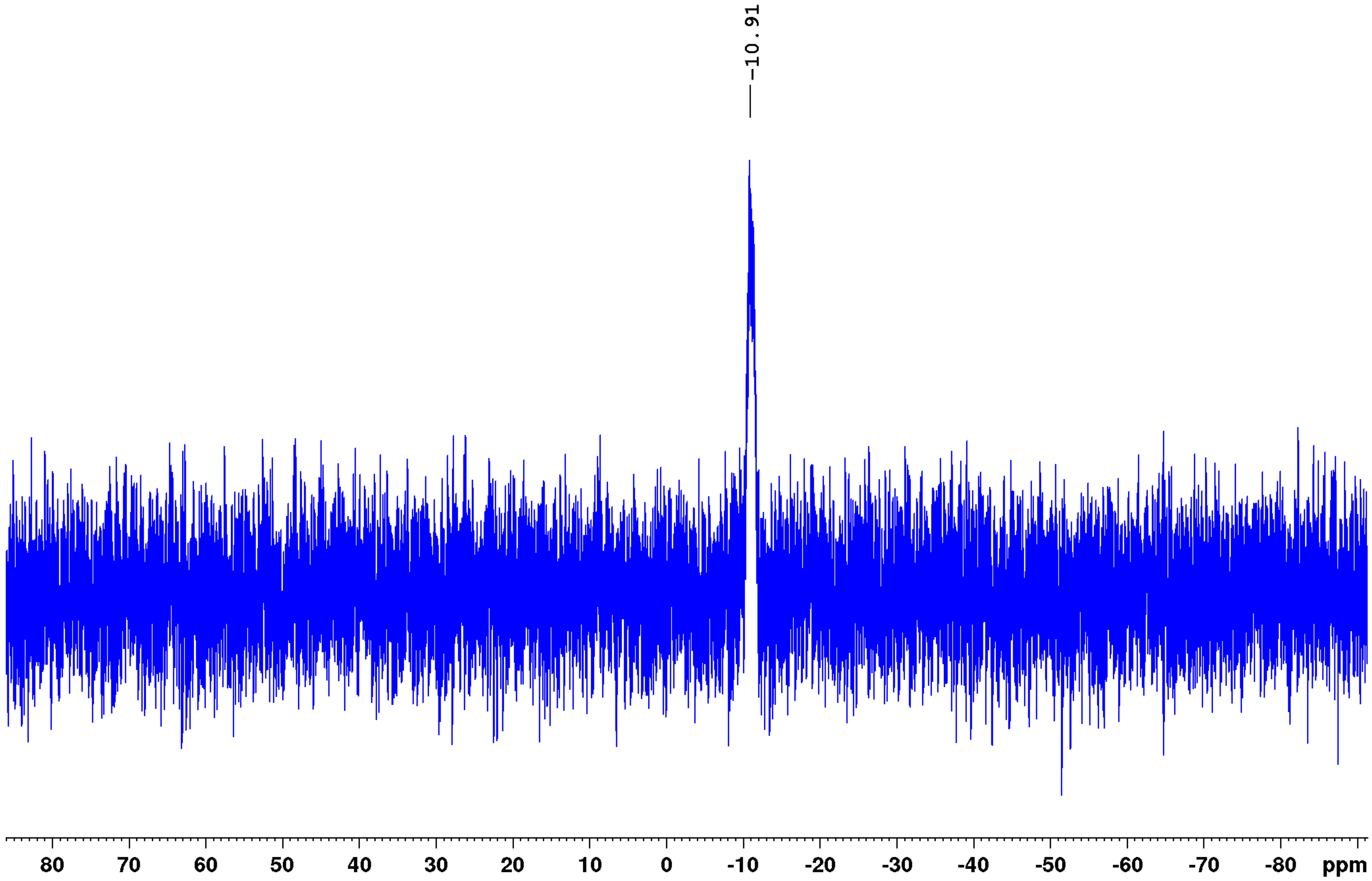

Figure S9. $^{1}H\{^{11}B\}$ NMR spectrum of **1-AlCl₃** in $CDCl_3$. The additional resonances at 0.88 (t) and 1.26 (m) ppm belong to residual hexane from crystallization.

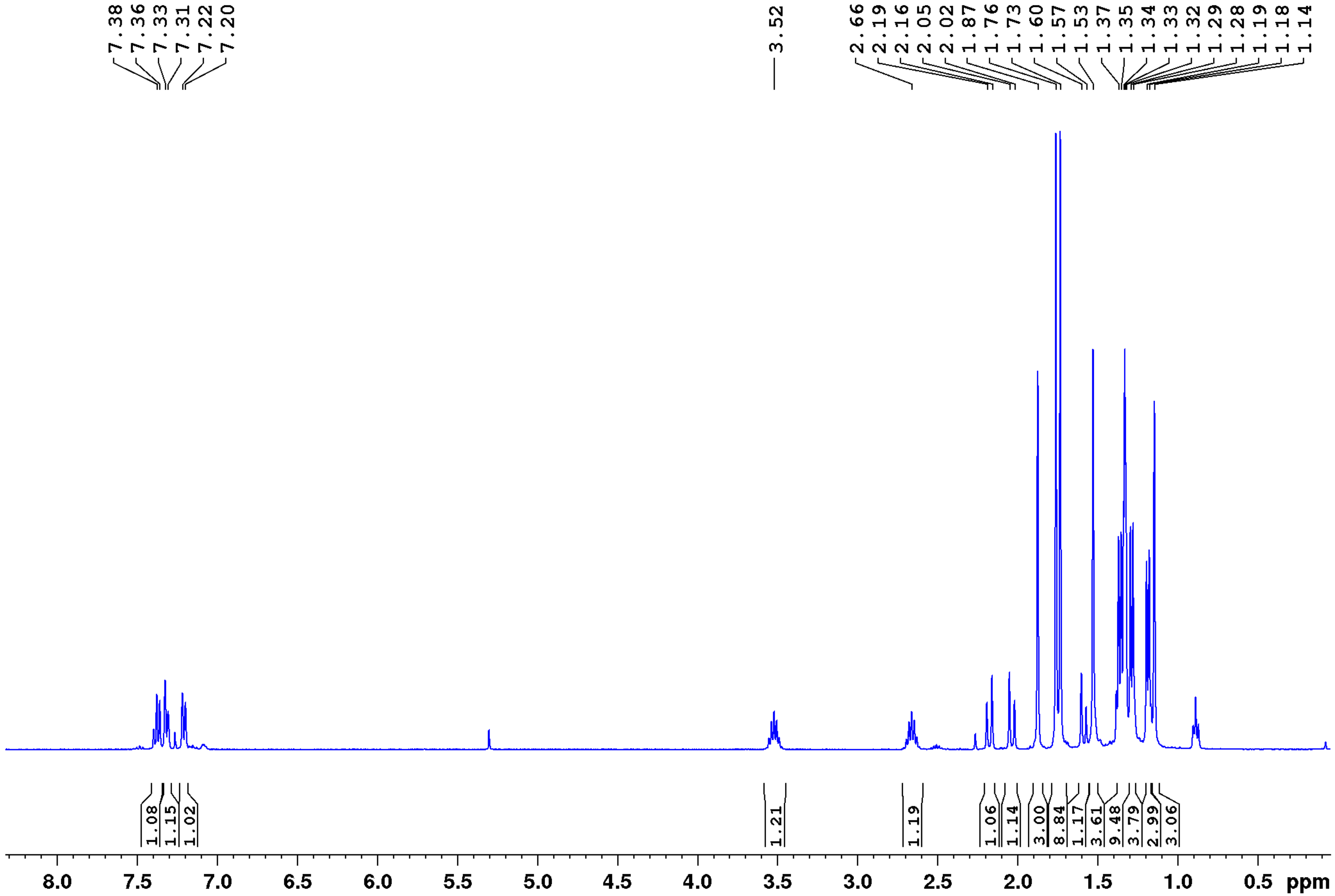

Figure S10. $^{13}C\{^{1}H\}$ NMR spectrum of **1-AlCl₃** in $CDCl_3$. The additional resonances at 31.8, 22.8 and 14.3 ppm belong to residual hexane from crystallization.

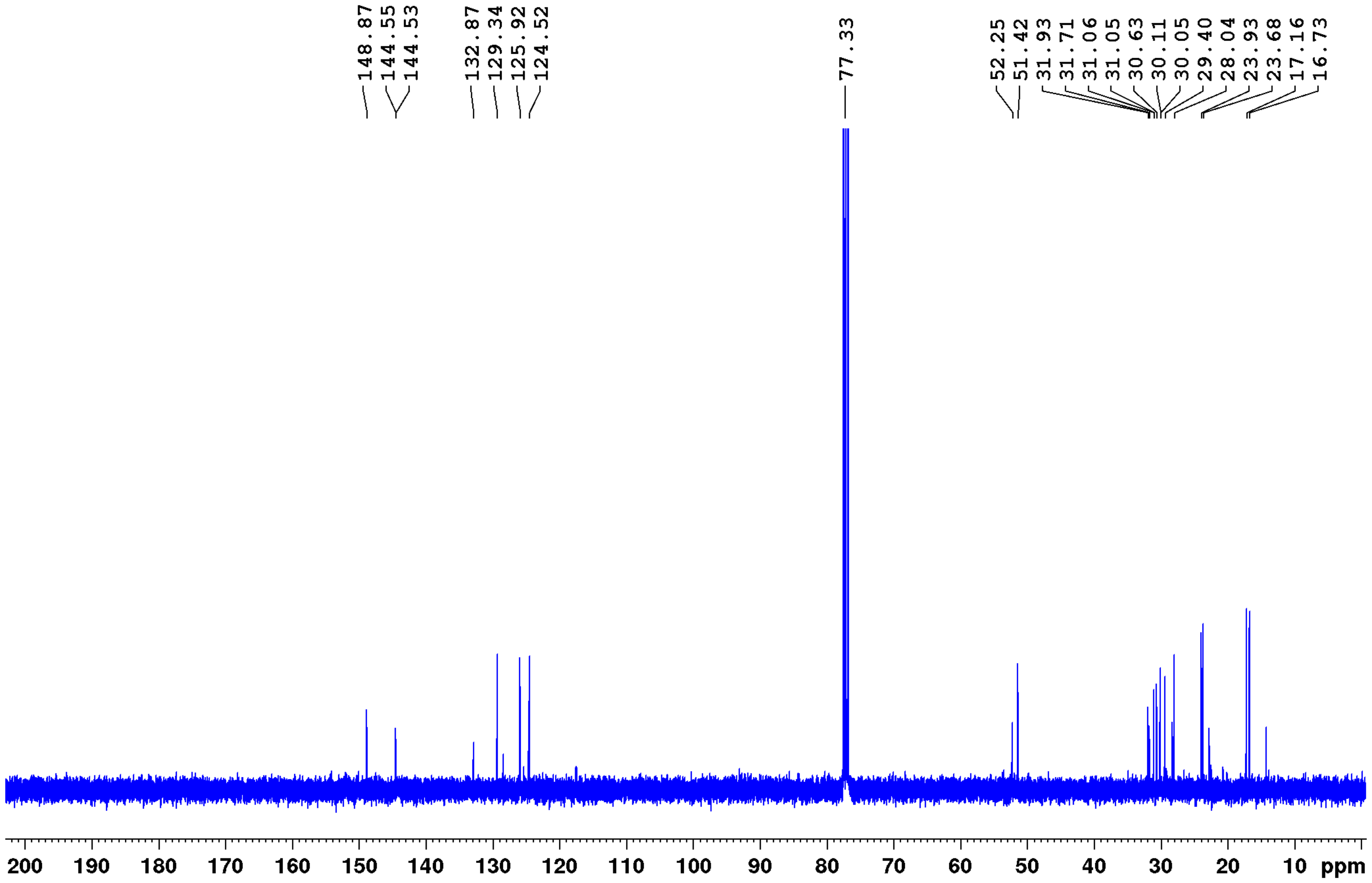

Figure S11. ^{11}B NMR spectrum of **1-AlCl₃** in $CDCl_3$.

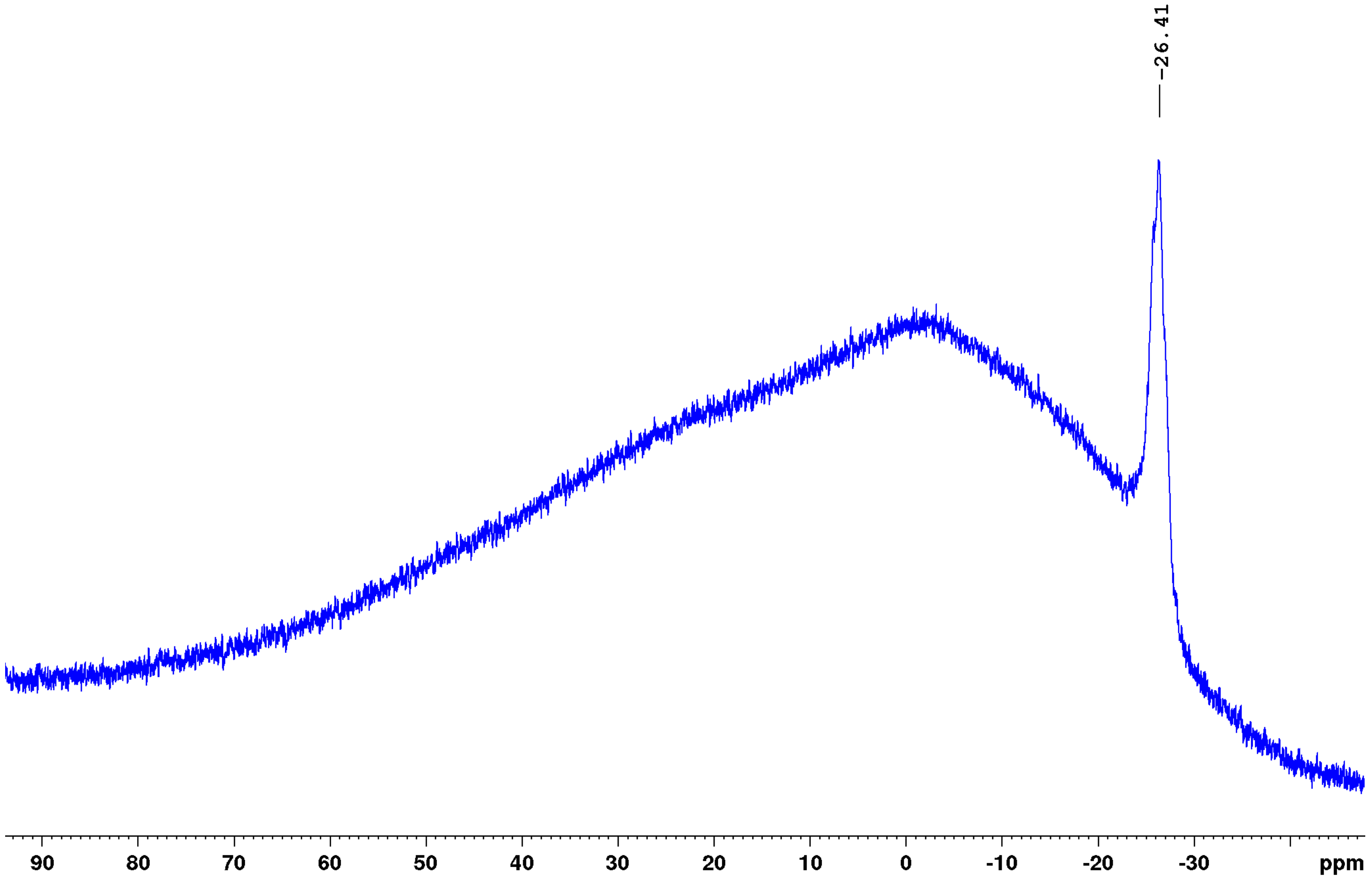

Figure S12. $^{31}P\{^1H\}$ NMR spectrum of **1-AlCl₃** in $CDCl_3$.

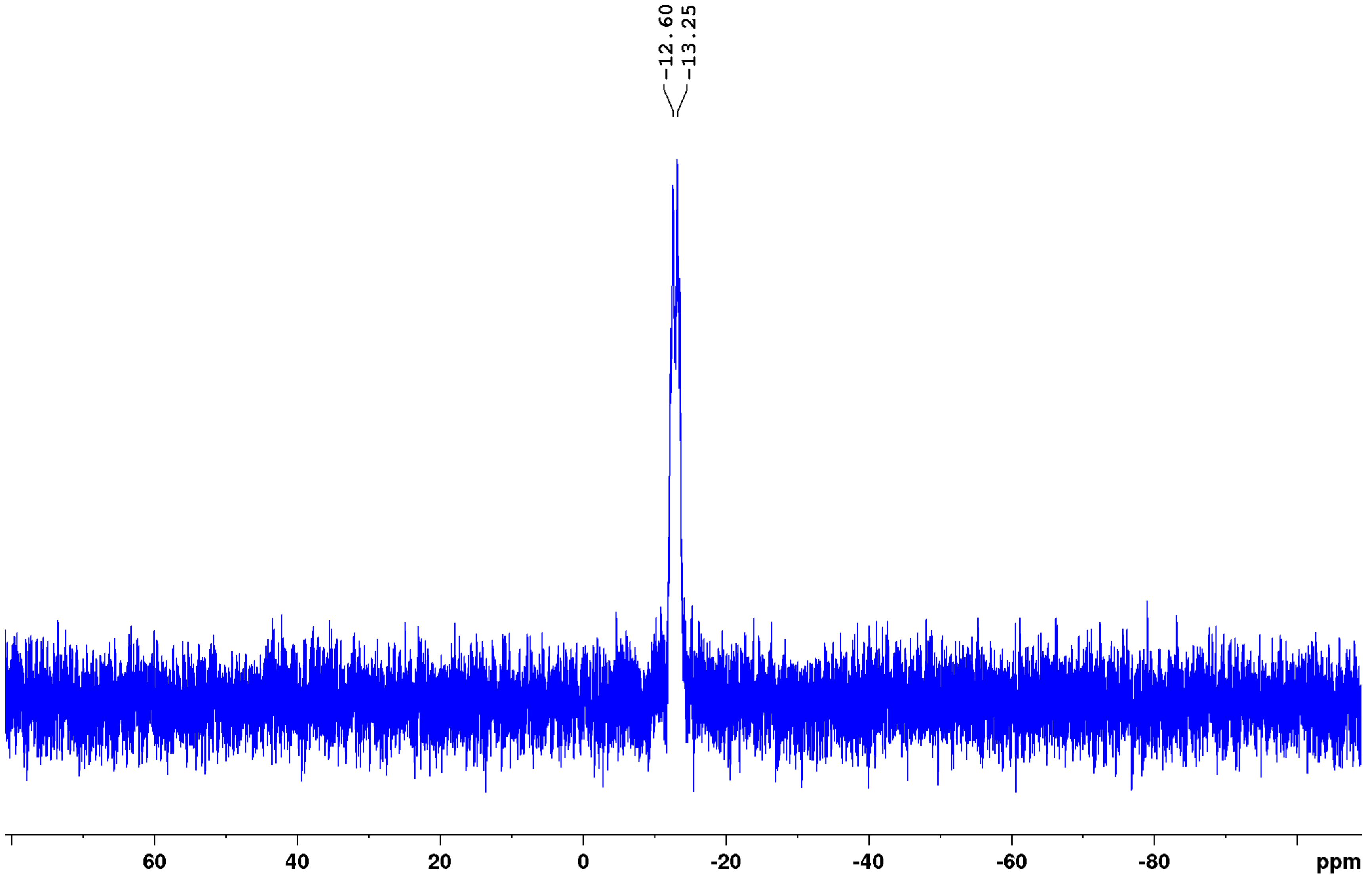

Figure S13. ^{27}Al NMR spectrum of **1-AlCl$_3$** in $CDCl_3$.

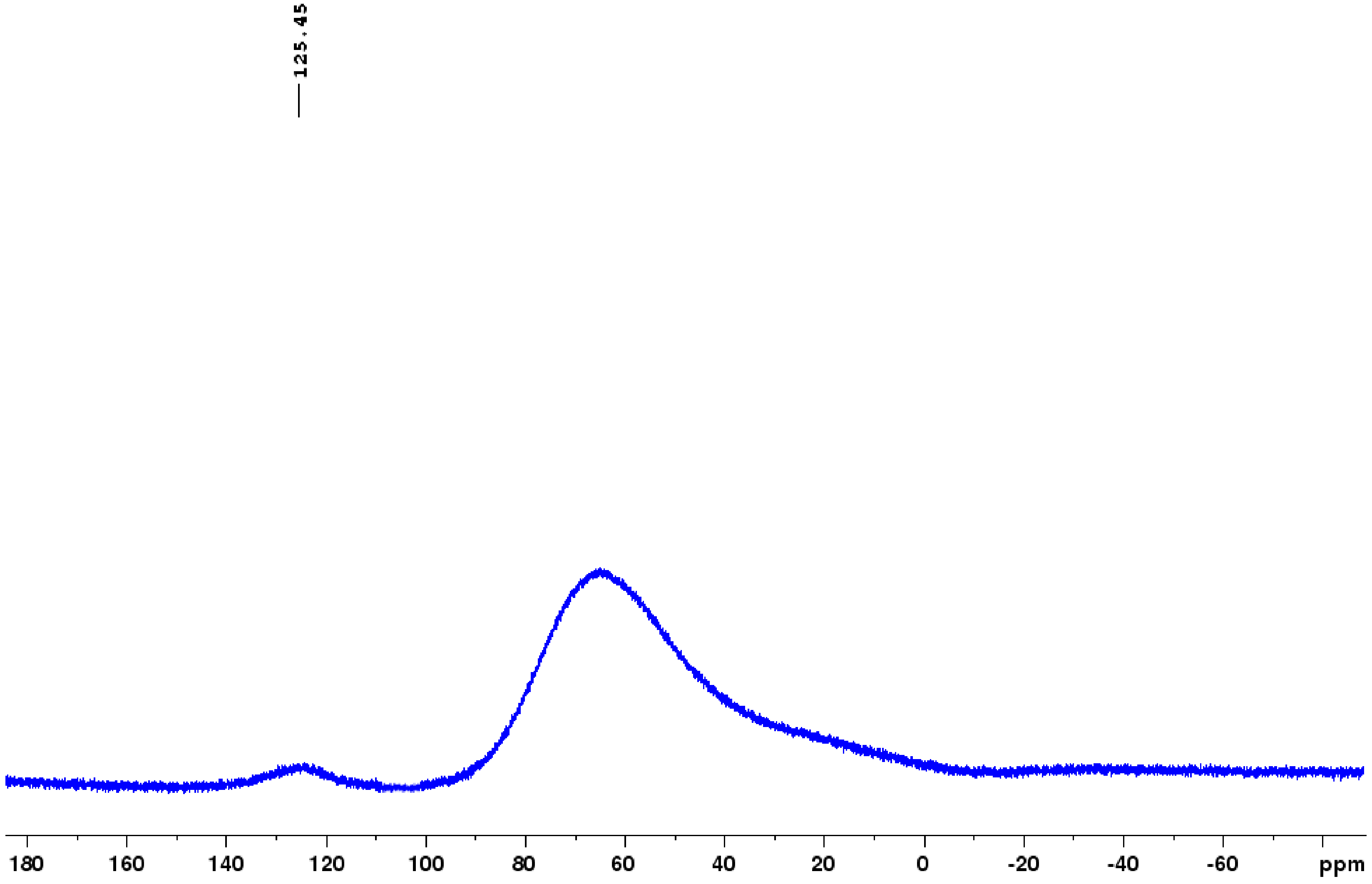

Figure S14. $^{1}H\{^{11}B\}$ NMR spectrum of **1-GaCl₃** in $CDCl_3$. The additional resonances at 0.88 (t) and 1.26 (m) ppm belong to residual hexane from crystallization.

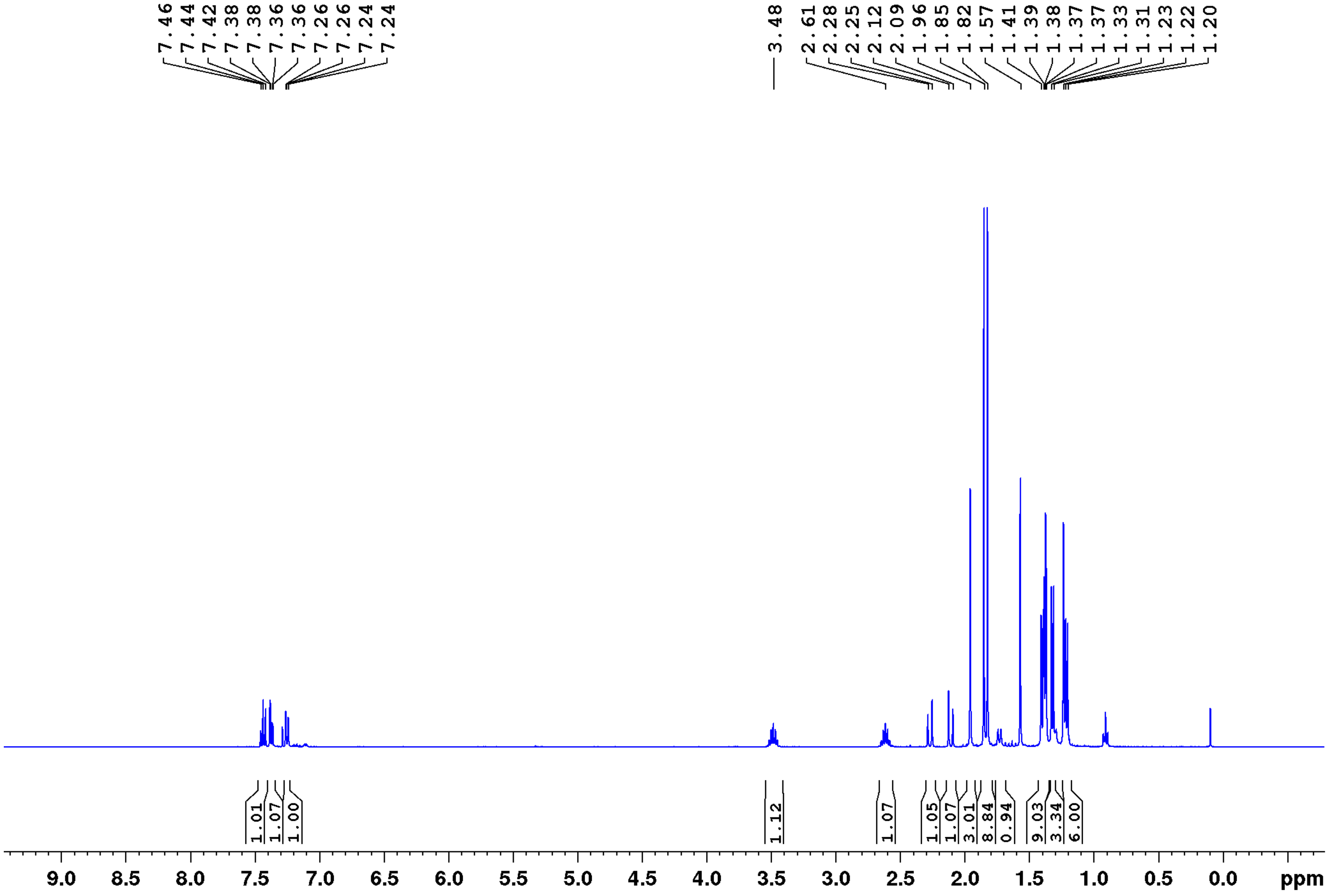

Figure S15. $^{13}C\{^1H\}$ NMR spectrum of **1-GaCl₃** in $CDCl_3$. The additional resonances at 31.8, 22.8 and 14.3 ppm belong to residual hexane from crystallization.

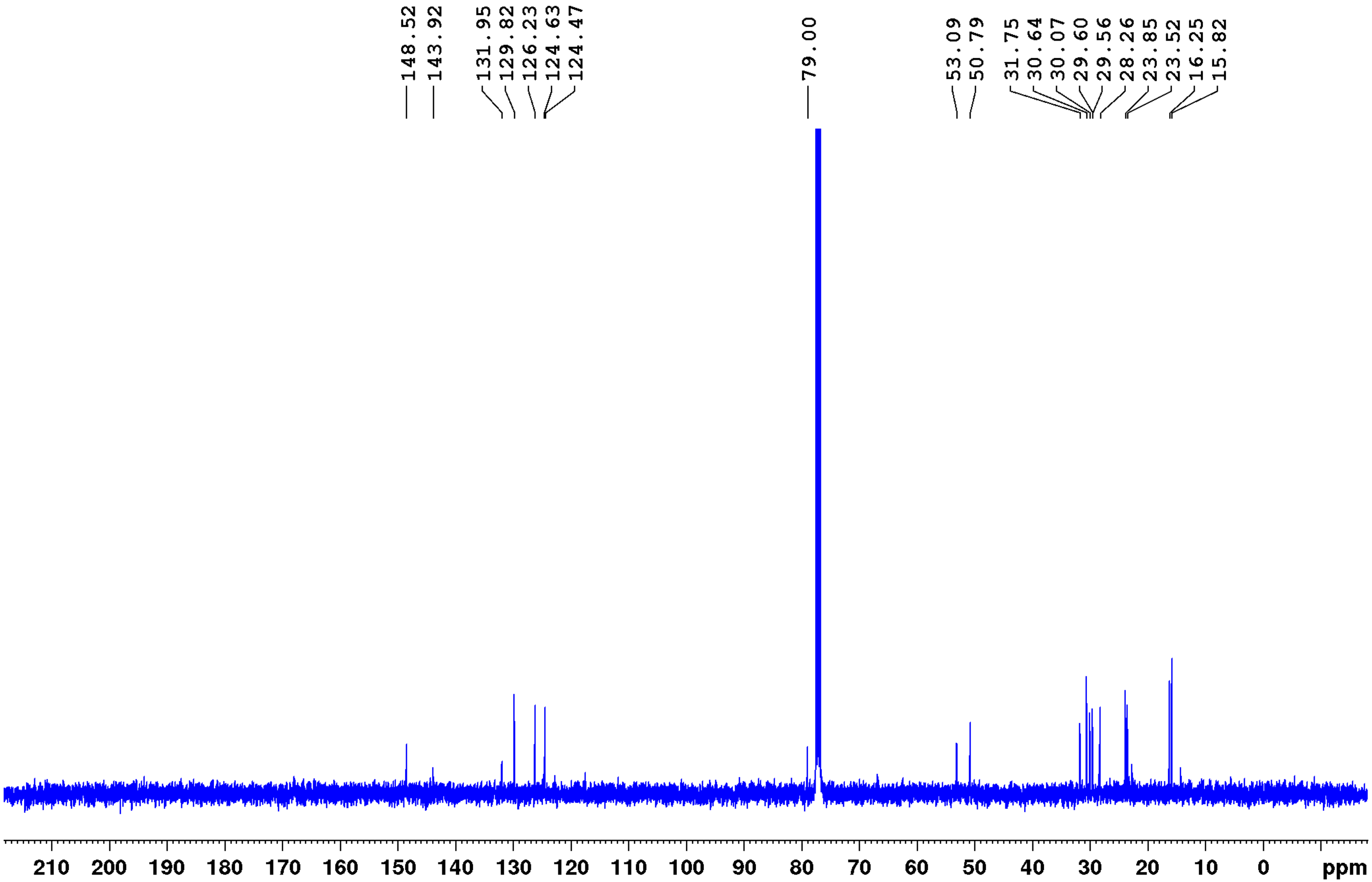

Figure S16. ^{11}B NMR spectrum of **1-GaCl₃** in $CDCl_3$.

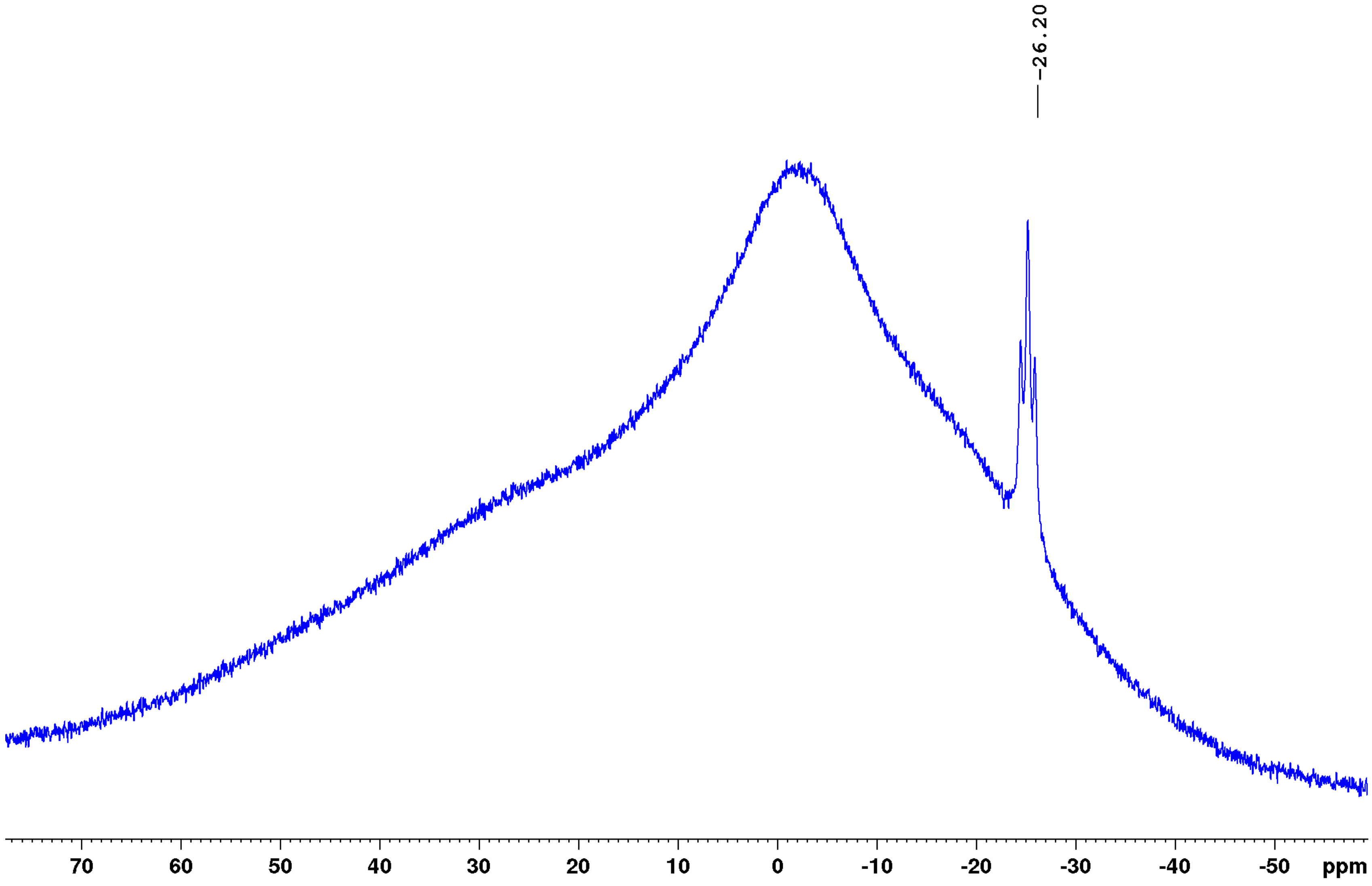

Figure S17. $^{31}P\{^{1}H\}$ NMR spectrum of **1-GaCl₃** in $CDCl_3$.

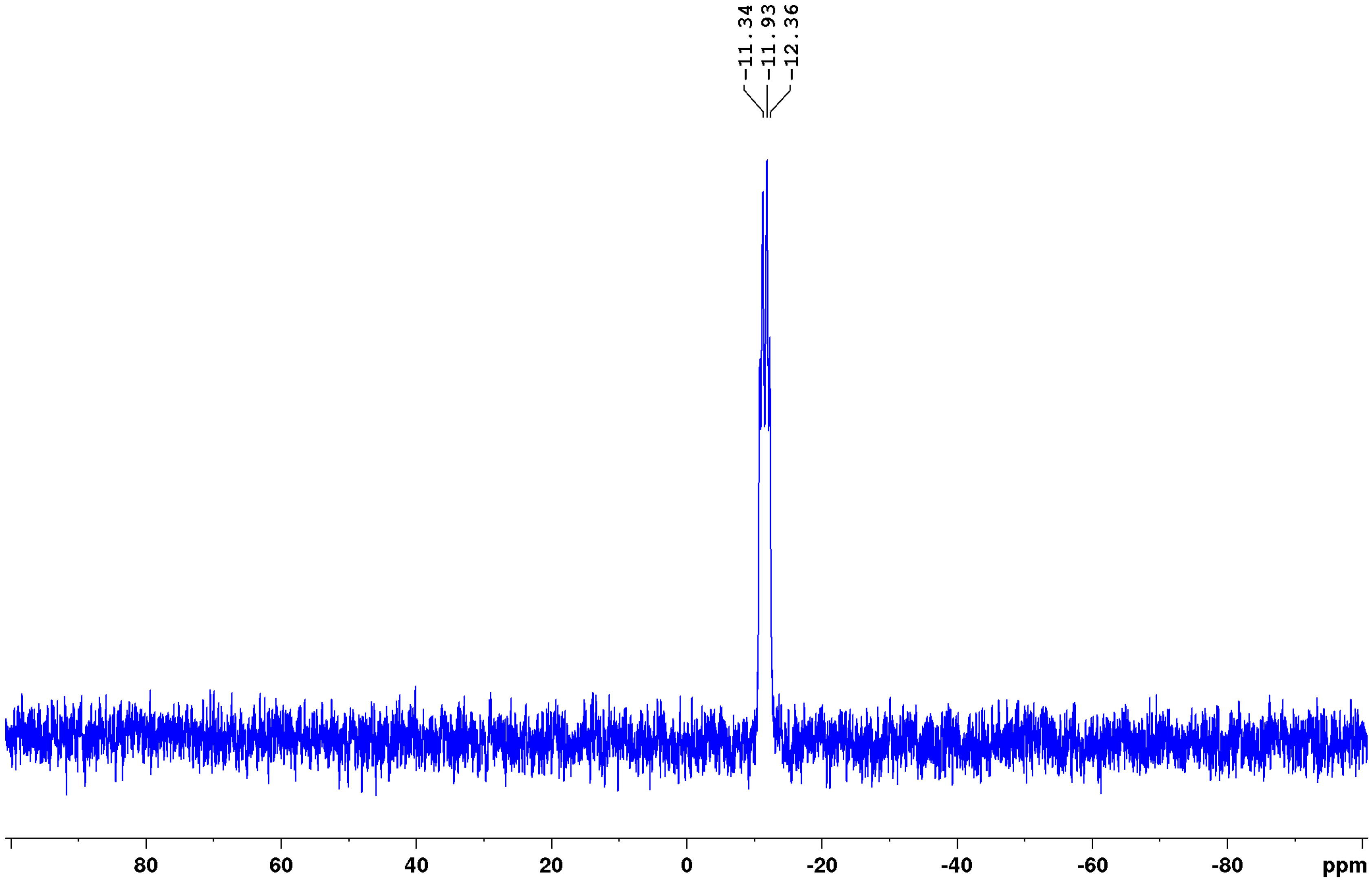

Figure S18. $^{1}H\{^{11}B\}$ NMR spectrum of **1-InCl₃** in $CDCl_3$. The additional resonances at 7.08–7.22 (m) and 0.91 (t) ppm correspond to residual *o*-difluorobenzene and hexane, respectively, from the crystallization solvent.

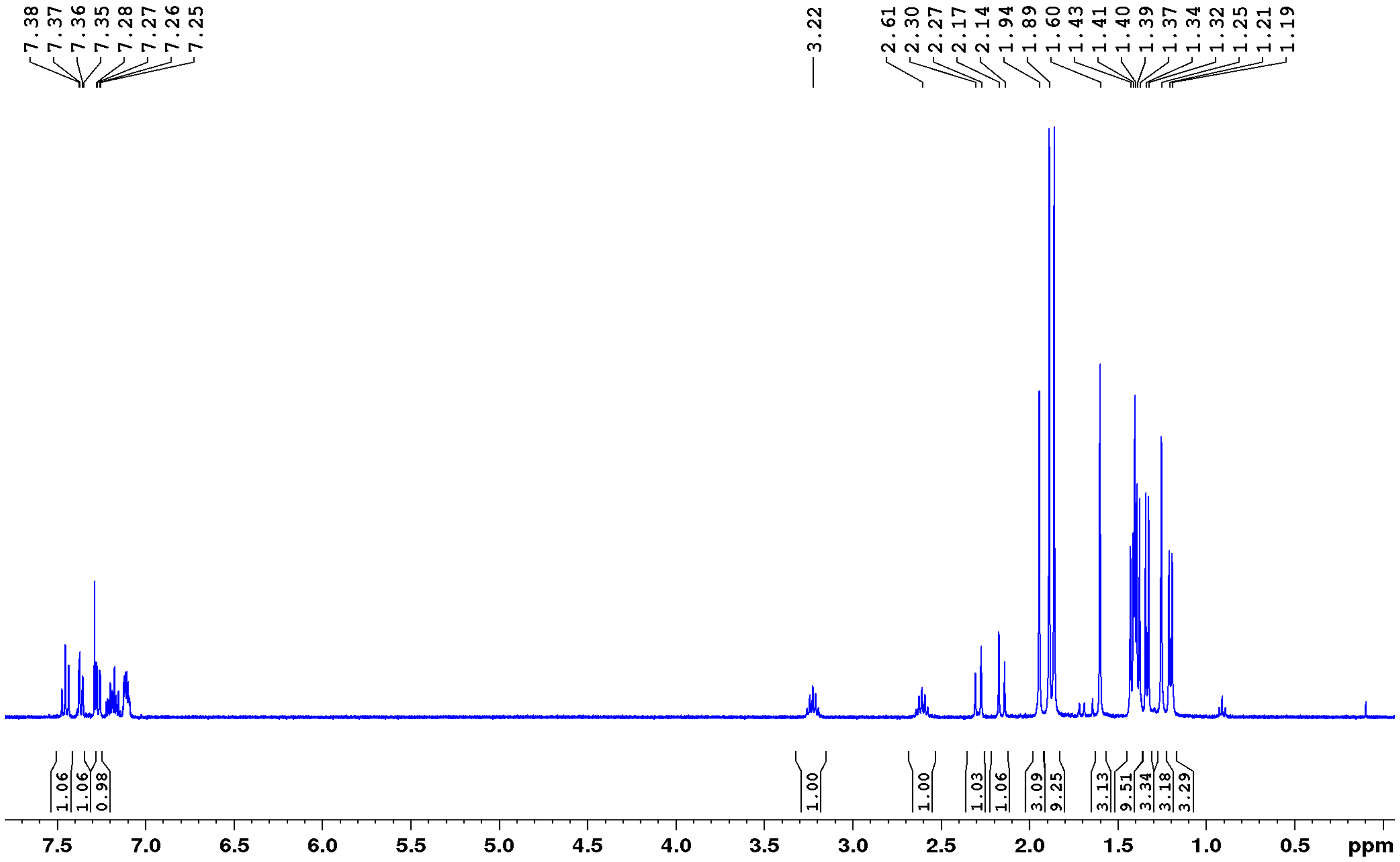

Figure S19. ^{11}B NMR spectrum of **1-InCl$_3$** in $CDCl_3$.

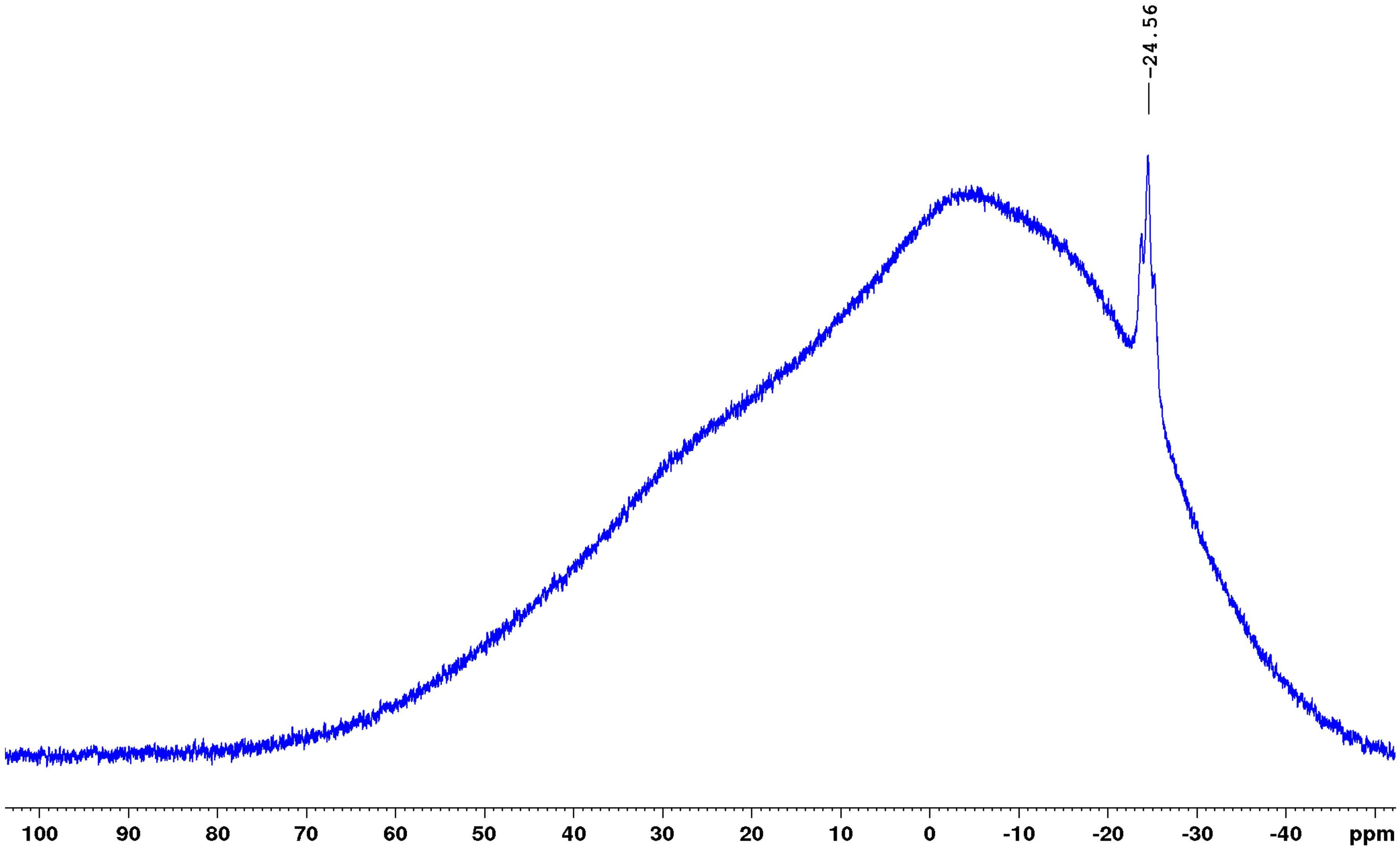

Figure S20. $^{31}P\{^1H\}$ NMR spectrum of **1-InCl₃** in $CDCl_3$.

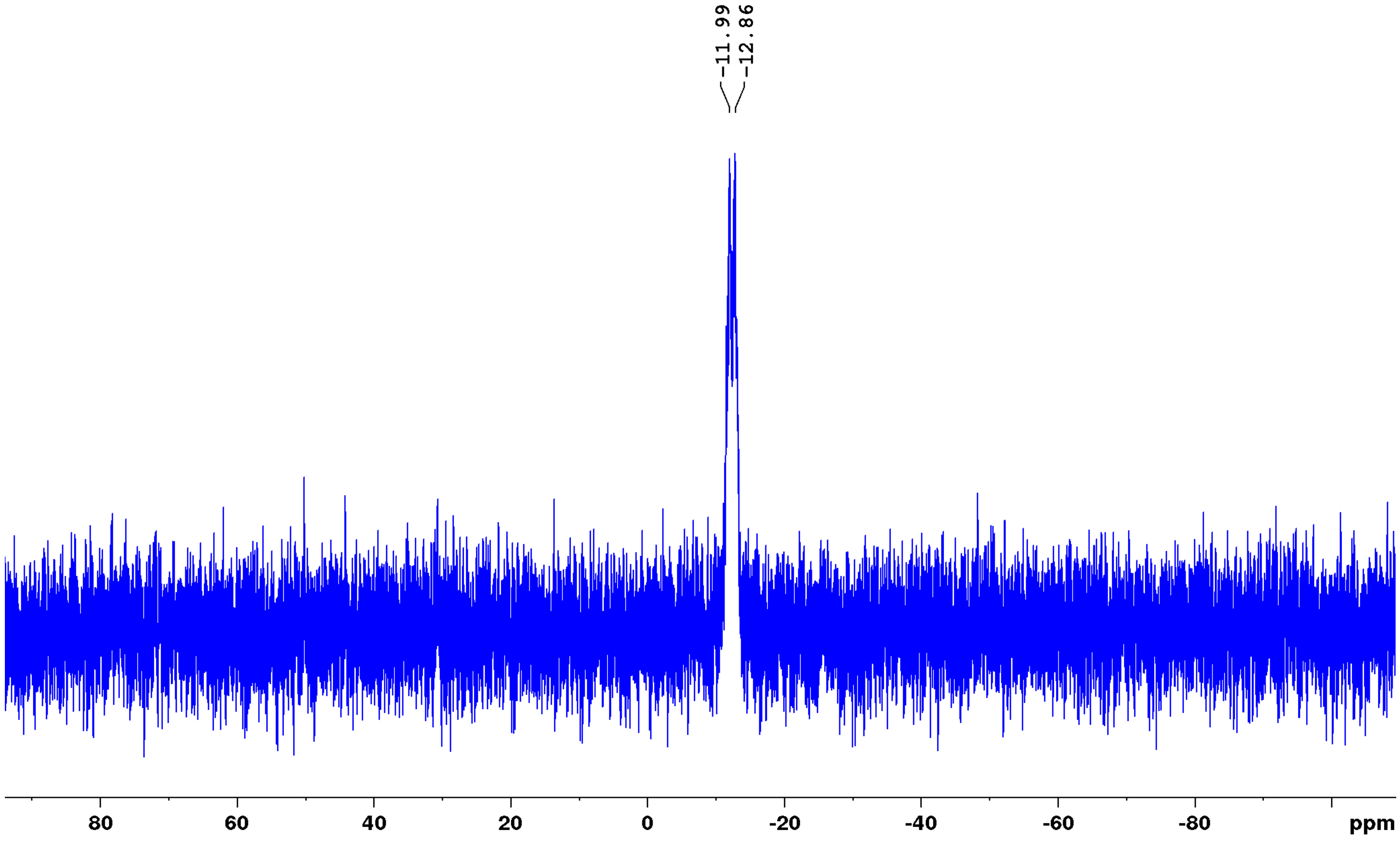

Figure S21. ^{1}H NMR spectrum of **[1-BF_2][BF_4]** in 1:1 PF_2/C_6D_6.

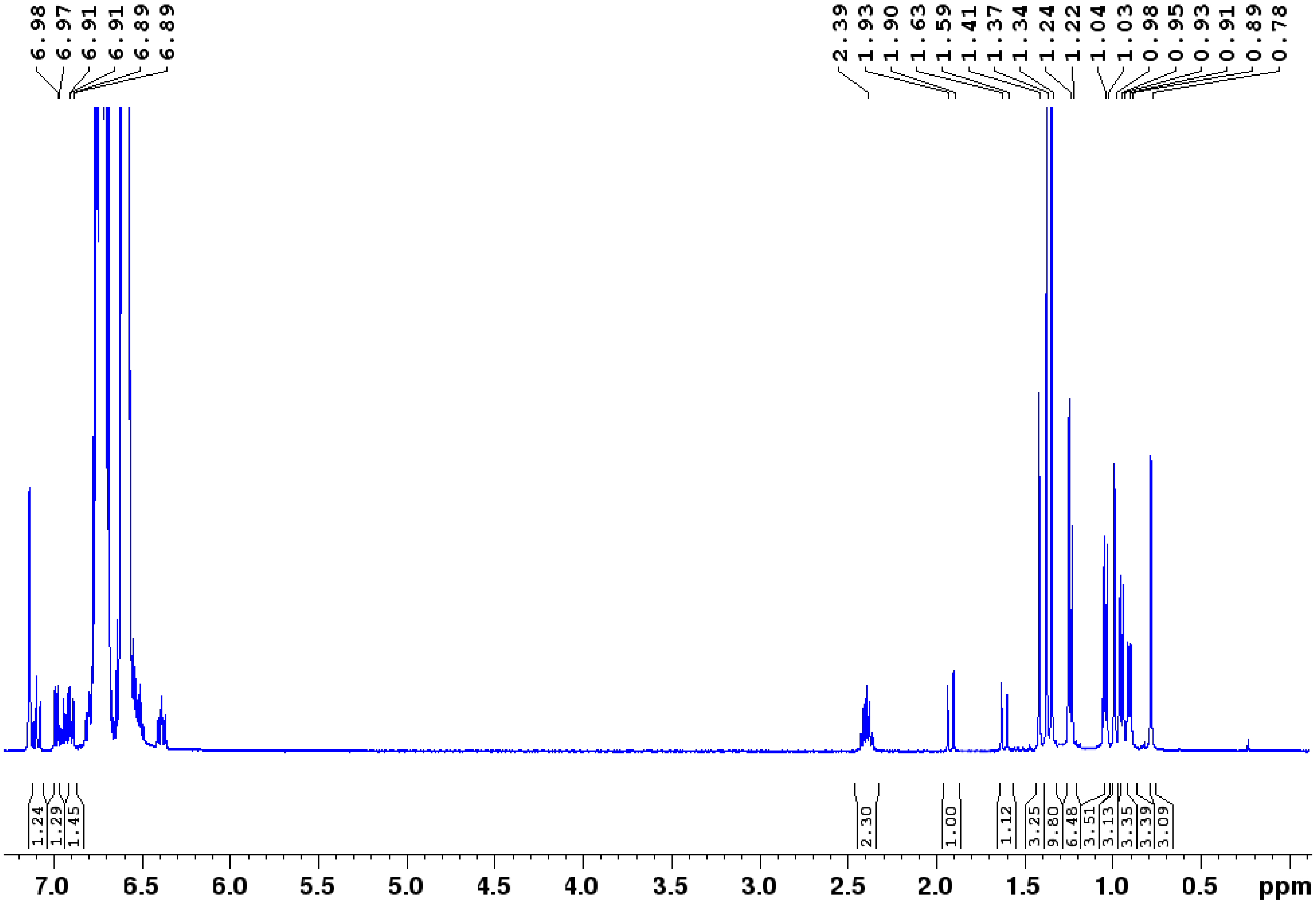

Figure S22. $^{13}C\{^{1}H\}$ NMR spectrum of **[1-BF$_2$][BF$_4$]** in 1:1 PF_2/C_6D_6.

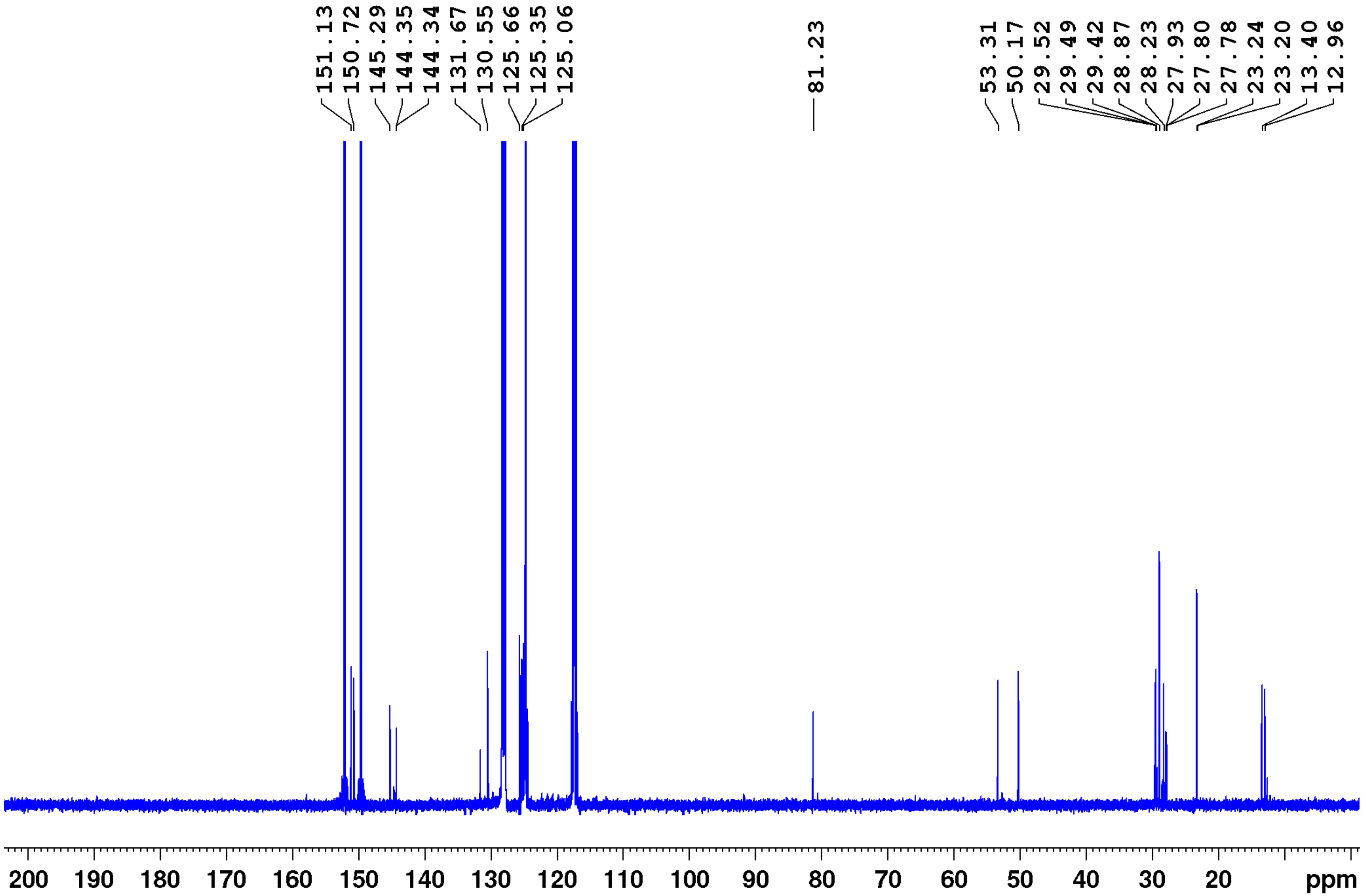

Figure S23. ^{11}B NMR spectrum of **[1-BF$_2$][BF$_4$]** in 1:1 PF$_2$/C$_6$D$_6$.

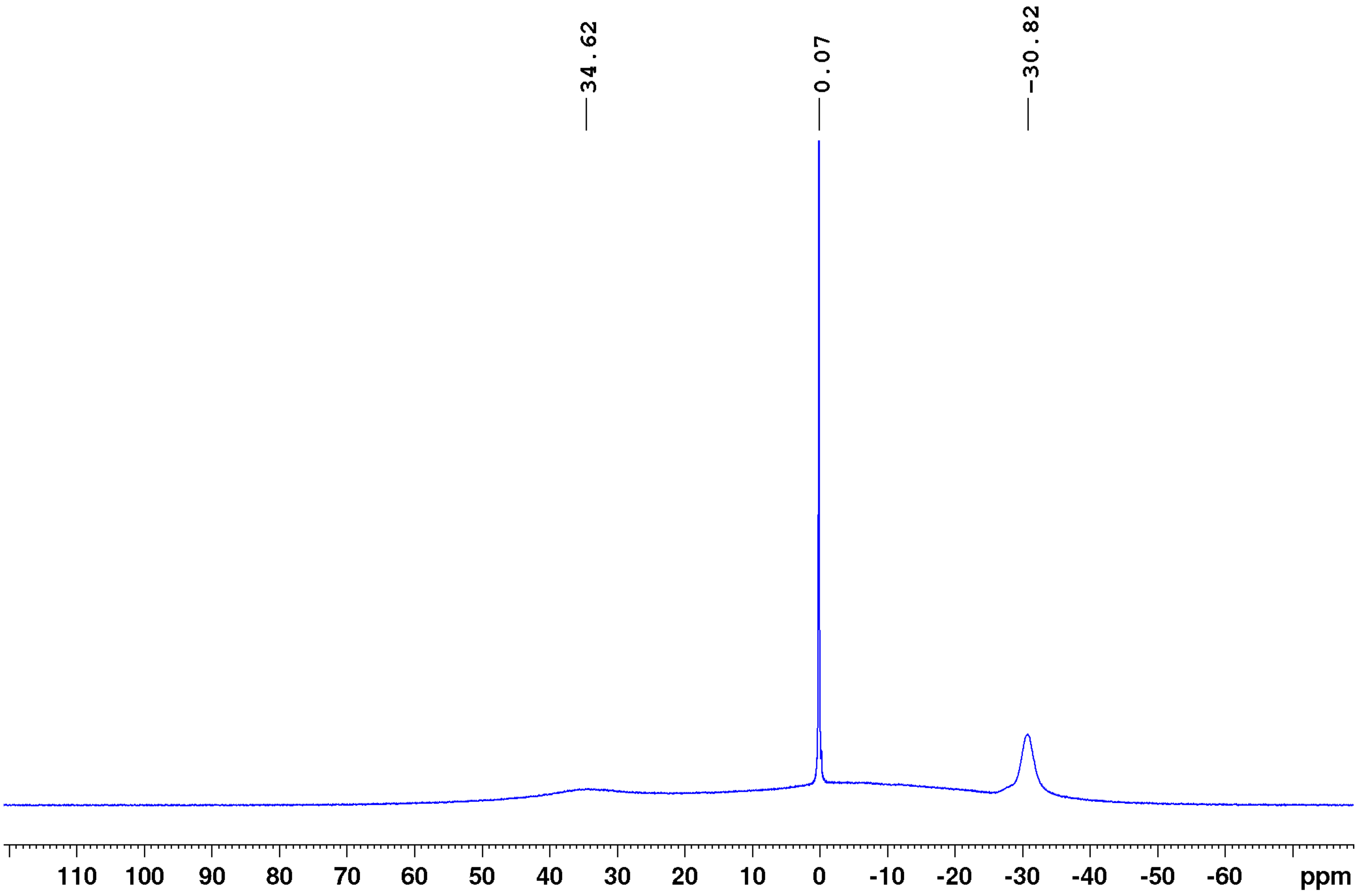

Figure S24. $^{31}P\{^{1}H\}$ NMR spectrum of **[1-BF$_2$][BF$_4$]** in 1:1 PF$_2$/C_6D_6.

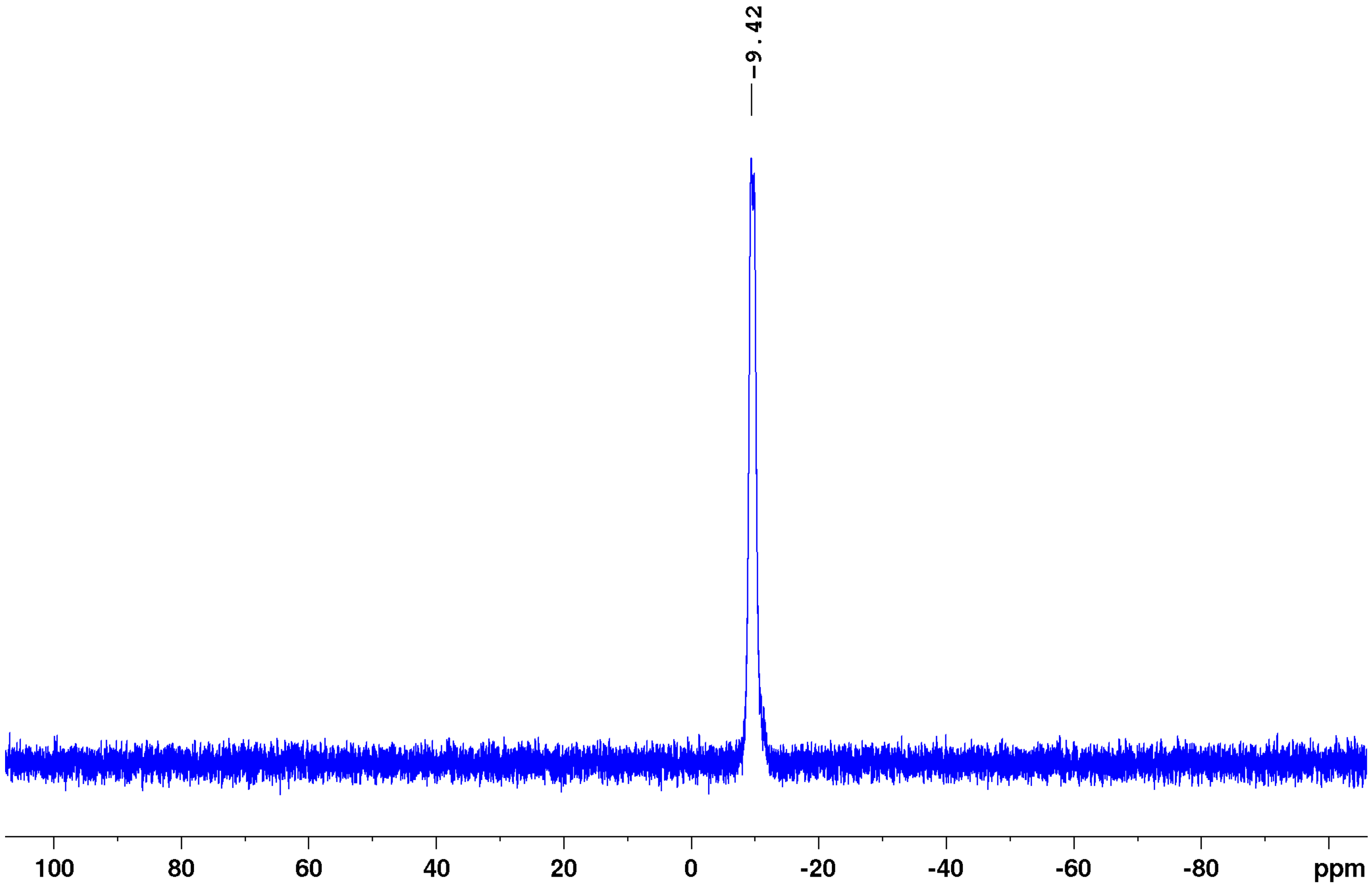

Figure S25. ^{19}F NMR spectrum of **[1-BF$_2$][BF$_4$]** in 1:1 PF_2/C_6D_6. The multiplet at –138 ppm correspond to the DFB solvent.

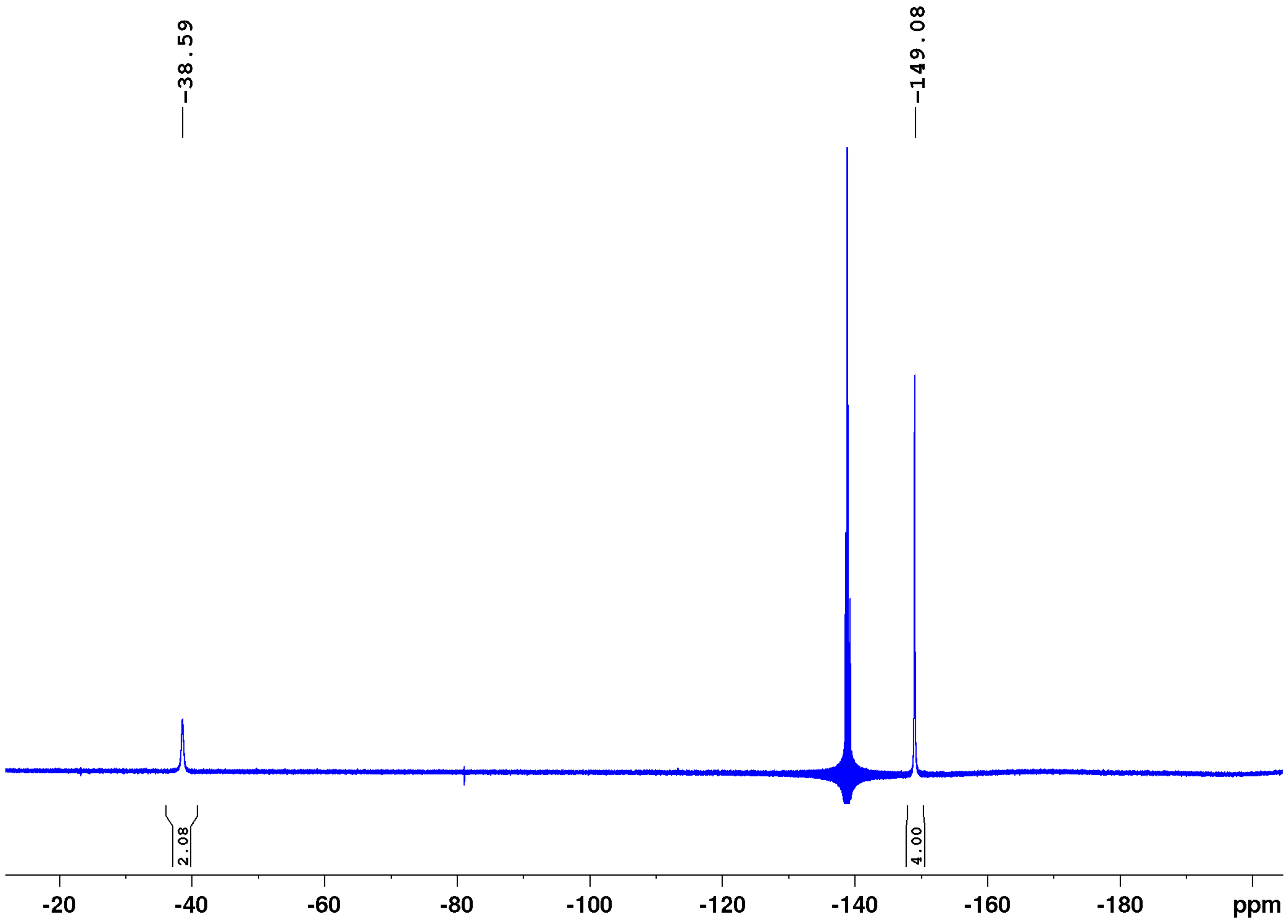

Figure S26. $^{1}H\{^{11}B\}$ NMR spectrum of **[1-BCl$_2$][BCl$_4$]** in 1:10 DFB/C_6D_6. The additional resonances at 0.88 (t) and 1.26 (m) ppm belong to residual pentane from washing.

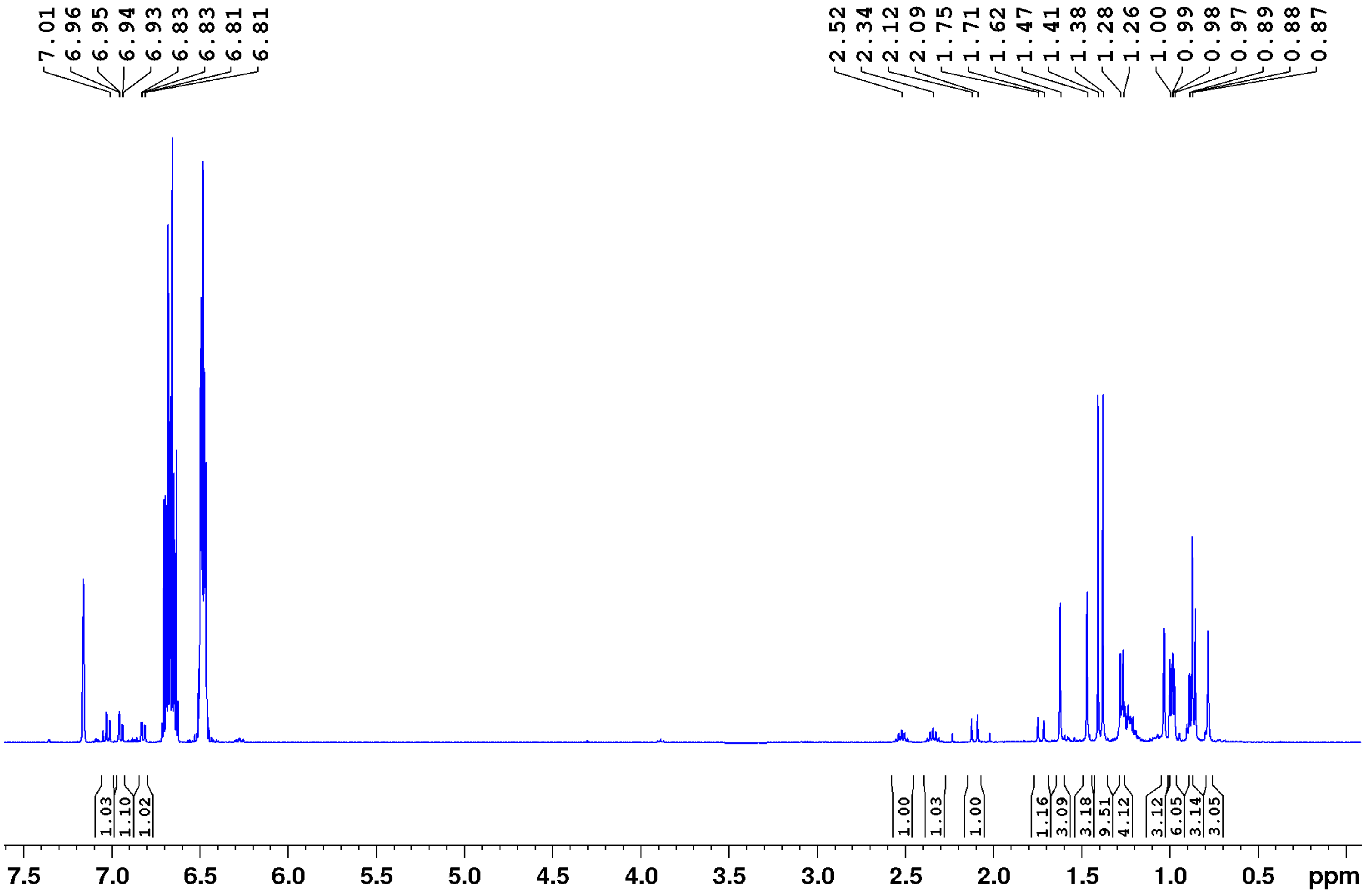

Figure S27. ^{11}B NMR spectrum of **[1-BCl$_2$][BCl$_4$]** in 1:10 DFB/C$_6$D$_6$.

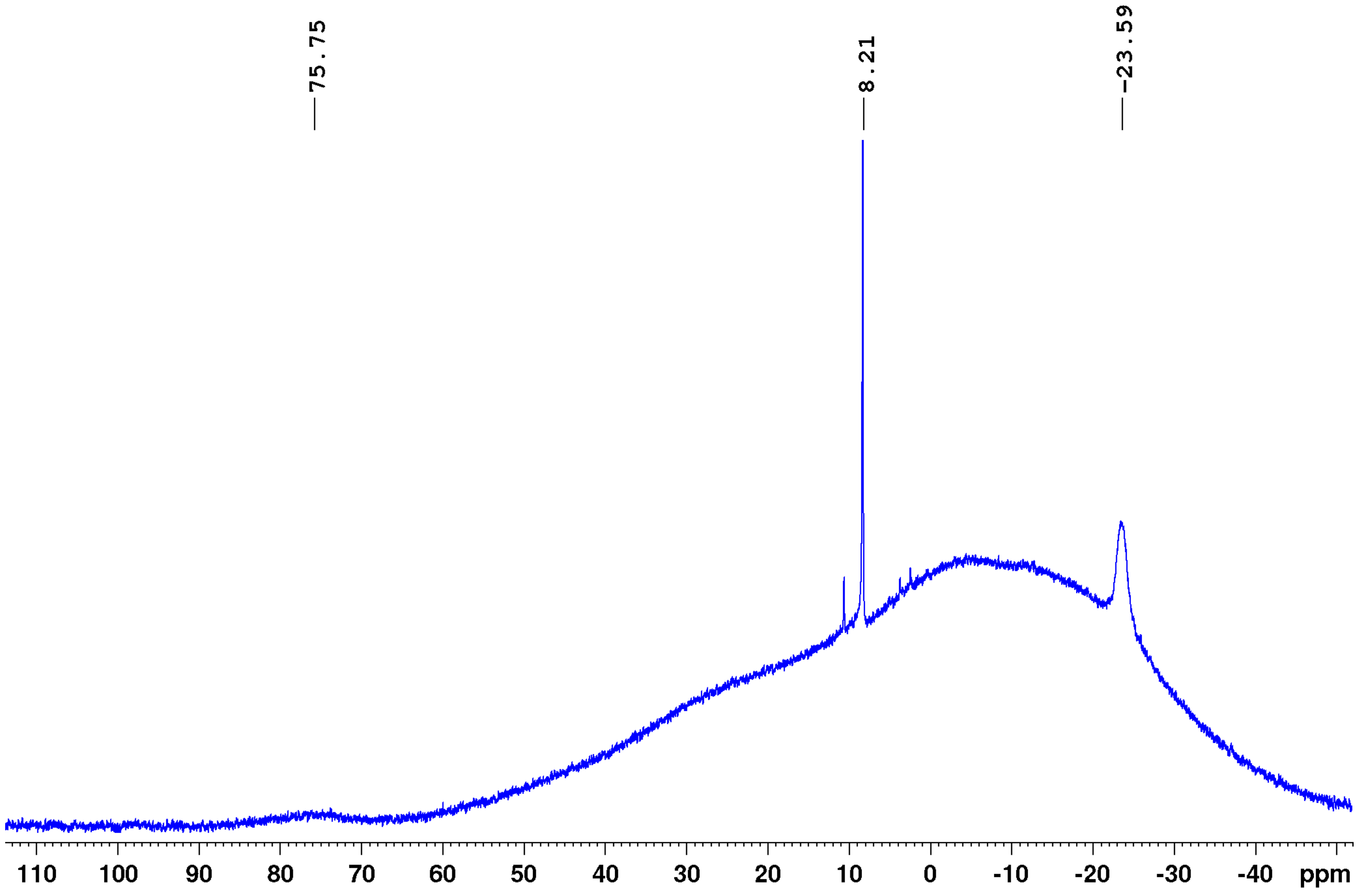

Figure S28. $^{31}P\{^{1}H\}$ NMR spectrum **[1-BCl$_2$][BCl$_4$]** in 1:10 DFB/C_6D_6.

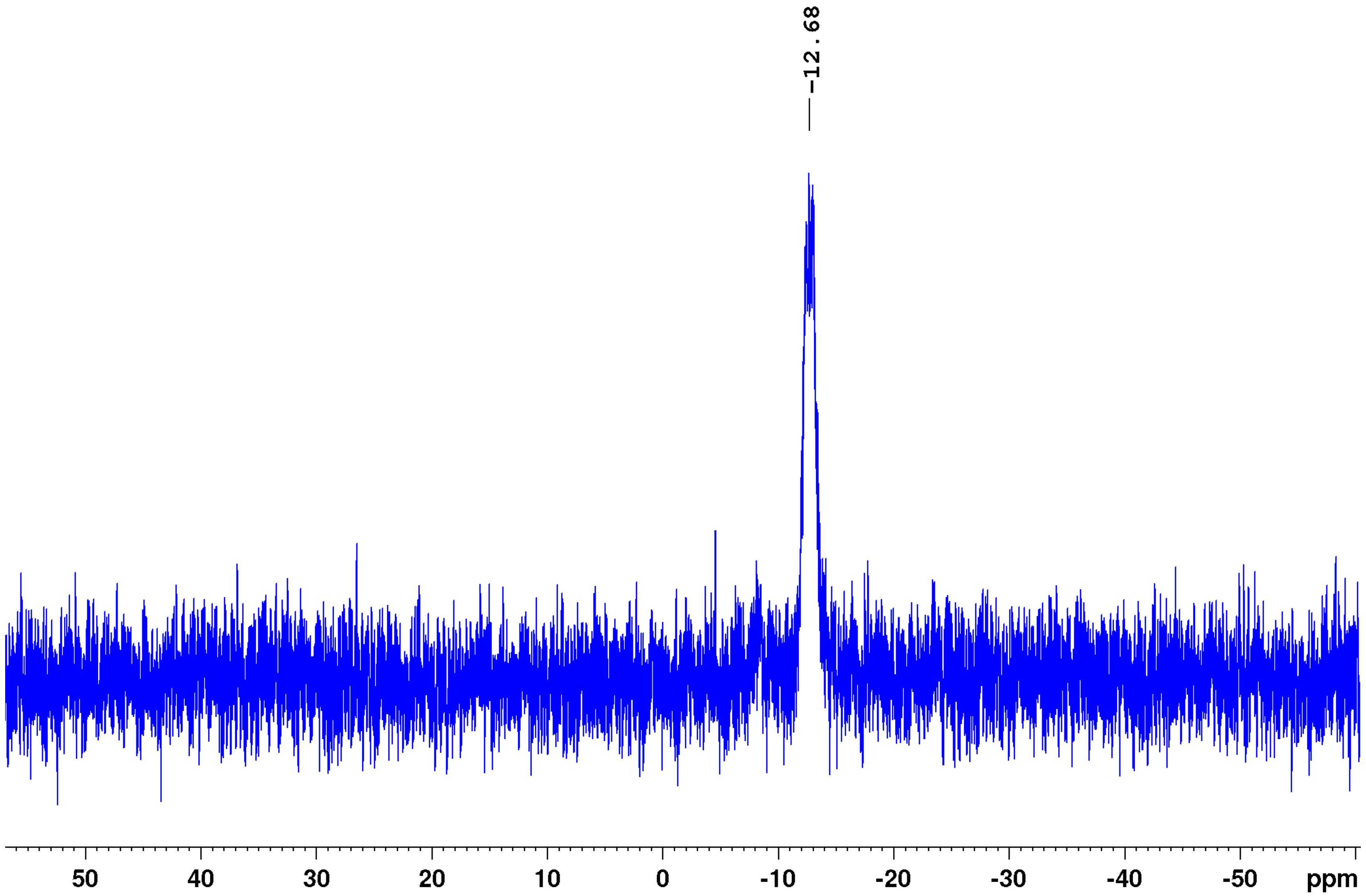

Figure S29. ^{11}B NMR spectrum of **4** in CH_2Cl_2.

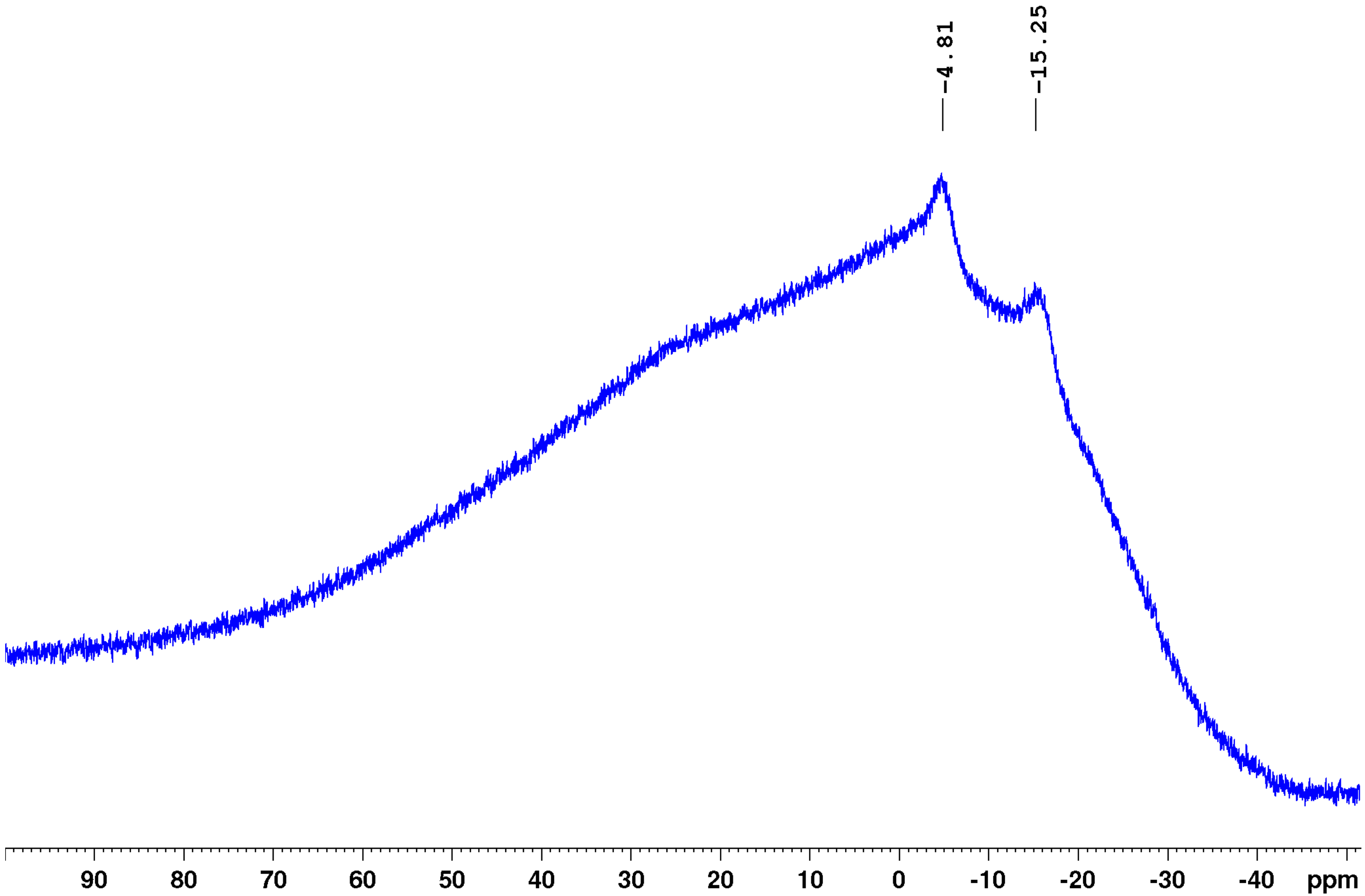

Figure S30. $^{1}H\{^{11}B\}$ NMR spectrum of **[1-I]I** in $CDCl_3$. The additional resonances at 0.88 (t) and 1.26 (m) ppm belong to residual hexane from crystallization.

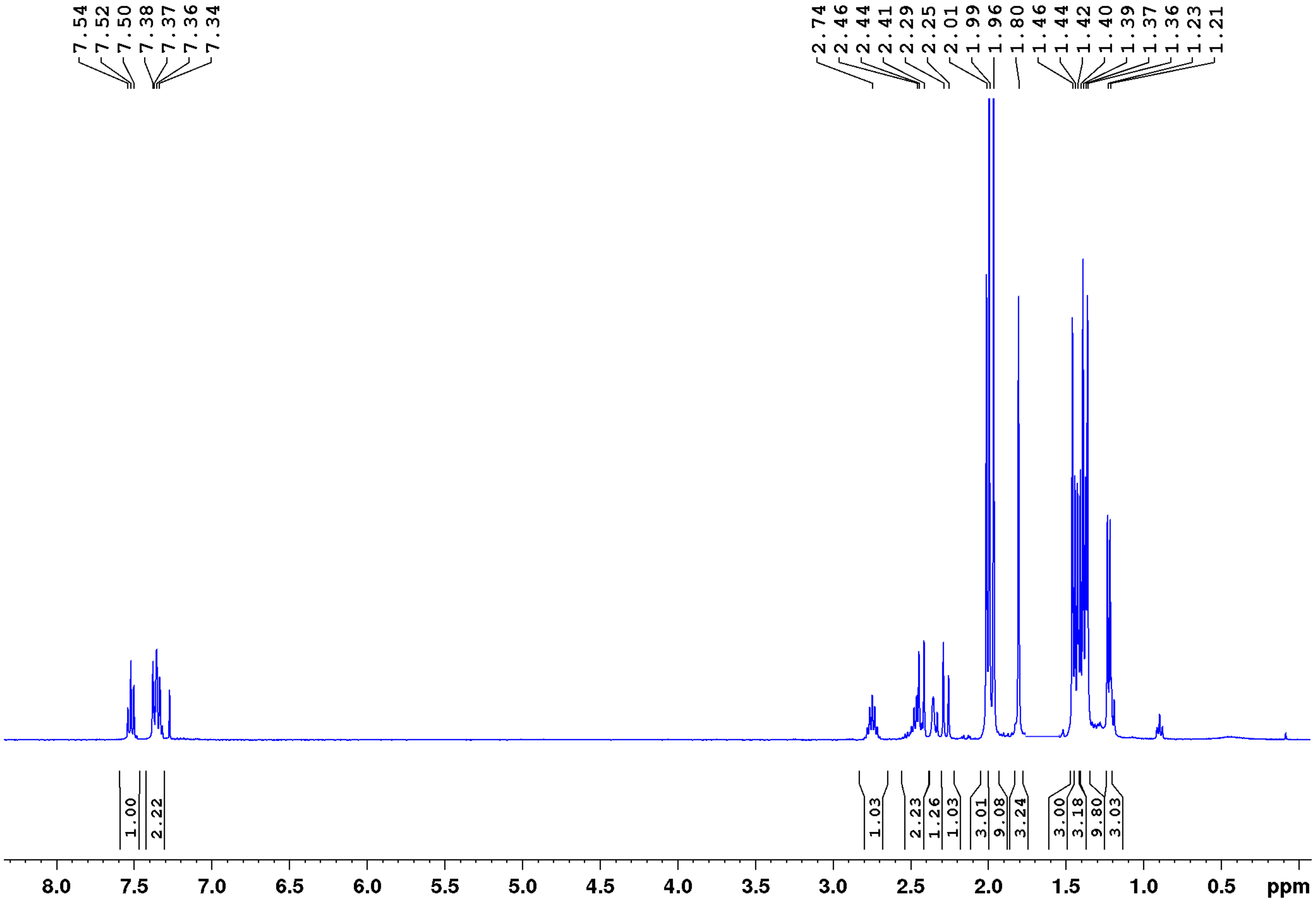

Figure S31. $^{13}C\{^1H\}$ NMR spectrum of **[1-I]I** in $CDCl_3$.

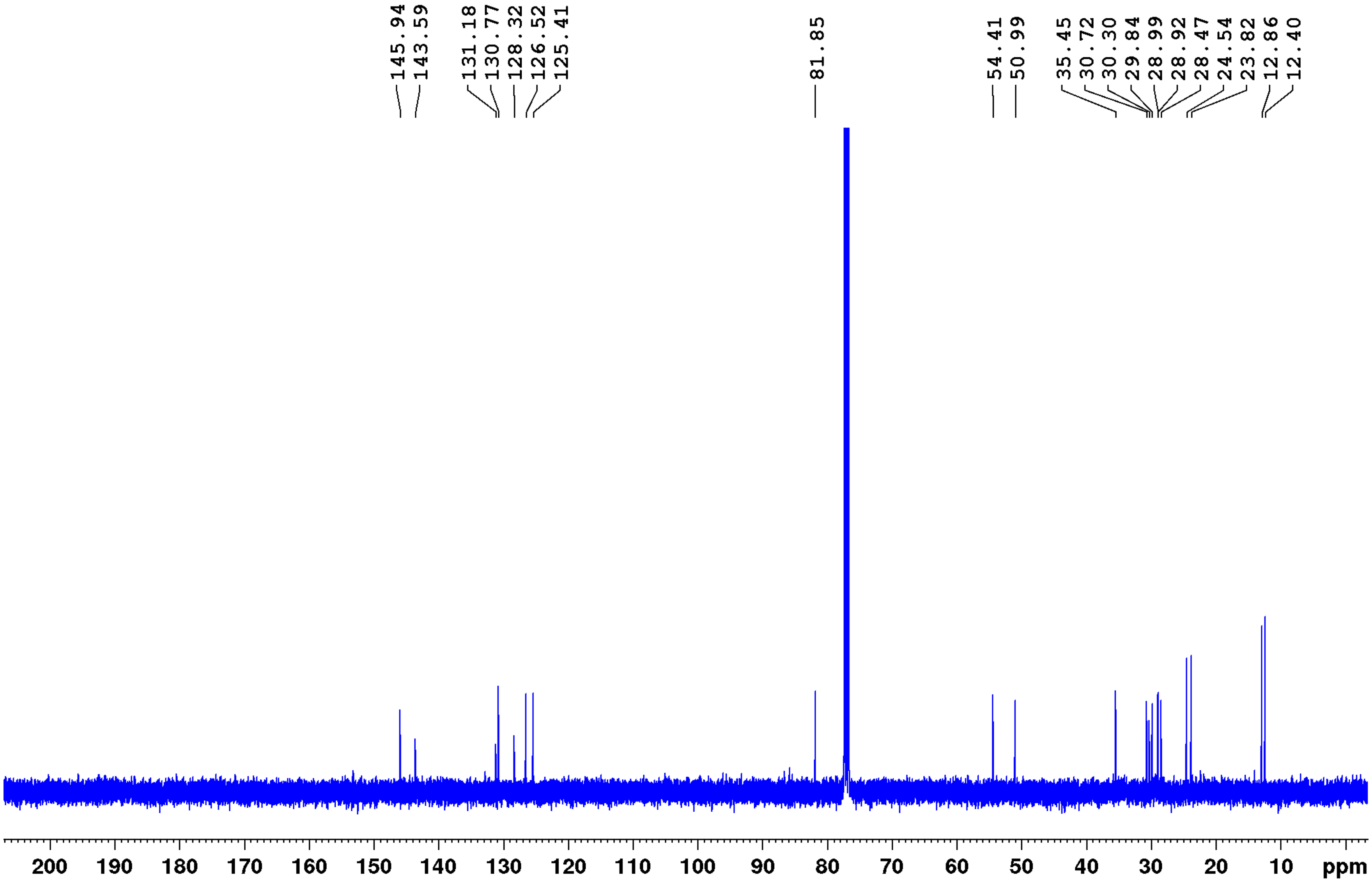

Figure S32. ^{11}B NMR spectrum of **[1-I]I** in $CDCl_3$.

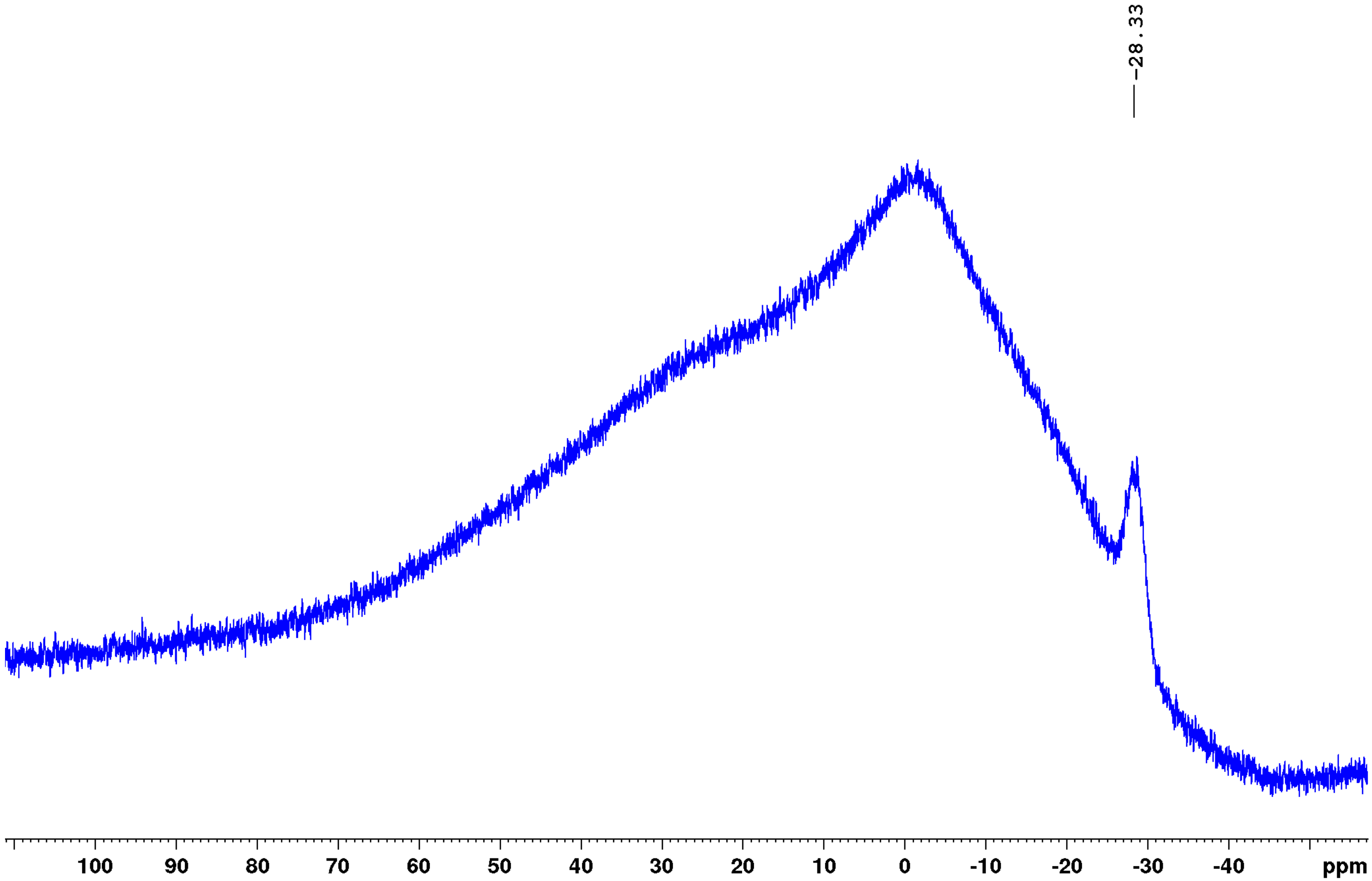

Figure S33. $^{31}P\{^{1}H\}$ NMR spectrum of **[1-I]I** in $CDCl_3$.

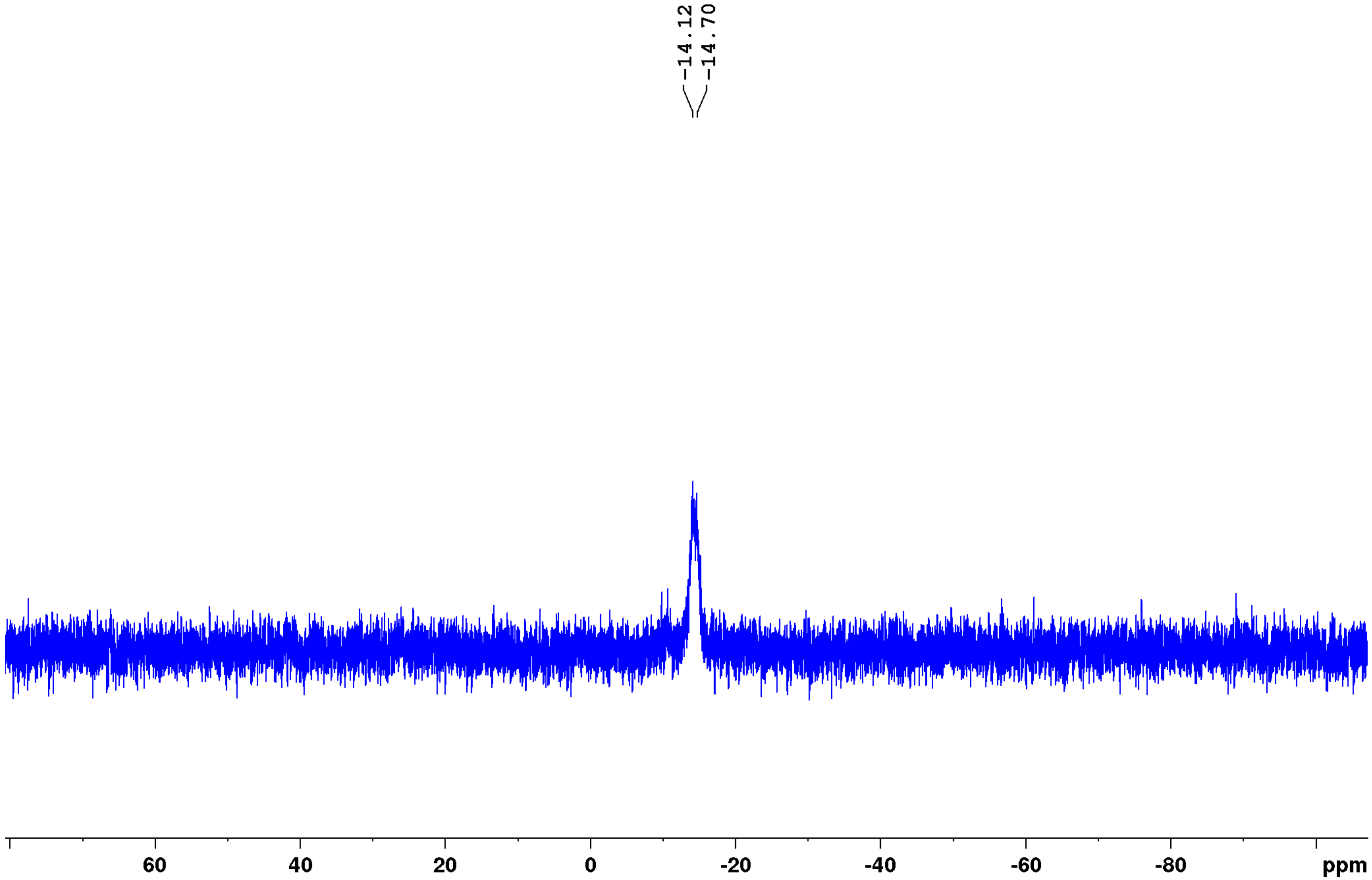

NMR-spectroscopic monitoring of the decomposition of [1-BCl_2][BCl_4] in solution

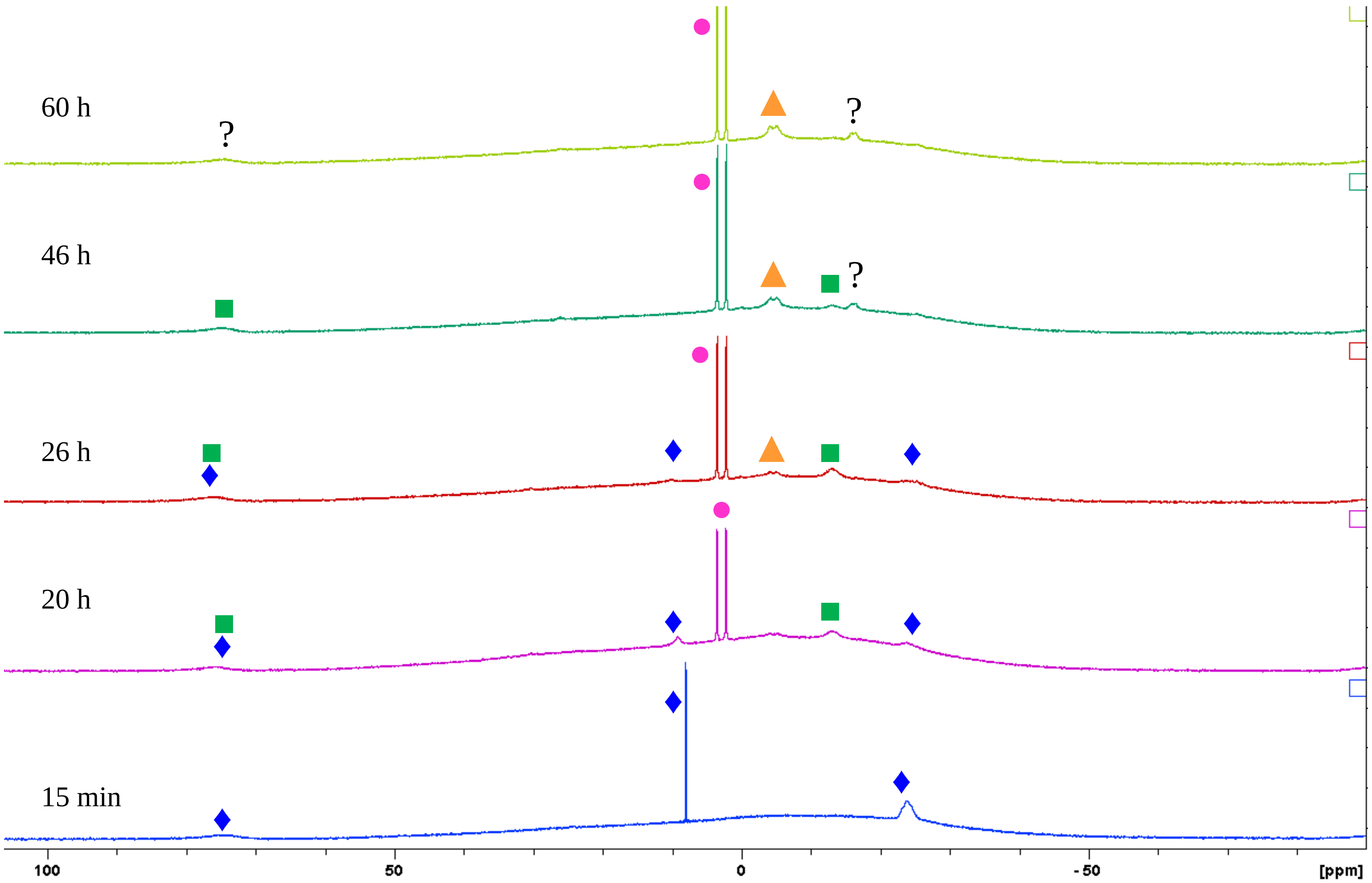

Figure S34. ^{11}B NMR-spectroscopic monitoring of the decomposition of **[1-BCl_2][BCl_4]** (♦) in 9:1 DFB/C_6D_6 at rt. Decomposition products: **2-Cl** (■), **3-Cl** (▲) and $(Me_3P)BCl_3$ (●). The ? marks an unknown sp^2-sp^3-diboron species.

Cyclic voltammetry of 1

Cyclic voltammetry experiments were performed using a Gamry Instruments Reference 600 potentiostat. A standard three-electrode cell configuration was employed using a platinum disk working electrode, a platinum wire counter electrode, and a silver wire, separated by a *Vycor* tip, serving as the reference electrode. Formal redox potentials are referenced to ferrocene by using the decamethylferrocene/decamethylferrocenium couple as an internal standard. Tetra-*n*-butylammonium hexafluorophosphate ([*n*-Bu_4N][PF_6]) was employed as the supporting electrolyte. Compensation for resistive losses (*iR* drop) was employed for all measurements.[3]

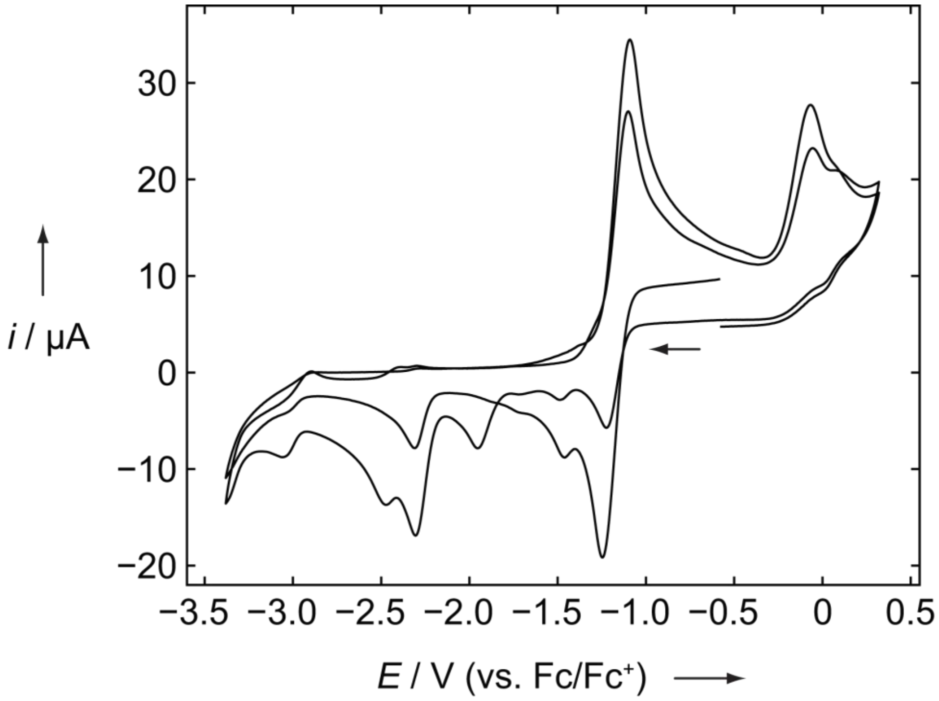

Figure S35. Cyclic voltammogram of **1** in THF with 0.1 M [*n*Bu_4N][PF_6] as the supporting electrolyte. Two consecutive cycles are shown. Formal potentials: $E_{1/2}$ = −1.15 V, E_{pa} = +0.06 V (oxidations), E_{pc} = −2.19 V, E_{pc} = −2.91 V (reductions).

EPR spectroscopy

EPR measurements at X-band (9.85 GHz) were carried out using a Bruker ELEXSYS E580 CW EPR spectrometer. The spectral simulations were performed using MATLAB 9.6.0.1072779 (R2019a) and the EasySpin 5.2.25 toolbox.[4]

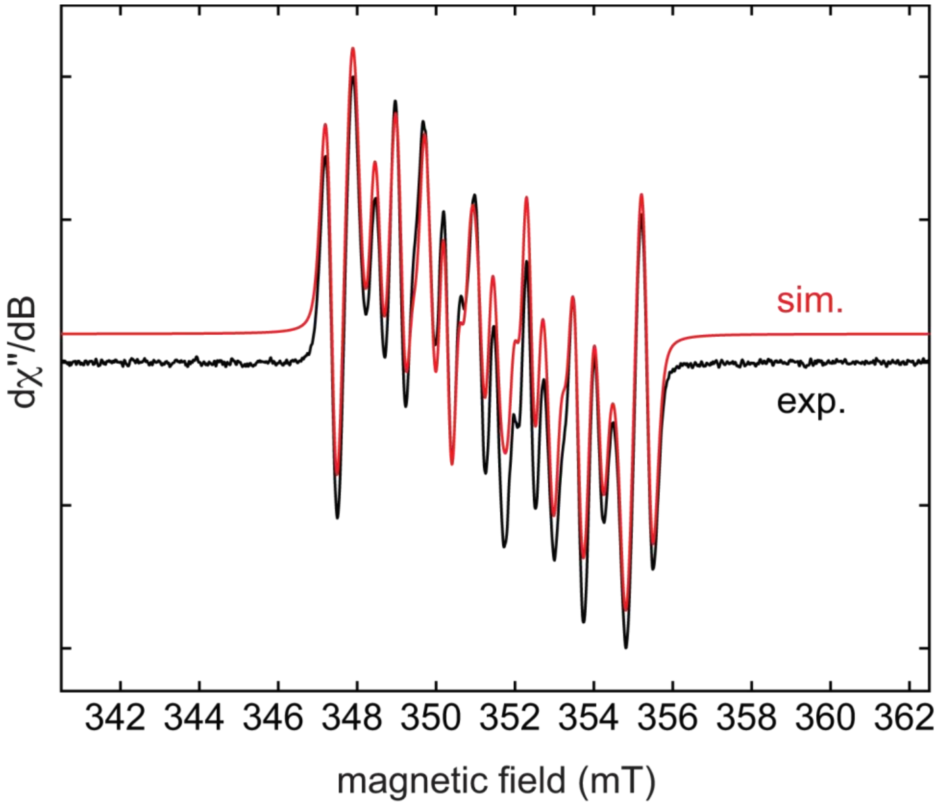

Figure S36. Experimental (black) and simulated (red) continuous-wave X-band EPR spectra of **[1][BAr$^{F}_{4}$]** in benzene at room temperature. The simulation parameters are: g_{iso} = 2.0024, a(B) = 24.4 MHz (8.7 G), a(N) = 18.1 MHz (6.5 G), a(^{1}H) = 32.1 MHz (11.5 G), and a(^{31}P) = 82.4 (29.4 G).

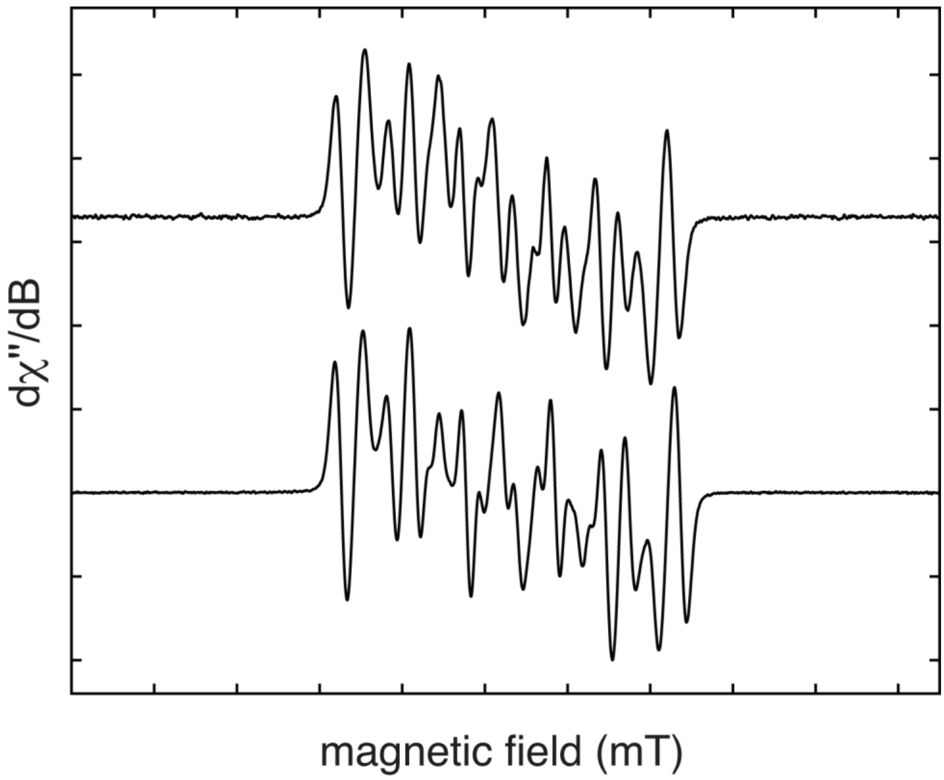

Figure S37. Comparison of room-temperature EPR spectra of the reaction mixture {**1** + $GaCl_3$} (bottom) and **[1][BAr^F$_4$]** (top), showing the formation of **1**$^{•+}$ in the former.

X-ray crystallographic details

The crystal data were collected either on a Bruker D8 Quest diffractometer with a CMOS area detector (**1-BCl$_3$·C$_6$H$_6$**, **1-BCl$_3$·DFB**, **1-InCl$_3$·DFB**, **1-InCl$_3$·CHCl$_3$**, **[1]BAr^F$_4$]**, **[1-H][In$_2$Cl$_6$]$_{0.5}$**, **[1-BCl$_2$][BCl$_4$]**, **[1-I]I),** a Bruker X8-APEX II diffractometer with a CCD area detector (**1**, **1-AlCl$_3$**, **1-GaCl$_3$**), both equipped with multi-layer mirror monochromated Mo$_{K\alpha}$ radiation, or a Rigaku OD XtaLAB Synergy-S diffractometer with a HPAD area detector, equipped with multi-layer mirror monochromated Cu$_{K\alpha}$ radiation (**[1-BF$_2$][BF$_4$]**). The structure was solved using the intrinsic phasing method,[5] refined with the ShelXL program[6] and expanded using Fourier techniques. All non-hydrogen atoms were refined anisotropically. Hydrogen atoms were included in structure factor calculations. All hydrogen atoms were assigned to idealized positions, except the boron-bound hydrogens, which were detected in the difference Fourier map and freely refined, except when stated otherwise in the refinement details.

Cif files were deposited with the Cambridge Crystallographic Data Center under CCDC numbers 2107378 ([**1][BAr^F$_4$]**), 2107379 ([**1-BCl$_2$][BCl$_4$]**), 22107380 (**1**), 2107381 (**1-AlCl$_3$**), 2107382 ([**1-BF$_2$][BF$_4$]**), 2107383 ([**1-I]I**), 2107384 (**1-InCl$_3$·CHCl$_3$**), 2107385 (**1-BCl$_3$·C$_6$H$_4$F$_2$**), 2107386 (**4**), 2107387 (**1-InCl$_3$·C$_6$H$_4$F$_2$**), 2107388 (**1-GaCl$_3$**), 2107389 ([**1-H][In$_2$Cl$_6$]$_{0.5}$**) and 107390 (**1-BCl$_3$·C$_6$H$_6$**) are available free of charge from www.ccdc.cam.ac.uk/structures.

Crystal data for 1: $C_{23}H_{41}BNP$, $M_r = 373.4$, yellow block, 0.212×0.262×0.485 mm^3, monoclinic space group $P2_1/n$, $a = 14.1867(9)$ Å, $b = 11.2719(7)$ Å, $c = 14.8047(10)$ Å, $\beta = 96.730(2)°$, $V = 2351.1(3)$ Å^3, $Z = 4$, $\rho_{calcd} = 1.055$ g·cm^{-3}, $\mu = 0.124$ mm^{-1}, $F(000) = 824$, $T = 103(2)$ K, $R_1 = 0.0787$, $wR^2 = 0.1321$, 5638 independent reflections [$2\theta \leq 55.876°$] and 250 parameters.

Crystal data for 1-BCl$_3$·C$_6$H$_6$: $C_{23}H_{41}B_2Cl_3NP·(C_6H_6)$, $M_r = 568.61$, colorless plate, 0.251×0.137×0.092 mm^3, monoclinic space group Cc, $a = 13.301(3)$ Å, $b = 15.938(3)$ Å, $c = 14.854(3)$ Å, $\beta = 92.54(3)°$, $V = 3145.8(11)$ Å^3, $Z = 4$, $\rho_{calcd} = 1.201$ g·cm^{-3},

μ = 0.361 mm^{-1}, F(000) = 1216, T = 100(2) K, R_1 = 0.0405, wR^2 = 0.0690, 7384 independent reflections [2σ ≤ 55.636°] and 340 parameters.

Crystal data for 1-BCl$_3$·DFB: $C_{23}H_{41}B_2Cl_3NP\cdot(C_6H_4F_2)$, M_r = 599.56, colorless plate, 0.392×0.29×0.086 mm^3, monoclinic space group $P2_1$, a = 9.6201(14) Å, b = 10.4564(15) Å, c = 16.299(3) Å, β = 101.730(6)°, V = 1605.3(4) Å^3, Z = 2, ρ_{calcd} = 1.240 g·cm^{-3}, μ = 0.366 mm^{-1}, F(000) = 630, T = 100(2) K, R_1 = 0.0394, wR^2 = 0.0827, 7011 independent reflections [2θ ≤ 54.212°] and 358 parameters.

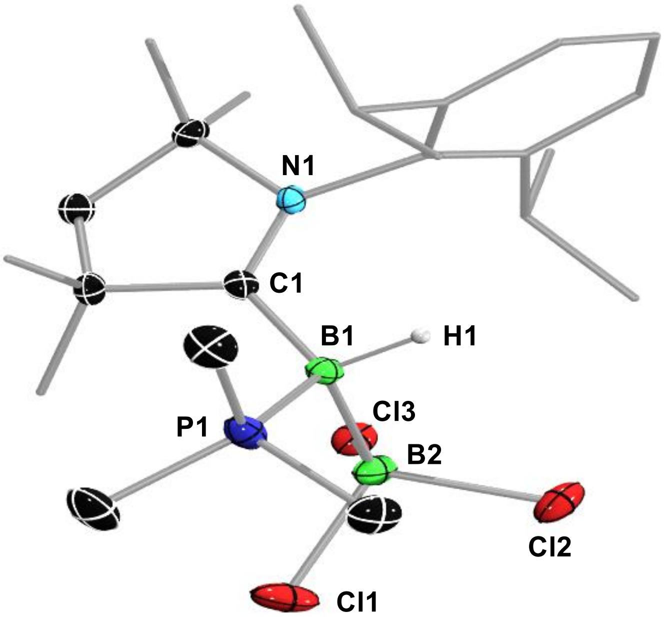

Figure S38. Crystallographically-derived molecular structure of **1-BCl$_3$**. Thermal ellipsoids set at 50% probability. Thermal ellipsoids of ligand periphery and hydrogen atoms omitted for clarity, except for boron-bound H1.

Refinement details for 1-AlCl$_3$: The asymmetric unit contains 1.5 DFB molecules. The fully occupied solvent molecule was twofold disordered and modelled with FVAR in a 61:39 ratio, with ADP restraints of SIMU 0.005. The half solvent molecule was positioned on an inversion center and at least twofold disordered. Failure to model this disorder led to the use of the Platon programm Squeeze[7] to remove this half molecule of *o*-difluorobenzene.

Crystal data for 1-AlCl$_3$: $C_{23}H_{41}AlBCl_3NP\cdot(C_6H_4F_2$ + squeezed solvent), M_r = 620.77, colorless block, 0.317×0.222×0.203 mm^3, monoclinic space group $P2_1/n$, a = 10.4743(4) Å,

b = 11.0391(4) Å, c = 29.7949(11) Å, β = 94.260(2)°, V = 3435.6(2) Å^3, Z = 4, ρ_{calcd} = 1.200 g·cm^{-3}, μ = 0.368 mm^{-1}, F(000) = 1312, T = 100(2) K, R_1 = 0.0623, wR^2 = 0.1093, 7100 independent reflections [$2\theta \leq 53.03°$] and 419 parameters.

Refinement details for 1-GaCl₃: The asymmetric unit contains 1.5 DFB molecules, the rings of which were idealized using AFIX 66. The first solvent molecule is twofold disordered modelled with free variables in a 39:61 ratio, and its atom ADPs were restrained using SIMU 0.005. The half solvent molecule lies next to an inversion center and no further restraints were applied.

Crystal data for 1-GaCl₃: $C_{23}H_{41}BCl_3GaNP \cdot (C_6H_4F_2)_{1.5}$, M_r = 720.55, yellow block, 0.632×0.593×0.47 mm^3, monoclinic space group $P2_1/n$, a = 10.527(6) Å, b = 11.112(5) Å, c = 29.971(13) Å, β = 94.434(13)°, V = 3495(3) Å^3, Z = 4, ρ_{calcd} = 1.369 g·cm^{-3}, ⎛= 1.101 mm^{-1}, F(000) = 1500, T = 296(2) K, R_1 = 0.0403, wR^2 = 0.0690, 7791 independent reflections [$2\theta \leq$ 5 4.508°] and 467 parameters.

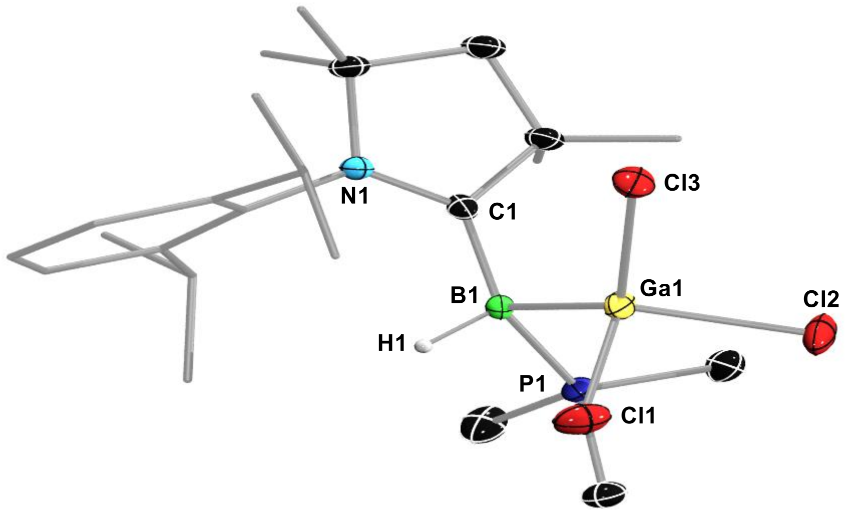

Figure S39. Crystallographically-derived molecular structure of **1-GaCl₃**. Thermal ellipsoids set at 50% probability. Thermal ellipsoids of ligand periphery and hydrogen atoms omitted for clarity, except for boron-bound H1.

Refinement details for 1-InCl$_3$·DFB: The asymmetric unit contains 1.5 DFB molecules. The first was twofold disordered in a 47:53 ratio, its ADPs restrained with SIMU 0.005. The half solvent molecule was found close to an inversion center and modelled with AFIX 66 for the ring and no further restraints.

Crystal data for 1-InCl$_3$·DFB: $C_{23}H_{41}BCl_3InNP\cdot(C_6H_4F_2)_{1.5}$, M_r = 765.65, colorless block, 0.168×0.275×0.305 mm^3, monoclinic space group $P2_1/n$, a = 10.5865(3) Å, b = 11.1545(3) Å, c = 29.8824(9) Å, β = 94.6410(10)°, V = 3517.16(17) Å^3, Z = 4, ρ_{calcd} = 1.446 g·cm^{-3}, μ = 0.984 mm^{-1}, F(000) = 1572, T = 104(2) K, R_1 = 0.0288, wR^2 = 0.0524, 8388 independent reflections [$2\theta \leq 55.836°$] and 479 parameters.

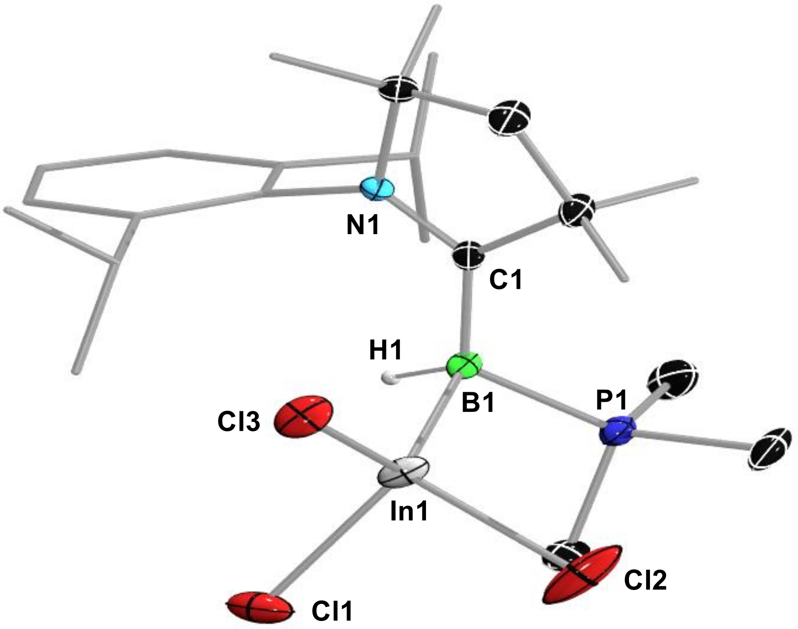

Figure S40. Crystallographically-derived molecular structure of **1-InCl$_3$·DFB**. Thermal ellipsoids set at 50% probability. Thermal ellipsoids of ligand periphery and hydrogen atoms omitted for clarity, except for boron-bound H1.

Crystal data for 1-InCl$_3$·CHCl$_3$: $C_{23}H_{41}BCl_3InNP\cdot CHCl_3$, M_r = 713.88, colorless block, 0.19×0.112×0.07 mm^3, Monoclinic space group $P2_1/n$, a = 9.650(10) Å, b = 16.465(18) Å, c = 20.33(2) Å, β = 92.06(3)°, V = 3228(6) Å^3, Z = 4, ρ_{calcd} = 1.469 g·cm^{-3}, μ = 1.293 mm^{-1}, F(000) = 1456, T = 100(2) K, R_1 = 0.1150, wR^2 = 0.2283, 6629 independent reflections [$2\theta \leq 53.108°$] and 319 parameters.

Refinement details for [1][BAr$^F_4$]: The BAr$^F{}_4^-$ counteranion shows one CF_3 group with a threefold rotational disorder (RESI 21, 22, 23 CF3), modelled with 3 FVAR summed up to 1 in a 66:14:20 ratio. SAME, SIMU 0.005 and ISOR 0.005 restraints were applied to these residues.

Crystal data for [1][BAr$^F_4$]: $C_{55}H_{53}B_2F_{24}NP$, M_r = 1236.57, colorless block, 0.345×0.246×0.15 mm^3, monoclinic space group $P2_1/c$, a = 17.484(6) Å, b = 17.632(9) Å, c = 19.038(5) Å, β = 105.73(3)°, V = 5649(4) Å^3, Z = 4, ρ_{calcd} = 1.454 g·cm^{-3}, μ = 0.166 mm^{-1}, F(000) = 2524, T = 100(2) K, R_1 = 0.0597, wR^2 = 0.0977, 12454 independent reflections [$2\theta \leq 54.26°$] and 838 parameters.

Refinement details for [1-H][In$_2$Cl$_6$]$_{0.5}$: The B-H distances to H1 and H2 were restrained with DFIX 1.2. The crystal was a pseudomerohedral twin with domains rotated by 179.8° around real axis [1.000 0.001 0.000]. The BASF parameter was refined to 50% and the structure refined with HKLF 5. The data was truncated at 0.85 Å since the crystal did not diffract sufficiently beyond that resolution due to its very small size. The displacement parameters of atoms P1, B1, N1, C6 > C8 , C9, C10 were restrained to the same value with similarity restraint SIMU. The structure provides sufficient proof of connectivity but may not be further discussed.

Crystal data for [1-H][In$_2$Cl$_6$]$_{0.5}$: $C_{29}H_{45}BCl_3F_2InNP$, M_r = 708.61, colorless needle, 0.376×0.058×0.054 mm^3, monoclinic space group $P2_1/n$, a = 10.4633(16) Å, b = 18.023(3) Å, c = 17.724(3) Å, β = 94.773(5)°, V = 3330.7(9) Å^3, Z = 4, ρ_{calcd} = 1.413 g·cm^{-3}, μ = 1.029 mm^{-1}, F(000) = 1456, T = 100(2) K, R_1 = 0.1199, wR^2 = 0.2140, 6402 independent reflections [$2\theta \leq 49.426°$] and 361 parameters.

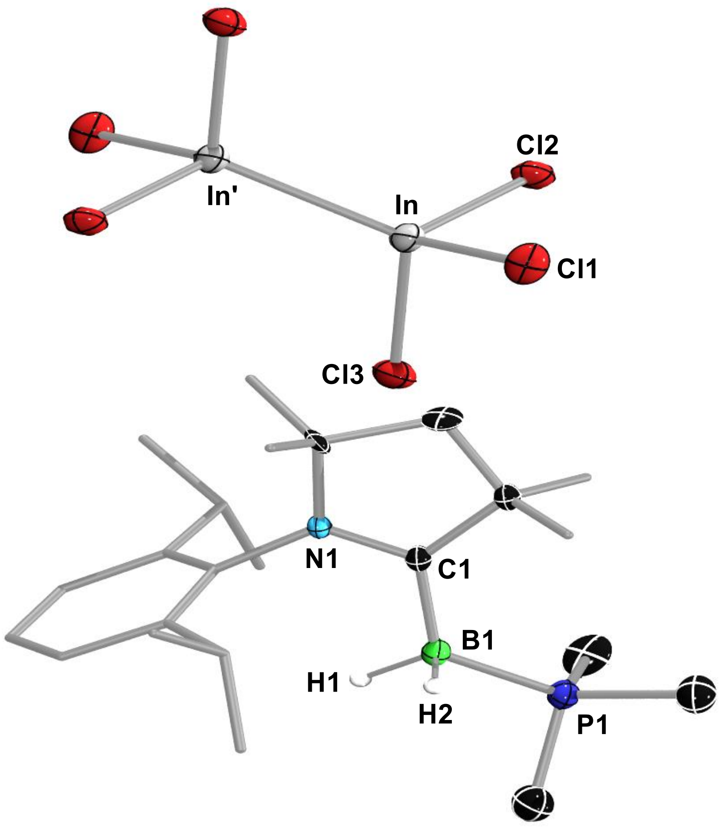

Figure S41. Crystallographically-derived molecular structure of **[1-H][In_2Cl_6]$_{0.5}$**. Thermal ellipsoids set at 50% probability. Thermal ellipsoids of ligand periphery and hydrogen atoms omitted for clarity, except for boron-bound H1.

Refinement details for [1-BF_2][BF_4]: The data were refined as a 2-component twin using the TWIN keyword (matrix: TWIN -1 0 0 0 -1 0 0.937 0.592 1). The BASF parameter was refined to 19%. The asymmetric unit contains half a benzene molecule on an inversion center, which was modelled as twofold disordered (RESI 31 and 32 Benz) by rotation around the inversion center in a 64:36 ratio. The 1-2 and 1-3 distances in these residues were restrained with SAME and the ADPs with ISOR 0.01. The 2nd BF_4^- anion in the asymmetric unit (RESI 21 and 22 BF4) was modelled as twofold disordered in a 57:43 ratio. The 1-2 and 1-3 distances in these residues were restrained with SAME and the ADPs with SIMU 0.01.

The CAAC backbone of the 2nd cation (RESI 13 and 14 CAAC) was modelled as twofold disordered in a 88:12 ratio. The 1-2 and 1-3 distances in these residues were restrained with SAME and the ADPs with SIMU 0.003.

Crystal data for [1-BF_2][BF_4]: $(C_{23}H_{41}B_3F_6NP)_2{\cdot}(C_6H_6)_{0.5}$, M_r = 1056.99, colorless plate, 0.175×0.065×0.049 mm^3, triclinic space group $P\bar{1}$, a = 13.6778(5) Å, b = 14.3470(4) Å,

c = 16.1596(7) Å, α = 101.670(3), β = 110.892(4), γ = 98.747(3), V = 2812.9(2) Å^3, Z = 2, ρ_{calcd} = 1.248 g·cm^{-3}, μ = 1.336 mm^{-1}, F(000) = 1122, T = 100(2) K, R_1 = 0.0848, wR^2 = 0.2323, 11107 independent reflections [2$\theta \leq$ 78.356°] and 809 parameters.

Crystal data for [1-BCl₂][BCl₄]: $C_{23}H_{41}B_3Cl_6NP{\cdot}(C_6H_4F_2)$, M_r = 721.76, colorless block, 0.442×0.365×0.161 mm^3, orthorhombic space group $Pca2_1$, a = 29.6071(16) Å, b = 9.4059(11) Å, c = 12.9189(8) Å, V = 3597.7(5) Å^3, Z = 4, ρ_{calcd} = 1.333 g·cm^{-3}, μ = 0.554 mm^{-1}, F(000) = 1504, T = 100(2) K, R_1 = 0.0347, wR^2 = 0.0708, 7651 independent reflections [2$\theta \leq$ 54.196°] and 394 parameters.

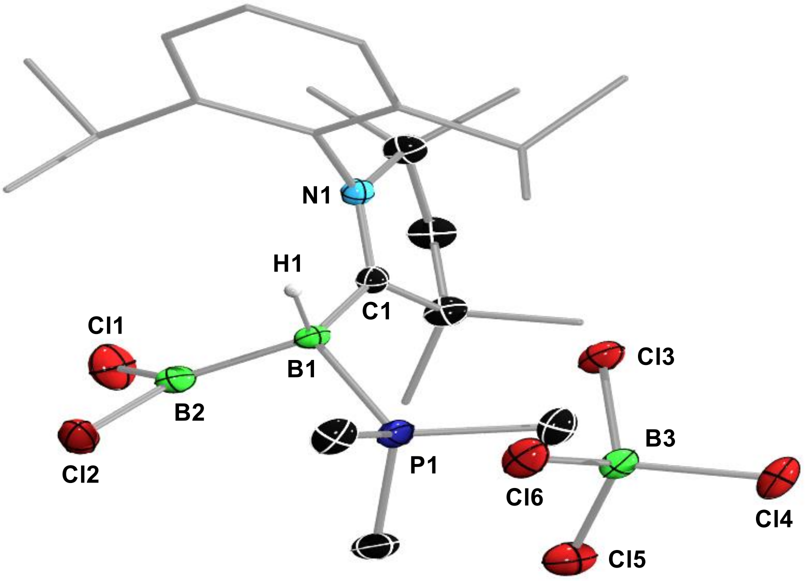

Figure S42. Crystallographically-derived molecular structure of **[1-BCl₂][BCl₄]**. Thermal ellipsoids set at 50% probability. Thermal ellipsoids of ligand periphery and hydrogen atoms omitted for clarity, except for boron-bound H1.

Refinement details for 4: The displacement parameters of atoms N1, C1 and B1 were restrained to the same value with similarity restraint SIMU.

Crystal data for 4: $C_{46}H_{82}B_4Br_6N_2P_2$, M_r = 1247.77, colorless plate, 0.040 x 0.111 x 0.126 mm^3, orthorhombic space group $Pbca$, a = 17.5828(6) Å, b = 14.5906(5) Å, c = 20.6510(6) Å,

V = 5297.9(3) Å^3, Z = 4, ρ_{calcd} = 1.564 g·cm^{-3}, μ = 4.641 mm^{-1}, F(000) = 2528, T = 100(2) K, R_1 = 0.0616, wR^2 = 0.0690, 5272 independent reflections [2$\theta \leq$ 52.246°] and 285 parameters.

Crystal data for [1-I]I: $C_{32}H_{50}BI_2NP$, M_r = 744.31, colorless block, 0.449×0.464×0.553 mm^3, triclinic space group $P\bar{1}$, a = 15.661(2) Å, b = 16.404(2) Å, c = 16.673(2) Å, α = 66.671(4)°, β = 62.158(3)°, γ = 81.970(3)°, V = 3472.5(8) Å^3, Z = 4, ρ_{calcd} = 1.424 g·cm^{-3}, μ = 1.877 mm^{-1}, F(000) = 1500, T = 100(2) K, R_1 = 0.0727, wR^2 = 0.1228, 15452 independent reflections [2θ $\leq$ 54.496°] and 697 parameters.

Computational details

Geometry optimizations and harmonic frequency calculations were performed using the ORCA program package[8] (Version 4.1.2) employing the PBEh-3c density functional composite method[9] combined with a higher integration grid (Grid5) to avoid spurious imaginary frequencies. Optimized structures were characterized as minima or first order saddle points by eigenvalue analysis of the computed Hessians. Single point calculations were conducted on the optimized geometries using the DSD-BLYP-D3BJ double hybrid density functional[10-11] as implemented in ORCA version 4.2.1 in conjunction with the def2-QZVPP basis set.[12] Additionally, the RI and RIJCOSX approximations were used for the MP2 and HF part with the respective auxiliary basis sets.[13-14]

For selected structures, relative energies from correlated wavefunction theory were computed on a smaller model system. For a smaller system with H-truncated nitrogen, phosphorus and carbon atoms single-point energies using the explicitly correlated coupled-cluster ansatz[15] CCSD(T)-F12b[16] as implemented in the Molpro2015.1 program[17] in combination with the F12-optimized correlation consistent polarized triple-zeta orbital[18] and auxiliary[19-21] basis sets of the cc-pVTZ-F12 family for all atoms.

Energy decomposition analysis (EDA) calculations were performed using the ADF2019[22-23] program employing the BP86 density functional[24-25] with the TZ2P Slater-type basis set[26] on optimized PBEh-3c structures.

Orbital composition analysis calculations were performed on the DSD-BYLP-D3BJ/def2-QZVPP//PBEh-3c wavefunction using the Multiwfn[27] program (Version 3.7) employing the Becke method. Additional results employing the Hirshfeld method are shown for comparison.

Pictures of molecular structures were generated with the Cylview[28] and ChemCraft[29] programs. All energies given are relative free energies at 298.15 K and 1 atm (ΔG^{298}) in kcal mol^{-1}.

Evaluation of the DSD-BLYP-D3BJ/def2-QZVPP//PBEh-3c method

Table S1: Comparison of ΔH^0 single point energies from CCSD(T)-F12/cc-pVTZ-F12 and DSD-BLYP-D3BJ/def2-QZVPP based on PBEh-3c optimized structures for the H-truncated model **1m**.

	CCSD(T)–F12	DSD-BLYP-D3BJ
**1m** + BF_3	–15.8	–15.0
**1m** + BH_3	–27.3	–26.7
**1m** + BMe_3	–9.3	–8.6
**1m** – PH_3	33.4	34.5
**1m** + H^+ (B)	–260.5	–260.4
**1m** + H^+ (N)	–236.5	–235.5

Additional Information on the 1-EX_3 adducts

Table S2: ΔE, ΔH^0 and selected structural parameters for the adducts of **1** with BX_3 Lewis acids (RI-DSD-BLYP-D3BJ/def2-QZVPP//PBEh-3c, energies in kcal mol^{-1}).

	ΔE	ΔH^0	B–B (Å)	Σ°(B^H)	Σ°(B^{LA})	B–X (Å)
**1**-BF_3	–13.9	–11.3	1.89	339.2	327.9	1.40
**1**-BCl_3	–24.8	–21.8	1.81	330.0	319.5	1.88
**1**-BBr_3	–30.9	–27.8	1.81	327.0	315.9	2.05
**1**-BI_3	–34.0	–31.0	1.81	325.2	311.5	2.30

Table S3: ΔE, ΔH^0 and selected structural parameters for the adducts of **1** with ECl_3 Lewis acids (RI-DSD-BLYP-D3BJ/def2-QZVPP//PBEh-3c, energies in kcal mol^{-1}).

	ΔE	ΔH^0	B–E (Å)	Σ°(B^H)	Σ°(E^{LA})	E–Cl (Å)
**1**-BCl_3	–24.8	–21.8	1.81	330.0	319.5	1.88
**1**-$AlCl_3$	–43.8	–40.7	2.23	341.1	322.6	2.17
**1**-$GaCl_3$	–52.0	–48.8	2.19	340.4	318.5	2.22
**1**-$InCl_3$	–59.5	–56.4	2.36	344.0	318.9	2.41

Table S4: Energy decomposition analysis of **1-BX$_3$** adducts, EDA components (BP86/TZ2P, energies in kcal mol^{-1})

	E_{Int}	E_{Pauli}	E_{Elec}	E_{Orb}	E_{Disp}	E_{Prep}	E_{Prep}(**1**)	E_{Prep}(BX_3)
**1**-BF_3	–72.5	136.5	–91.2	–107.6	–10.2	53.8	13.0	40.7
**1**-BCl_3	–97.9	207.8	–119.9	–164.8	–21.0	68.5	23.9	44.6
**1**-BBr_3	–108.3	213.9	–122.4	–175.5	–24.2	70.6	26.7	43.9
**1**-BI_3	–122.0	228.39	–128.0	–192.9	–29.5	73.6	29.4	44.2

Table S5: Energy decomposition analysis of **1-ECl$_3$** adducts, EDA components (BP86/TZ2P, energies in kcal mol^{-1})

	E_{Int}	E_{Pauli}	E_{Elec}	E_{Orb}	E_{Disp}	E_{Prep}	E_{Prep}(**1**)	E_{Prep}(ECl_3)
**1**-BCl_3	–97.9	207.8	–119.9	–164.8	–21.0	68.5	23.9	44.6
**1**-$AlCl_3$	–91.2	113.3	–92.1	–89.9	–22.5	39.6	15.0	24.6
**1**-$GaCl_3$	–97.7	148.2	–115.1	–107.8	–23.1	42.5	16.9	25.6
**1**-$InCl_3$	–98.6	146.9	–125.7	–95.4	–24.4	33.8	14.7	19.0

Potential scan for the ECl$_3$ Lewis acids

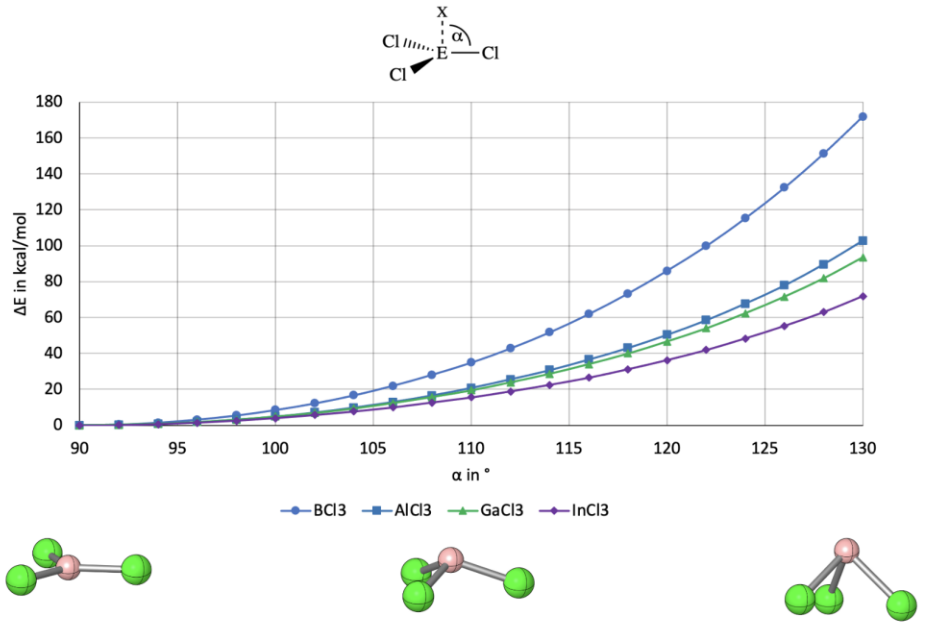

Figure S43. Scan of the C_{3v} bending potential for the ECl$_3$ Lewis acids as a function of α at the RI-DSD-BLYP-D3BJ/def2-QZVPP//PBEh-3c level of theory.

Table S6: Contributions to the HOMO of **1** according to Orbital composition analysis calculations on the DSD-BYLP-D3BJ/def2-QZVPP//PBEh-3c wavefunction.

	B	$C^{carbene}$	N	P
Becke	35%	21%	17%	11%
Hirshfeld	32%	19%	15%	8%

Table S7. Cartesian coordinates of calculated compounds. Compound (point group): total energy/Hartree (PBEh-3c).

**1m** (C_1): E_{tot} = -579.01699991			
C	-0.48503	-0.40021	0.69351
C	-0.08748	-1.86145	0.76731
H	-0.94944	-2.49233	0.52809
H	0.26954	-2.16889	1.75067
C	0.97997	-2.01671	-0.3189
H	1.0496	-3.02923	-0.71505
H	1.96016	-1.73899	0.07251
C	0.53534	-0.99814	-1.364
H	-0.18657	-1.44291	-2.06449
H	1.36832	-0.61211	-1.9563
N	-0.04711	0.02257	-0.53423
B	-1.19148	0.44113	1.65165
P	-1.62333	-0.3245	3.27858
H	-2.94442	-0.69965	3.63446
H	-1.34583	0.40413	4.45403
H	-0.98181	-1.53837	3.56589
H	-1.52225	1.58079	1.50365
H	-0.36602	0.89457	-0.91515

**BF_3** (D_{3h}): E_{tot} = -323.93762507			
B	0	0	0.99998
F	1.13667	0	1.65629
F	0	0	-0.31258
F	-1.13667	0	1.65629

**1m+BF_3** (C_1): E_{tot} = -902.99111622			
C	-0.65577	-0.40034	0.5933
C	0.02046	-1.72066	0.83228
H	-0.73277	-2.51242	0.75763
H	0.46705	-1.78278	1.82195
C	1.05151	-1.81443	-0.29605
H	1.25991	-2.83597	-0.60832
H	1.98582	-1.3615	0.03517
C	0.43377	-0.97168	-1.41179
H	-0.1351	-1.57325	-2.12699
H	1.17372	-0.39749	-1.96936
N	-0.44897	-0.08894	-0.67001
B	-1.27714	0.54462	1.58943
P	-1.77516	-0.17375	3.26415
H	-2.85712	-1.05916	3.4404
H	-2.12209	0.82435	4.17647
H	-0.77822	-0.87075	3.95862
H	-2.06475	1.37997	1.23685
H	-0.78587	0.78024	-1.05153
B	0.32994	1.43482	2.10484
F	1.28702	0.49651	2.49828
F	-0.01937	2.23728	3.18346
F	0.75074	2.16286	1.01267

**BH_3** (D_{3h}): E_{tot} = -26.53707793			
H	-0.11609	0	-0.1877
B	-0.05	0.00002	0.99999
H	1.01161	0	1.53663
H	-1.04552	0	1.65107

**1m+BH_3** (C_1): E_{tot} = -605.60859848			
C	-0.37646	-0.77997	0.50485
C	0.48176	-1.98402	0.22532
H	-0.0755	-2.64898	-0.44233
H	0.70413	-2.53794	1.13348
C	1.71716	-1.40116	-0.46195
H	2.18813	-2.08836	-1.16312
H	2.45312	-1.12708	0.29355
C	1.18453	-0.14059	-1.1464
H	0.95016	-0.30856	-2.20201
H	1.88642	0.693	-1.09003
N	-0.02484	0.14395	-0.39254
H	-0.40384	1.07421	-0.37329
B	-1.30912	-0.73195	1.67081
H	-1.70869	-1.73194	2.18989
P	-2.50519	0.71786	1.85754
H	-3.86746	0.63219	1.49653
H	-2.16479	1.90656	1.20168
H	-2.62439	1.15477	3.18054
B	0.09571	0.1369	2.61247
H	1.20746	-0.2533	2.32808
H	-0.181	-0.1102	3.76463
H	0.0081	1.33014	2.33964

**BMe_3** (C_1): E_{tot} = -144.27990471			
B	0.00189	-0.00684	1.00173
C	-0.00358	0.01065	-0.57262
H	0.75294	0.68987	-0.97354
H	-0.96266	0.24734	-1.03338
H	0.29307	-0.98285	-0.92839
C	1.36411	-0.11206	1.78379
H	2.24528	-0.28353	1.16561
H	1.32451	-0.88587	2.55482
H	1.52514	0.82295	2.3324
C	-1.35768	0.08506	1.79095
H	-1.27623	0.0055	2.87485
H	-2.05539	-0.68303	1.44403
H	-1.85141	1.03462	1.55975

**1m+BMe_3** (C_1): E_{tot} = -723.31839586			
C	-0.66641	-0.16194	-0.01646
H	-0.54207	0.58825	0.76558
H	-1.32767	0.2604	-0.78039
C	0.66524	-0.63076	-0.60266
H	1.45894	-0.50595	0.13414
H	0.94745	-0.07657	-1.49612
C	0.43531	-2.12331	-0.86187
H	1.3446	-2.7149	-0.76098
H	0.04265	-2.29406	-1.87017

N	-1.21306	-1.39124	0.51917
H	-1.96511	-1.40123	1.18713
C	-0.61857	-2.5181	0.13867
B	-0.90088	-3.86492	0.71536
P	-0.17849	-5.31684	-0.24302
H	1.22007	-5.37607	-0.34225
H	-0.50968	-5.62493	-1.58115
H	-0.47184	-6.54601	0.35253
H	-1.94892	-4.15366	1.21884
B	0.39923	-3.80724	2.27401
C	1.88564	-3.30923	1.85143
H	2.55415	-3.43083	2.71098
H	1.94543	-2.25104	1.57686
H	2.35911	-3.87732	1.03999
C	-0.33218	-2.81361	3.32007
H	0.23758	-2.7965	4.25657
H	-1.34548	-3.12998	3.5844
H	-0.38548	-1.77019	2.99239
C	0.41364	-5.34818	2.81306
H	0.90114	-5.35446	3.79481
H	0.99215	-6.05658	2.20736
H	-0.5804	-5.77971	2.96348

PH_3 (C_{3v}): E_{tot} = -342.73082732

H	-0.06005	-0.10401	-0.3009
P	0.10399	0.18012	1.07353
H	-1.24652	-0.10401	1.37701
H	0.53318	-1.13152	1.37701

1m–PH_3 (C_1): E_{tot} = -236.2218426

C	0.3715	-0.49976	1.0672
C	-0.11149	-0.24064	-0.35873
H	0.11752	0.7852	-0.65883
H	-1.18143	-0.39169	-0.499
C	0.73885	-1.2163	-1.17763
H	0.86355	-0.89914	-2.21263
H	0.28923	-2.21031	-1.17578
C	2.04508	-1.23555	-0.39964
H	2.6546	-0.35805	-0.66125
H	2.64364	-2.12685	-0.59706
N	1.57449	-1.22257	0.97097
H	2.269	-1.03276	1.67375
B	-0.37346	-0.05675	2.16036
H	-1.05639	0.34397	3.02282

1m+H^+ (B) (C_1): E_{tot} = -579.4491895

C	0.41015	-0.58004	0.91455
C	-0.15787	-0.48943	-0.46464
H	-0.32131	0.57148	-0.68325
H	-1.13703	-0.96504	-0.53964
C	0.90297	-1.13169	-1.37039
H	1.0253	-0.60149	-2.31131
H	0.62814	-2.15858	-1.60694
C	2.17923	-1.10402	-0.52715
H	2.8358	-0.26483	-0.76202
H	2.76473	-2.02008	-0.58073
N	1.6472	-0.92521	0.82619
H	2.2353	-1.00052	1.64768
B	-0.24907	-0.16985	2.298
H	0.34924	-0.57064	3.2594
H	-0.36816	1.03031	2.31403
P	-2.07203	-0.80494	2.52473
H	-2.29078	-2.18683	2.56893
H	-3.03703	-0.38394	1.60204
H	-2.60657	-0.37275	3.74049

1m+H^+ (N) (C_1): E_{tot} = -579.40840137

C	-0.7446	0.00192	-0.00065
H	-0.55673	0.95915	0.48082
H	-1.71003	0.03735	-0.50311
C	0.35031	-0.49142	-0.92615
H	1.33385	-0.29611	-0.49459
H	0.31166	0.00774	-1.89212
C	0.07506	-1.99431	-1.01447
H	0.97945	-2.54518	-1.27424
H	-0.65528	-2.18756	-1.806
N	-0.81522	-1.08621	1.02608
H	-0.14562	-0.8985	1.7734
H	-1.73039	-1.1354	1.47171
B	-0.71151	-3.59242	1.04927
H	-1.17543	-3.72132	2.13835
P	-0.2555	-5.23307	0.18153
H	0.62451	-6.06293	0.88556
H	0.35659	-5.11666	-1.07028
H	-1.32354	-6.10037	-0.07653
C	-0.46955	-2.37954	0.33973

1 (C_1): E_{tot} = -1319.58907616

P	3.20297	0.52813	-0.8938
H	0.78417	0.78387	-1.46546
N	-0.65656	-0.32815	0.75514
C	0.72443	-0.26321	0.62674
C	-1.15825	-0.91848	2.00288
C	-2.22423	-0.05798	2.67969
H	-2.54383	-0.53177	3.60952
H	-3.10786	0.0592	2.05052
H	-1.84678	0.93174	2.92962
C	-1.76869	-2.31088	1.80803
H	-2.09503	-2.71397	2.76856
H	-1.0716	-3.02465	1.37357
H	-2.64539	-2.2604	1.16177
C	0.12289	-0.95498	2.84089
H	0.15043	-1.80961	3.52075
H	0.16791	-0.0549	3.46118
C	1.30416	-0.94593	1.8571
C	1.75293	-2.37352	1.51761
H	2.06812	-2.90923	2.41727
H	2.58935	-2.36249	0.81995
H	0.96012	-2.95166	1.04564
C	2.47519	-0.19651	2.48482
H	2.74236	-0.63828	3.44807
H	2.22882	0.85313	2.64743
H	3.37116	-0.23829	1.86544
C	-1.52149	0.13069	-0.2675
C	-1.95456	1.46864	-0.26642
C	-1.40972	2.50711	0.69241
H	-0.76966	2.00113	1.4155
C	-2.51298	3.2459	1.45067
H	-2.08077	3.91101	2.20053
H	-3.19027	2.56153	1.96046
H	-3.11577	3.8653	0.78427
C	-0.52363	3.50554	-0.05607
H	-0.08531	4.22336	0.64071
H	-1.09271	4.07039	-0.79742
H	0.28649	2.98676	-0.566
C	-2.86298	1.88012	-1.23605
H	-3.21524	2.90477	-1.23786
C	-3.31291	1.01089	-2.21172
H	-4.02595	1.34631	-2.95398
C	-2.81873	-0.27888	-2.2542
H	-3.13587	-0.94039	-3.05159
C	-1.90848	-0.73441	-1.30597
C	-1.30482	-2.1101	-1.49542
H	-0.66865	-2.32399	-0.63797
C	-0.3986	-2.12095	-2.7288
H	0.08928	-3.0923	-2.83497
H	0.37423	-1.35912	-2.63765
H	-0.96363	-1.93319	-3.64416
C	-2.35687	-3.21354	-1.60202
H	-1.87734	-4.19285	-1.64991
H	-2.9635	-3.10991	-2.50335
H	-3.03626	-3.21782	-0.75025
C	3.34028	0.97251	-2.65629
H	2.93834	0.16366	-3.26407
H	4.37298	1.16354	-2.94616
H	2.74277	1.86191	-2.84823

C	4.11002	1.8964	-0.07203
H	4.15228	1.72868	1.00228
H	3.56561	2.82438	-0.24116
H	5.126	2.00169	-0.45515
C	4.37783	-0.87166	-0.74489
H	4.50667	-1.16811	0.29411
H	5.3544	-0.60296	-1.14907
H	3.98643	-1.72619	-1.29449
B	1.38135	0.32088	-0.53312

BCl$_3$ (D_{3h}): E_{tot} = -1404.02660338

B	0	0	0.99997
Cl	1.49954	0	1.8658
Cl	0	0	-0.73157
Cl	-1.49954	0	1.8658

BBr$_3$ (D_{3h}): E_{tot} = -7743.93224055

B	0	0	0.99986
Br	1.63576	0	1.94464
Br	0	0	-0.88914
Br	-1.63576	0	1.94464

BI$_3$ (D_{3h}): E_{tot} = -918.09687947

B	0	0	1.00015
I	1.82741	0	2.05481
I	0	0	-1.10977
I	-1.82741	0	2.05481

AlCl$_3$ (D_{3h}): E_{tot} = -1621.43918326

Al	0	0	0.99998
Cl	0	0	-1.06851
Cl	-1.79143	0	2.03426
Cl	1.79143	0	2.03426

GaCl$_3$ (D_{3h}): E_{tot} = -3303.16568413

Ga	0	0	1.00002
Cl	0	0	-1.10767
Cl	-1.82535	0	2.05382
Cl	1.82535	0	2.05382

InCl$_3$ (D_{3h}): E_{tot} = -1569.41467095

In	0	0	1.0000
Cl	0	0	-1.30526
Cl	-1.99644	0	2.15263
Cl	1.99644	0	2.15263

1-BF$_3$ (C_1): E_{tot} = -1643.55530431

P	5.66968	9.73745	6.26552
N	6.93259	12.88209	8.78112
C	7.07178	11.80512	8.01524
C	8.14431	13.75767	8.88311
C	7.94651	15.07178	8.13257
H	8.86571	15.65564	8.19137
H	7.15258	15.66887	8.58029
H	7.71724	14.92904	7.07847
C	8.51346	14.0922	10.31977
H	9.44606	14.6587	10.31522
H	8.67559	13.19697	10.91595
H	7.7584	14.70988	10.80483
C	9.20609	12.87068	8.24476
H	9.76432	12.35188	9.02728
H	9.92806	13.4488	7.66536
C	8.46236	11.83673	7.39544
C	9.22254	10.51597	7.42647
H	10.22349	10.67968	7.02067
H	8.75029	9.74137	6.83285
H	9.32212	10.13653	8.43951
C	8.32725	12.30356	5.93808
H	9.31857	12.43923	5.50233
H	7.79106	13.24697	5.83889
H	7.80406	11.5675	5.32897
C	5.71177	13.37071	9.37138
C	5.45857	13.1796	10.74109
C	6.2018	12.20303	11.6281
H	7.03874	11.78104	11.07708
C	5.27001	11.04063	11.98234
H	5.8227	10.26498	12.51187
H	4.83608	10.58259	11.09927
H	4.45632	11.37602	12.62927
C	6.72817	12.83415	12.91929
H	7.33096	12.10616	13.46368
H	5.9158	13.13204	13.58433
H	7.34587	13.71417	12.74751
C	4.38619	13.85887	11.31363
H	4.18348	13.72479	12.36823
C	3.56841	14.68403	10.57136
H	2.74947	15.21162	11.04251
C	3.78263	14.79937	9.21268
H	3.10732	15.40569	8.62212
C	4.83518	14.14273	8.5851
C	4.88359	14.22779	7.07209
H	5.76443	13.69643	6.70927
C	4.93352	15.65962	6.53611
H	5.02921	15.65084	5.44903
H	5.76718	16.23145	6.93815
H	4.01977	16.2072	6.77038
C	3.65374	13.51736	6.50101
H	3.7082	13.46406	5.41228
H	2.73692	14.05063	6.75623
H	3.56957	12.50753	6.89876
C	4.90121	10.70134	4.91085
H	5.53122	11.54308	4.62707
H	3.94184	11.09484	5.23595
H	4.7391	10.07307	4.03436
C	4.3857	8.506	6.6419
H	4.79991	7.71614	7.26214
H	3.98799	8.07939	5.72148
H	3.58163	8.98328	7.19836
C	6.92048	8.77449	5.34506
H	7.42512	8.08338	6.01217
H	7.65775	9.42143	4.87358
H	6.41608	8.21002	4.56011
B	6.01635	10.6976	7.89041
H	4.9	11.03888	8.20955
B	6.55333	9.30519	9.04965
F	7.44012	9.7555	10.02988
F	5.40139	8.77823	9.62283
F	7.18207	8.28493	8.30611

1-BCl$_3$ (C_1): E_{tot} = -2723.66077393

Cl	7.44337	7.71597	8.15627
Cl	5.09177	8.43924	9.8541
Cl	7.82817	9.6812	10.3708
P	5.69756	9.7166	6.2289
N	6.93472	12.80379	8.81334
C	7.10642	11.72665	8.07252
C	8.15416	13.68937	8.92955
C	7.95032	15.01722	8.20816
H	8.86584	15.60347	8.29141
H	7.14739	15.60027	8.65819
H	7.73775	14.89749	7.14883
C	8.52664	13.99162	10.37158
H	9.47201	14.53662	10.36843
H	8.67303	13.08376	10.95223
H	7.78982	14.6217	10.86694
C	9.21892	12.81512	8.28018
H	9.76672	12.27287	9.05389
H	9.9451	13.40371	7.71719
C	8.47432	11.80674	7.4101
C	9.26043	10.50687	7.31893
H	10.22036	10.71383	6.84051
H	8.75636	9.747	6.73118
H	9.45107	10.08123	8.29992
C	8.26338	12.3479	5.98434
H	9.23586	12.51659	5.51938
H	7.71499	13.28767	5.94945
H	7.7288	11.63615	5.35812
C	5.71201	13.33816	9.38607
C	5.47232	13.26571	10.77168

C	6.15911	12.3127	11.72268
H	6.99453	11.83987	11.21513
C	5.17844	11.19548	12.08536
H	5.68811	10.41678	12.65233
H	4.75827	10.72253	11.20185
H	4.35528	11.57796	12.69341
C	6.67626	12.9688	13.00388
H	7.24984	12.23904	13.57629
H	5.86326	13.30575	13.64914
H	7.32279	13.82547	12.81918
C	4.45156	14.04553	11.30871
H	4.26348	13.9963	12.37296
C	3.6725	14.86811	10.52514
H	2.90029	15.48194	10.96967
C	3.86374	14.86529	9.15969
H	3.21461	15.46789	8.53739
C	4.85684	14.09682	8.56147
C	4.84614	14.07068	7.04547
H	5.65032	13.42574	6.68747
C	5.00743	15.44711	6.3968
H	5.04931	15.34726	5.31091
H	5.90732	15.96629	6.71775
H	4.16074	16.09611	6.6237
C	3.5189	13.45868	6.5864
H	3.49827	13.34315	5.5021
H	2.67803	14.09748	6.85774
H	3.35235	12.48668	7.04885
C	4.82594	10.81808	5.05858
H	5.38753	11.73372	4.88128
H	3.85424	11.08608	5.46176
H	4.67523	10.31071	4.10542
C	4.46878	8.4041	6.49777
H	4.93829	7.54772	6.97416
H	4.03854	8.1041	5.5421
H	3.68318	8.76567	7.15744
C	6.91549	8.93781	5.10603
H	7.49356	8.18512	5.63142
H	7.59111	9.67261	4.67345
H	6.36782	8.46152	4.29189
B	6.08979	10.52532	7.96748
H	4.97004	10.88138	8.26708
B	6.59884	9.16235	9.04804

1-BBr$_3$ (C_1): E_{tot} = -9063.56720782

P	5.71319	9.68501	6.25287
N	6.93626	12.77856	8.8236
C	7.12896	11.69513	8.09744
C	8.15793	13.66784	8.95634
C	7.95837	15.01733	8.27638
H	8.8679	15.60501	8.40317
H	7.14034	15.58179	8.7225
H	7.77841	14.93143	7.20819
C	8.53184	13.92352	10.40748
H	9.48675	14.45159	10.41872
H	8.66236	12.9974	10.96284
H	7.80653	14.55405	10.91856
C	9.22439	12.81462	8.28143
H	9.78586	12.26241	9.03797
H	9.93758	13.42008	7.71961
C	8.47867	11.81784	7.40202
C	9.29348	10.54595	7.22579
H	10.22583	10.80089	6.71698
H	8.7875	9.79948	6.62215
H	9.54491	10.08251	8.17506
C	8.20926	12.40442	6.00239
H	9.16387	12.59957	5.5116
H	7.64979	13.33756	6.0154
H	7.66305	11.7063	5.37081
C	5.71077	13.33345	9.38066
C	5.46803	13.30681	10.76797
C	6.10152	12.33456	11.73316
H	6.93114	11.83812	11.24158
C	5.06987	11.25512	12.06717
H	5.53543	10.44638	12.63002
H	4.64073	10.81735	11.16965
H	4.25434	11.66556	12.66722
C	6.6194	12.96191	13.02718
H	7.15362	12.20537	13.6027
H	5.80947	13.32611	13.66139
H	7.30363	13.7924	12.85939
C	4.48416	14.1425	11.2885
H	4.29964	14.12759	12.35433
C	3.73821	14.97768	10.4865
H	2.99766	15.63888	10.91659
C	3.91757	14.92174	9.12096
H	3.28837	15.53014	8.48418
C	4.87326	14.09476	8.53912
C	4.8354	14.0165	7.02546
H	5.59177	13.30941	6.68218
C	5.06898	15.35382	6.32033
H	5.07032	15.21193	5.23834
H	6.01077	15.82204	6.595
H	4.27537	16.06688	6.54686
C	3.46372	13.47331	6.60958
H	3.41828	13.31449	5.53165
H	2.6701	14.17643	6.8628
H	3.23819	12.53465	7.1145
C	4.77325	10.82295	5.17694
H	5.32347	11.74188	4.98406
H	3.832	11.07856	5.65293
H	4.55765	10.34023	4.22345
C	4.50884	8.34261	6.4827
H	4.98457	7.46582	6.91297
H	4.07253	8.08352	5.51756
H	3.72398	8.66755	7.16227
C	6.9009	9.00465	5.03623
H	7.53209	8.24153	5.47848
H	7.52876	9.78648	4.61429
H	6.32672	8.55802	4.22348
B	6.14833	10.44459	8.0182
H	5.02774	10.77615	8.34434
B	6.70017	9.07837	9.07056
Br	8.05669	9.61661	10.51712
Br	5.09246	8.23237	9.97942
Br	7.64115	7.49781	8.11036

1-BI$_3$ (C_1): E_{tot} = -2237.73964109

P	5.71078	9.66727	6.29141
N	6.93253	12.7764	8.81493
C	7.14514	11.68212	8.11253
C	8.15566	13.66902	8.9598
C	7.96462	15.03125	8.30488
H	8.8713	15.61567	8.46292
H	7.13794	15.58696	8.74562
H	7.80933	14.96812	7.23154
C	8.51889	13.89596	10.4188
H	9.47902	14.41389	10.44607
H	8.63625	12.95884	10.95911
H	7.79583	14.52612	10.93353
C	9.22648	12.82684	8.27558
H	9.79838	12.27622	9.0254
H	9.92893	13.44076	7.70936
C	8.48207	11.82908	7.39751
C	9.30577	10.5704	7.18542
H	10.22623	10.83975	6.66308
H	8.79539	9.82793	6.5798
H	9.58209	10.09447	8.12188
C	8.17734	12.43439	6.01252
H	9.11947	12.61379	5.49266
H	7.63964	13.37896	6.05435
H	7.59483	11.75604	5.39203
C	5.70352	13.3411	9.35907
C	5.44884	13.31967	10.74361

C	6.04696	12.32195	11.70165
H	6.86715	11.80977	11.20908
C	4.98353	11.26758	12.01154
H	5.42073	10.4426	12.57388
H	4.55644	10.84835	11.10363
H	4.16931	11.69255	12.60308
C	6.57435	12.91258	13.00793
H	7.09047	12.13232	13.5682
H	5.77009	13.28138	13.64681
H	7.2772	13.73064	12.85809
C	4.48517	14.18039	11.26028
H	4.29504	14.16871	12.32523
C	3.76757	15.03602	10.45391
H	3.04386	15.71853	10.87927
C	3.95039	14.97133	9.08908
H	3.3389	15.59365	8.44856
C	4.88644	14.11891	8.51155
C	4.84412	14.0329	6.99824
H	5.57215	13.29374	6.66149
C	5.12786	15.35298	6.27917
H	5.1009	15.20393	5.19852
H	6.09566	15.77807	6.53089
H	4.37371	16.10436	6.51635
C	3.45055	13.54357	6.58577
H	3.40631	13.35102	5.51328
H	2.69092	14.2934	6.80696
H	3.17112	12.63523	7.11803
C	4.70224	10.81816	5.29868
H	5.23888	11.73885	5.07958
H	3.79392	11.06603	5.8385
H	4.42605	10.3457	4.35577
C	4.55128	8.28491	6.50629
H	5.05476	7.41264	6.91479
H	4.11829	8.03201	5.53788
H	3.76029	8.57138	7.19596
C	6.87246	9.06854	5.00966
H	7.53769	8.30474	5.39965
H	7.46567	9.8819	4.598
H	6.28171	8.6369	4.20076
B	6.20068	10.38857	8.06696
H	5.0863	10.68947	8.4394
B	6.80564	9.01938	9.08576
I	8.32709	9.57745	10.71493
I	5.05399	8.01104	10.12405
I	7.88396	7.25073	8.03912

1-AlCl₃ (C_1): E_{tot} = -2941.10952613

P	5.63728	9.68704	6.32074
N	6.92226	12.87652	8.76937
C	7.0743	11.78082	8.03154
C	8.1538	13.72944	8.89817
C	7.98218	15.0796	8.21025
H	8.91207	15.64142	8.30292
H	7.1964	15.67223	8.67788
H	7.7617	14.98945	7.14886
C	8.5323	13.9812	10.34985
H	9.48466	14.51357	10.36982
H	8.66354	13.05109	10.89944
H	7.80081	14.60312	10.86418
C	9.20065	12.85116	8.22183
H	9.75813	12.30021	8.98241
H	9.92187	13.43868	7.65123
C	8.43824	11.85235	7.3522
C	9.21597	10.54688	7.2698
H	10.17309	10.73578	6.77868
H	8.70689	9.77786	6.6991
H	9.42853	10.14106	8.25586
C	8.22772	12.39388	5.92836
H	9.19565	12.52069	5.44097
H	7.72014	13.35662	5.90471
H	7.64519	11.70537	5.31692
C	5.70381	13.4118	9.33305
C	5.42425	13.26566	10.70292
C	6.09187	12.25141	11.5991
H	6.9266	11.80161	11.06771
C	5.09176	11.13359	11.90029
H	5.58387	10.31299	12.42108
H	4.65153	10.7265	10.99312
H	4.27699	11.49519	12.53152
C	6.62089	12.82342	12.91408
H	7.18118	12.05176	13.44279
H	5.81229	13.1406	13.57488
H	7.283	13.67623	12.77395
C	4.3902	14.01638	11.25494
H	4.17499	13.91395	12.31059
C	3.62824	14.87323	10.49006
H	2.83988	15.45986	10.94297
C	3.85725	14.94291	9.13093
H	3.22208	15.57369	8.52192
C	4.87456	14.21403	8.5246
C	4.92898	14.26481	7.01126
H	5.77702	13.67459	6.66237
C	5.06169	15.67774	6.44013
H	5.1622	15.63616	5.35432
H	5.92268	16.21319	6.83379
H	4.1784	16.28053	6.65447
C	3.65532	13.6174	6.46086
H	3.70564	13.51339	5.37582
H	2.77758	14.22321	6.68931
H	3.49533	12.63429	6.90005
C	4.66197	10.6732	5.13012
H	5.20018	11.57581	4.84563
H	3.71727	10.96774	5.57885
H	4.45475	10.08837	4.2335
C	4.51249	8.31816	6.7267
H	5.06991	7.49638	7.17122
H	4.01712	7.96562	5.82214
H	3.76678	8.64686	7.44735
C	6.86404	8.91276	5.21373
H	7.48027	8.2062	5.76308
H	7.50052	9.65391	4.73537
H	6.3234	8.37461	4.43446
B	6.06844	10.61961	7.94598
H	4.97322	10.8511	8.40097
Al	6.83978	9.00522	9.28223
Cl	8.30851	9.69966	10.71998
Cl	5.14726	8.06335	10.22237
Cl	7.81179	7.39717	8.1782

1-GaCl₃ (C_1): E_{tot} = -4622.84169983

P	5.61687	9.66793	6.33897
N	6.92385	12.88202	8.75791
C	7.07706	11.78674	8.03015
C	8.16114	13.72993	8.89321
C	7.99658	15.08491	8.21454
H	8.92927	15.64062	8.31481
H	7.21286	15.6783	8.68444
H	7.7801	15.00397	7.15151
C	8.5331	13.96586	10.34891
H	9.48837	14.49251	10.37749
H	8.65685	13.02919	10.88917
H	7.80285	14.58731	10.86544
C	9.20529	12.85015	8.21394
H	9.76515	12.2995	8.97273
H	9.92364	13.43651	7.63872
C	8.43944	11.84998	7.3492
C	9.21806	10.54537	7.26034
H	10.16828	10.73898	6.75791
H	8.70681	9.77427	6.69438
H	9.44493	10.14072	8.24337
C	8.21781	12.39085	5.92596
H	9.18176	12.49914	5.42664
H	7.72738	13.36251	5.90563

H	7.61593	11.7105	5.32394
C	5.70491	13.41971	9.32257
C	5.41798	13.26186	10.68928
C	6.07691	12.23942	11.58239
H	6.91148	11.78599	11.05375
C	5.06929	11.1267	11.87813
H	5.55736	10.2995	12.39172
H	4.62367	10.72729	10.97023
H	4.25887	11.49008	12.514
C	6.60635	12.80338	12.90071
H	7.16137	12.02576	13.42605
H	5.79829	13.12219	13.56142
H	7.27382	13.65285	12.76571
C	4.38231	14.01154	11.23995
H	4.15965	13.90055	12.29313
C	3.62802	14.877	10.47731
H	2.83798	15.46147	10.93007
C	3.86659	14.95903	9.12046
H	3.2375	15.59716	8.51289
C	4.88593	14.23282	8.51464
C	4.95271	14.29882	7.00227
H	5.80475	13.71417	6.65404
C	5.08889	15.71768	6.44692
H	5.20163	15.68727	5.36198
H	5.94427	16.25067	6.85579
H	4.20202	16.31649	6.65735
C	3.68542	13.65503	6.43343
H	3.7482	13.55656	5.34845
H	2.80534	14.25992	6.65518
H	3.51931	12.66984	6.86505
C	4.61039	10.64338	5.16821
H	5.13544	11.5484	4.86756
H	3.67287	10.9325	5.63574
H	4.38841	10.05273	4.2791
C	4.52049	8.28677	6.7753
H	5.1007	7.47295	7.20546
H	4.00745	7.92793	5.8831
H	3.78936	8.60527	7.5152
C	6.84142	8.90653	5.22345
H	7.46557	8.2063	5.77304
H	7.46848	9.65274	4.74062
H	6.30018	8.36209	4.44894
B	6.05885	10.61968	7.95899
H	4.963	10.87748	8.39749
Ga	6.84622	9.0479	9.26694
Cl	8.37355	9.74041	10.72617
Cl	5.17435	8.02774	10.27464
Cl	7.85026	7.38256	8.16573

1-InCl$_3$ (C_1): E_{tot} = -2889.10216462

P	5.59711	9.66698	6.34797
N	6.9154	12.91877	8.7393
C	7.06452	11.82041	8.01487
C	8.15578	13.75882	8.88114
C	8.00032	15.11649	8.20535
H	8.93423	15.66847	8.31397
H	7.2163	15.71098	8.67336
H	7.79052	15.04132	7.1402
C	8.52156	13.98798	10.33982
H	9.47426	14.51872	10.37557
H	8.64824	13.04879	10.87545
H	7.7862	14.60347	10.85655
C	9.19842	12.87503	8.20316
H	9.76099	12.32988	8.96389
H	9.91484	13.45867	7.62291
C	8.43228	11.86865	7.34357
C	9.20302	10.55728	7.2838
H	10.15409	10.73024	6.77577
H	8.68857	9.77264	6.7393
H	9.43549	10.18028	8.27769
C	8.2238	12.39354	5.91283
H	9.19047	12.48786	5.41615
H	7.74262	13.3698	5.88184
H	7.61672	11.71305	5.31587
C	5.69437	13.44472	9.30495
C	5.39446	13.24693	10.6627
C	6.0666	12.2156	11.53562
H	6.91179	11.78787	11.00057
C	5.07715	11.08233	11.81359
H	5.57386	10.26041	12.32749
H	4.6393	10.68315	10.90094
H	4.2556	11.42412	12.44675
C	6.58923	12.76235	12.86374
H	7.1665	11.98662	13.3672
H	5.77636	13.04683	13.53412
H	7.23455	13.63079	12.74348
C	4.33996	13.96578	11.21842
H	4.10579	13.82585	12.26571
C	3.58119	14.83689	10.46598
H	2.77483	15.39578	10.92218
C	3.83672	14.95934	9.11478
H	3.20546	15.60285	8.51515
C	4.8767	14.26626	8.50567
C	4.9727	14.36968	6.99728
H	5.8501	13.82019	6.65543
C	5.07977	15.80493	6.48013
H	5.21966	15.8055	5.39799
H	5.90996	16.35026	6.92371
H	4.17135	16.37312	6.68327
C	3.74169	13.69786	6.38418
H	3.8335	13.62537	5.29903
H	2.83613	14.26702	6.5982
H	3.60074	12.6981	6.7902
C	4.56605	10.59432	5.16032
H	5.08017	11.49414	4.82702
H	3.63036	10.88954	5.6285
H	4.3413	9.97401	4.29247
C	4.54087	8.27289	6.84029
H	5.15564	7.46953	7.24379
H	3.99365	7.89785	5.97557
H	3.83839	8.5789	7.61288
C	6.83785	8.89773	5.25986
H	7.46033	8.21086	5.83051
H	7.46494	9.63877	4.76935
H	6.30924	8.33298	4.49121
B	6.02781	10.67592	7.93179
H	4.9401	10.91703	8.39183
In	6.90516	9.01365	9.35649
Cl	8.5783	9.80462	10.90376
Cl	5.13106	7.83733	10.44459
Cl	7.98821	7.22723	8.13085

References

[1] M. Arrowsmith, J. D. Mattock, J. Böhnke, I. Krummenacher, A. Vargas, H. Braunschweig, *Chem. Commun.* **2018**, *54*, 4669–4672.

[2] I. Chavez, A. Alvarez-Carena, E. Molins, A. Roig, W. Maniukiewicz, A. Arancibia, V. Arancibia, H. Brand, J. Manuel Manriquez, *J. Organomet. Chem.* **2000**, *601*, 126–132.

[3] I. Noviandri, K. N. Brown, D. S. Fleming, P. T. Gulyas, P. A. Lay, A. F. Masters, L. Phillips, *J. Phys. Chem. B*, **1999**, *103*, 6713-6722.

[4] S. Stoll, A. Schweiger, *J. Magn. Reson.* **2006**, *178*, 42–55.

[5] G. Sheldrick, *Acta Cryst.* **2015**, *A71*, 3–8.

[6] G. Sheldrick, *Acta Cryst.* **2008**, *A64*, 112–122.

[7] A. L Spek, *Acta Cryst.* **2015**, *C71*, 9–18.

[8] F. Neese, *Wiley Interdiscip. Rev. Comput. Mol. Sci.* **2018**, *8*, e1327.

[9] S. Grimme, J. G. Brandenburg, C. Bannwarth, A. Hansen, *J. Chem. Phys.* **2015**, *143*, 054107.

[10] S. Kozuch, D. Gruzman, J. M. L. Martin, *J. Phys. Chem. C* **2010**, *114*, 20801-20808.

[11] S. Grimme, S. Ehrlich, L. Goerigk, *J. Comput. Chem.* **2011**, *32*, 1456-1465.

[12] F. Weigend, R. Ahlrichs, *Phys. Chem. Chem. Phys.* **2005**, *7*, 3297-3305.

[13] F. Weigend, *Phys. Chem. Chem. Phys.* **2006**, *8*, 1057-1065.

[14] A. Hellweg, C. Hättig, S. Höfener, W. Klopper, *Theor. Chem. Acc.* **2007**, *117*, 587-597.

[15] S. Ten-no, *Chem. Phys. Lett.* **2004**, *398*, 56-61.

[16] T. B. Adler, G. Knizia, H.-J. Werner, *J. Chem. Phys.* **2007**, *127*, 221106.

[17] H..-J. Werner, P. J. Knowles, G. Knizia, F. R. Manby, M. Schütz, *Wiley Interdiscip. Rev. Comput. Mol. Sci.* **2012**, *2*, 242-253.

[18] K. A. Peterson, T. B. Adler, H.-J. Werner, *J. Chem. Phys.* **2008**, *128*, 084102.

[19] F. Weigend, *J. Comput. Chem.* **2008**, *29*, 167-175.

[20] F. Weigend, A. Köhn, C. Hättig, *J. Chem. Phys.* **2002**, *116*, 3175-3183.

[21] K. E. Yousaf, K. A. Peterson, *Chem. Phys. Lett.* **2009**, *476*, 303-307.

[22] G. te Velde, F. M. Bickelhaupt, E. J. Baerends, C. Fonseca Guerra, S. J. A. van Gisbergen, J. G. Snijders, T. Ziegler, *J. Comput. Chem.* **2001**, *22*, 931-967.

[23] ADF2019, SCM, T. Chemistry, V. Universiteit, Amsterdam, T. Netherlands, http://www.scm.com, **2019**.

[24] A. D. Becke, *Phys. Rev. A* **1988**, *38*, 3098-3100.

[25] J. P. Perdew, *Phys. Rev. B* **1986**, *33*, 8822-8824.

[26] E. Van Lenthe, E. J. Baerends, *J. Comput. Chem.* **2003**, *24*, 1142-1156.

[27] T. Lu, F. Chen, *J. Comput. Chem*. **2012**, *33*, 580-592.

[28] C. Y. Legault, Université de Sherbrooke, http://www.cylview.org, **2009**.

[29] G. A. Andrienko, https://www.chemcraftprog.com, **2015**.
